# ProZES: the methodology and software tool for assessment of assigned share of radiation in probability of cancer occurrence

**DOI:** 10.1007/s00411-020-00866-7

**Published:** 2020-08-26

**Authors:** Alexander Ulanowski, Elena Shemiakina, Denise Güthlin, Janine Becker, Dale Preston, A. Iulian Apostoaei, F. Owen Hoffman, Peter Jacob, Jan Christian Kaiser, Markus Eidemüller

**Affiliations:** 1 Institute of Radiation Medicine, Helmholtz Zentrum München, Ingolstädter Landstraße 1, 85764 Neuherberg, Germany; 2 Present Address: IAEA Environment Laboratories, International Atomic Energy Agency, 2444 Seibersdorf, Austria; 3 Present Address: Department of Radiation Protection and Health, Federal Office for Radiation Protection, 85764 Oberschleissheim, Germany; 4 Hirosoft International, Eureka, CA USA; 5 Oak Ridge Center for Risk Analysis, Inc, Oak Ridge, TN USA

**Keywords:** Radiation exposure, Radiation risk, Risk analysis, Malignant neoplasms

## Abstract

ProZES is a software tool for estimating the probability that a given cancer was caused by preceding exposure to ionising radiation. ProZES calculates this probability, the assigned share, for solid cancers and hematopoietic malignant diseases, in cases of exposures to low-LET radiation, and for lung cancer in cases of exposure to radon. User-specified inputs include birth year, sex, type of diagnosed cancer, age at diagnosis, radiation exposure history and characteristics, and smoking behaviour for lung cancer. Cancer risk models are an essential part of ProZES. Linking disease and exposure to radiation involves several methodological aspects, and assessment of uncertainties received particular attention. ProZES systematically uses the principle of multi-model inference. Models of radiation risk were either newly developed or critically re-evaluated for ProZES, including dedicated models for frequent types of cancer and, for less common diseases, models for groups of functionally similar cancer sites. The low-LET models originate mostly from the study of atomic bomb survivors in Hiroshima and Nagasaki. Risks predicted by these models are adjusted to be applicable to the population of Germany and to different time periods. Adjustment factors for low dose rates and for a reduced risk during the minimum latency time between exposure and cancer are also applied. The development of the methodology and software was initiated and supported by the German Federal Ministry for the Environment, Nature Conservation and Nuclear Safety (BMU) taking up advice by the German Commission on Radiological Protection (SSK, Strahlenschutzkommission). These provide the scientific basis to support decision making on compensation claims regarding malignancies following occupational exposure to radiation in Germany.

**Electronic supplementary material:**

The online version of this article (10.1007/s00411-020-00866-7) contains supplementary material, which is available to authorized users.

## Introduction

Ionising radiation is known to induce or contribute to long-term health effects. If a person with a history of exposure to radiation is diagnosed with cancer, it is possible that the disease is related to the preceding exposure. Since cancer induction and promotion by ionising radiation is a fundamentally stochastic process, the relationship between radiation and cancer can only be expressed by probabilities. The probability that the observed cancer in an exposed person may be caused by past exposure to radiation is called the assigned share. The assigned share is derived based on risk models obtained from radioepidemiological studies and depends on type of cancer, age at cancer occurrence, exposure history, and other personal factors. ProZES^1^ is a computer program that was developed to quantitatively assess the assigned share together with related uncertainties. This paper describes the background, methodology and risk models of ProZES.

Persons who develop cancer after occupational exposure to ionising radiation may get compensated in their national countries. Principles and implementations of compensation systems vary among countries (ILO 2010). In Germany, judicial decisions on eligibility for compensation are based on estimates of assigned share. In 2009, the German Federal Ministry for the Environment, Nature Conservation and Nuclear Safety (BMU) initiated and supported the development of ProZES, taking up advice by the German Commission on Radiological Protection (SSK, Strahlenschutzkommission), to replace special radioepidemiological tables (Chmelevsky et al. 1995) that are in use for compensation claims in Germany. The aim was to develop a modern interactive software tool capable of calculating the assigned share based on scientific state-of-the-art risk models.

The functionality of ProZES is in many aspects similar to the US online tool IREP^2^ (Land et al. 2003; Kocher et al. 2008) which is used for adjudication on compensation claims in the United States. However, ProZES has been developed independently, following critical revisions of methodology and models. Progress on the project was regularly presented to a dedicated working group of the German Commission on Radiological Protection (SSK), and to the Federal Office for Radiation Protection (BfS) who is the owner of the program. Furthermore, methodological aspects and risk models were discussed in depth with an external group of international experts.

Assessment of the assigned share is a complex process that includes several methodological challenges. Risk models obtained from radioepidemiological cohorts must be applied to a current Western population. Dose and dose rate exposure conditions for such cohorts are often different from exposures used in applications of ProZES and thus, the risks obtained from radioepidemiological studies require adjustments. Since selection of a single “best” model is often not possible, ProZES makes systematic use of the principle of multi-model inference (Burnham and Anderson 2002). Due to power limitations of epidemiological data, it is possible to develop dedicated risk models only for the most frequently occurring cancers. Reasonable grouping of other cancer sites is necessary to construct meaningful and reliable risk models. The use of models with non-linear dose response relationships requires special attention in cases of fractionated or protracted exposures.

ProZES allows for estimation of assigned share after low-LET exposure for almost all types of solid cancer and hematopoietic malignancies. The low-LET risk models are largely based on the Life Span Study (LSS) of the atomic bomb survivors of Hiroshima and Nagasaki (Preston et al. 2007), and most models have been newly developed from cohort data using the ProZES methodology. In addition, the program includes risk models for lung cancer after radon exposure. Uncertainties from various sources are assessed and reported as a probability distribution of the assigned share from which the confidence intervals can be obtained.

This paper presents the methodology and risk models developed and implemented in ProZES. The first part introduces the general framework for expressing the relationship between radiation and disease. The following part discusses the framework of the radiation risk models, including design principles, grouping of cancer sites, and selection and fitting procedures of the models. The most important properties of the derived risk models are presented. The paper concludes with a discussion and summary. The electronic supplement contains detailed information on the risk models including model parameter values with uncertainties and covariance matrices. Additional documentation can be found in the ProZES reports (Jacob et al. 2013; Ulanowski et al. 2016). The ProZES webpage (BfS 2020) contains further information on current developments and includes a link for downloading the software.

## Expressing the relation between radiation and disease

### Assigned share

The central quantity in ProZES is the assigned share for a diagnosed primary malignancy. The assigned share depends on the total risk of developing the malignancy, given the radiation exposure history of an individual. Assuming that the risk to the individual can be represented by the disease incidence rates observed in a population matching the individual by age, sex, country, birth year and exposure history, the assigned share can be interpreted as a measure of the probability that the diagnosed malignancy was caused by the past exposure to radiation: hence probability of causation. The assigned share of radiation, hereafter denoted as *Z*, can be expressed as follows:

1 $$Z = \frac{h}{\lambda } = \frac{h}{{\lambda_{0} + h}} ,$$

where for the given disease diagnosed at age *a*, $\lambda$ is the total incidence rate of the disease in the matching hypothetical exposed population, in cases per person year (PY^−1^); $\lambda_{o}$ is the observed baseline incidence rate in the matching non-exposed population (PY^−1^); and $h$ represents the inferred excess incidence rate reflecting the impact of radiation exposures (PY^−1^). For positive values of the excess rate $h$, the assigned share has values between zero and one.

As follows from this definition, *Z* is a ratio of two quantities described by the baseline and the excess rates. In ProZES, uncertainties of both quantities are simulated to derive the uncertainty in *Z*. Probability distribution functions are used to model uncertainty in all involved quantities.

Conventionally used alternative formulas, $Z = {\text{EAR}}/\left( {\lambda_{0} + {\text{EAR}}} \right) = {\text{ERR}}/\left( {1 + {\text{ERR}}} \right)$, represent the assigned share in terms of either excess absolute rate (or excess absolute risk) ${\text{EAR}} = h$, or excess relative risk ${\text{ERR}} = h/\lambda_{0}$ (Land et al. 2003). However, as discussed in the next section, the mechanism of risk transfer between populations involves different baseline rates, and the type of transfer is not identical to the type of phenomenological model selected to quantify radiation risk in the epidemiological cohort. Therefore, ProZES always uses Eq. (1) for the calculation of *Z*.

### Transfer of risk between populations

Excess and baseline rates in Eq. (1) need to be pertinent to the target population. However, the radiation risk models are derived from epidemiological studies of certain population groups exposed to radiation. Commonly, the studied epidemiological cohorts differ from the target population with respect to ethnicity, lifestyle, dietary, occupational, temporal, geographic, and other features. Therefore, risk estimates derived from epidemiological studies need to be adjusted or “transferred” to become applicable to the target population.

Ideas for modelling the risk transfer from one population to another can be developed based on current notions of carcinogenic effects of radiation (NRC 2006; UNSCEAR 2012). Carcinogenesis is a complicated multi-stage process and radiation is thought to influence various stages of tumour development (Rühm et al. 2017). Different processes can be induced by ionising radiation, for example:

- Creation of pre-malignant cells with a growth advantage from healthy stem or progenitor cells, e.g. by induction of driver gene mutations. Such initiating processes might be more closely related to additive risk transfer.
- Growth acceleration of clones of pre-malignant cells. Its magnitude depends on the number of pre-malignant cells and might be more closely related to multiplicative risk transfer.

Correspondingly, a transfer mode can be selected to be either additive, when the excess absolute rate from a given dose in the target population, $h$, is assumed to be the same as the modelled excess absolute rate, $h_{\text{m}}$, for the same dose in the studied radioepidemiological cohort or multiplicative, when the same is assumed for the excess relative risk for the target population, $h/\lambda_{0}$, and the modelled excess relative risk, $h_{\text{m}} /\lambda_{{0{\text{m}}}}$, for the same dose. The resulting excess rate for the target population can, therefore, be modelled as a weighted sum of excess rates predicted by either transfer mode, additive or multiplicative. The excess rate in the target population can then be expressed as follows:2where $f$ is the relative weight of the multiplicative transfer, $1 - f$ is the complementary weight of the additive one, $h_{\text{m}}$ and $\lambda_{{ 0 {\text{m}}}}$ are the model excess and baseline incidence rates, correspondingly. Thus, $f = 1$ results in a pure multiplicative transfer, $f = 0$ in a pure additive transfer, and values in the range $0 < f < 1$ are representative for a mixed transfer mode.

Denoting the ratio of the target population baseline and the model baseline rates as $B = \lambda_{0} /\lambda_{0m}$, the Eq. (2) can be re-written as follows:

3 $$h = h_{\text{m}} \left( {1 - f + fB} \right)$$

and, correspondingly, the assigned share (1) as:

4 $$Z = \frac{{h_{\text{m}} \left( {1 - f + fB} \right)}}{{\lambda_{0} + h_{\text{m}} \left( {1 - f + fB} \right)}} .$$

The baseline ratio $B$ reflects differences that exist between the spontaneous incidence rate in the target population and the model-based estimate of the baseline rate derived from the epidemiological data. Besides potential differences in ethnicity and lifestyle of the populations, differences also arise from the fact that the cohort-specific model estimates and the disease rates for the target population are frequently related to different time periods. Varying time trends in the disease incidence rates may therefore significantly influence *B*. For example, assume that some cancer of a German person was observed in the year 2010, 30 years after an exposure in 1980. When using a LSS-based model, the calendar year in the LSS cohort corresponding to a time since exposure of 30 years is 1975 (i.e., 30 years after the detonation of the atomic bomb in 1945), so $B = \lambda_{{ 0 , {\text{Ger}}}} \left( {2010} \right) /\lambda_{{ 0 , {\text{LSS}}}} \left( {1975} \right).$

Selection of the coefficient $f$, which defines relative weights of either type of risk transfer, is a challenging task. A convincing decision about the type of risk transfer for a specific cancer should not only be based on descriptive risk modelling, but also include additional biological and mechanistic information (UNSCEAR 2012). If no preferred value or range of values can be identified for $f$, then any value in the range from zero to one can be regarded as equally likely, thus indicating equal probabilities to all combinations between additive and multiplicative terms (Eq. 2). Under such circumstances, generation of the distribution of the assigned share can be realised by sampling a value of $f$ from a uniform distribution: $f\sim U\left( {0,1} \right)$, which represents the situation of highest uncertainty (maximum entropy). Correspondingly, this sampling method is implemented in the current version of ProZES for the value of $f$. For simplified comparisons, the best estimates of $h$ and $Z$ can be calculated using the mean value of the factor $f = 0.5$. For example, the latter simplified approach was applied in the WHO report on health consequences of the Fukushima accident (WHO 2013). Importantly, if baseline rates are significantly different, then a significant part of the uncertainty range of the assigned share calculated in ProZES can originate from the uncertainty associated with the transfer factor $f$.

### Multi-model inference

A distinctive feature of the current development is a systematic use of multi-model inference (MMI) (Burnham and Anderson 2002; Anderson 2008; Claeskens and Hjort 2008). Since no single true risk model exists, different plausible alternative models can be used to approximate the underlying risk. Statistical modelling can be performed using several models differing by number and type of model parameters. Different models often provide statistically similar quality of fit and cannot be rejected based on purely statistical criteria (e.g. by likelihood ratio tests or information criteria). In MMI, instead of selecting only one “best” model and neglecting all other models, the risk is calculated based on several plausible models accounting for their descriptive capabilities. Thus, risk estimates are less dependent on the choice of one particular model. Typically, model predictions agree well in the centre of the data space but diverge on the fringes. Hence, MMI improves the characterisation of uncertainties in regions of the data space with fewer numbers of cases and weaker statistical evidence.

Practical implementation of the MMI principle begins with the selection of a set of plausible models describing the epidemiological data similarly well. Then, each model *i* is assigned a weight $w_{i}$ with $\sum\nolimits_{i} {w_{i} = 1}$. A distribution of assigned share is constructed from a stochastically generated sample created by merging sub-samples generated by the different models using their corresponding weights. For example, to estimate risk and error bounds from 10,000 realizations for 2 models with 60% and 40% weights, 6000 and 4000 realizations are performed for each model and, for each realization, the assigned share values are computed. The resulting percentiles of the combined sample of the assigned share values produced by these models include both the uncertainty from the model selection and the model-specific statistical parameter uncertainties.

One possible way to select the model weights $w_{i}$ is to use the Akaike Information Criterion, given by ${\text{AIC}} = {\text{dev}} + 2n$, where dev is the model deviance and $n$ is the number of model parameters. The weights are obtained from

5 $$w_{i} = \frac{{{\text{e}}^{{ - \frac{1}{2}\Delta {\text{AIC}}_{i} }} }}{{\mathop \sum \nolimits_{k = 1}^{M} {\text{e}}^{{ - \frac{1}{2}\Delta {\text{AIC}}_{k} }} }} .$$

The sum runs over all $M$ models, and $\Delta {\text{AIC}}_{i}$ is the difference of the Akaike value of model $i$ to some reference value, e.g. the minimum Akaike value. AIC-based MMI has been applied for radiation risk estimates by Kaiser et al. (2012), Kaiser and Walsh (2013) and Schöllnberger et al. (2018). Also, in these references, the results from the MMI approach are compared to the standard approach with a single risk model.

### Dose rate effectiveness factor (DREF)

The concept of a dose- and dose rate effectiveness factor (DDREF) in carcinogenesis has been widely and controversially discussed during the last decade (Rühm et al. 2015; Shore et al. 2017; Kocher et al. 2018). The use of a DDREF to transfer risk quantities obtained from epidemiological studies at moderate and high doses and dose rates to low doses and dose rates typical for occupational exposures has been recommended by NRC (2006) and ICRP (2007). For example, the program IREP (Kocher et al. 2008) follows this approach for risk calculations.

In ProZES, an alternative method has been implemented following UNSCEAR (2018, Annex B) and recommendations of the German Commission on Radiological Protection (SSK 2014) observing that studies of cancer risk due to radiation exposure at low dose rates and moderate doses do not provide evidence for lower risks per unit exposure than studies of high radiation doses and dose rates. The method is based on results of Jacob et al. (2009) and follows UNSCEAR (2012, Annex B, Paragraph C45), so the risk estimates are only modified by a dose rate effectiveness factor (DREF). The DREF distribution is approximated by a log-normal probability distribution with the constant geometric mean ${\text{GM}} = 1$ and the geometric standard deviation GSD, which depends on dose rate. Mathematically, the geometric standard deviation is represented by a log-linear decreasing function of the dose rate in the low-dose rate range, i.e., below a threshold dose rate of 0.1 mGy min^−1^ = 6 mGy h^−1^ (UNSCEAR 2000; ICRP 2005): ${\text{GSD}}\left( {\text{dr}} \right) = { \hbox{max} }\left( {1.0, 1.1803 - 0.2317\log_{10} \left( {\text{dr}} \right)} \right)$, where ${\text{dr}}$ represents the dose rate in mGy h^−1^ (Jacob et al. 2013). For dose rates exceeding the threshold, GSD is set equal to 1.0. The selected parameters ensure that at dose rates that are typical for elevated occupational dose rates (1 mGy d^−1^ ≈ 0.042 mGy h^−1^) the results correspond to Jacob et al. (2009), i.e. $GSD = 1.5$.

**Fig. 1:**
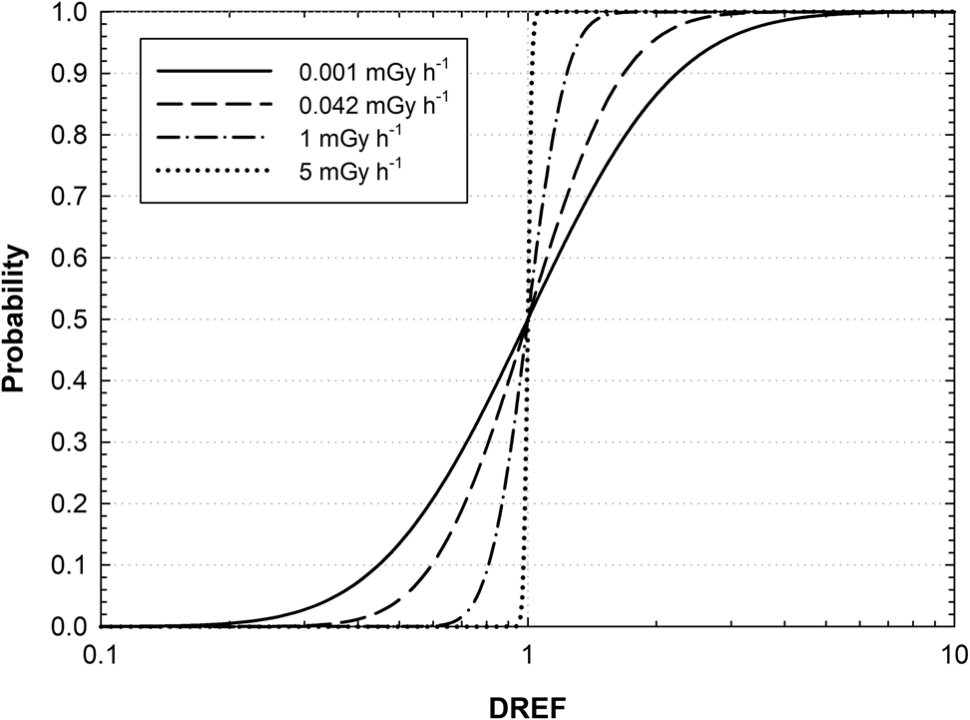
Cumulative probability distributions of DREF for different dose rates as implemented in ProZES

As shown in Fig. 1, the resulting cumulative probability distribution of the DREF becomes broader when the dose rate decreases. A comparison of DDREF distributions from BEIR VII (NRC 2006), Kocher et al. (2008) and Jacob et al. (2009) can be found in UNSCEAR (2012, Annex B, Fig. C-II). Reduction of the dose rate does not change the median value of the DREF, which remains equal to 1.0, and, correspondingly, the median of the risk estimates is not affected, as well. However, uncertainty bounds of the risk estimates become larger when the dose rate decreases, thus reflecting the decreasing evidence of risk inference at such conditions.

### Minimum latency period

Due to the multi-stage process of cancer development, radiation-induced cancer can be diagnosed only after a minimum period necessary for development of the disease, or latency time. Therefore, in ProZES, a multiplicative latency-adjustment factor is implemented to reduce risk estimates at early times since exposure. A widely adopted technique (Land et al. 2003; Kocher et al. 2008; Berrington de Gonzalez et al. 2012) is to use an S-shaped function to gradually reduce risk estimates at times since exposure shorter than the observed period of latency. Unlike alternative implementation realised in IREP (Kocher et al. 2008) and RadRAT (Berrington de Gonzalez et al. 2012), the following asymmetric form of the S-shaped latency factor was adopted for use in ProZES ensuring zero radiation risk for vanishing time after exposure:

6 $$F_{\text{L}} = \left[ {1 + \exp \left( { - \eta \ln \left( {\frac{t}{{t_{0} }}} \right)} \right)} \right]^{ - 1} ,$$

where $t_{0}$ is the central estimate (median) of the latency period (year); $t$ is the time since exposure (year); and $\eta$ is the parameter controlling the width of the transition period from zero to full probability.

**Fig. 2:**
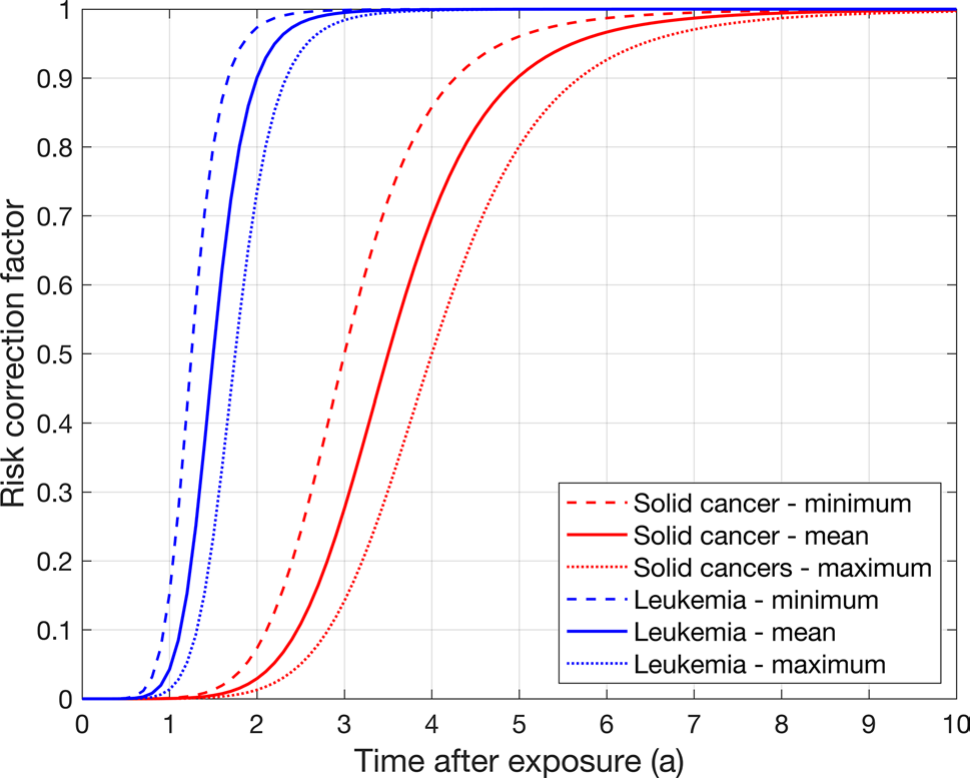
Risk adjustment factor to account for latency time

In the software tool ProZES, the latency factor (6) is modelled as a random function with parameters different for solid and hematopoietic cancers, as shown in Fig. 2. Selection of minimum latency times is based on inferences from studies on leukaemia in the LSS cohort (Lange et al. 1954) and members of the public after the Chernobyl accident (Noshchenko et al. 2002); and from studies on solid cancers from radiotherapy patients (Berrington de Gonzalez et al. 2011), nuclear workers (Daniels et al. 2017), Chernobyl emergency workers (Ivanov et al. 2009), and studies of thyroid cancer after the Chernobyl accident (Heidenreich et al. 1999; Williams et al. 2004). For solid cancers, the parameter $t_{0}$ is sampled uniformly in the range from 3 to 4 years and the width parameter is taken equal to $\eta = 6.25$; for leukaemia and lymphomas, $t_{0}$ is sampled uniformly in the range from 1.25 to 1.75 years with a width parameter of $\eta = 7.66$. Thus, in practice, only risk estimates with a latency time of less than about 6 years for solid cancers, and about 2 years for leukaemia and lymphomas are affected.

### Multiple exposures

Generally, a person may be exposed by radiation not only once, but several times. The evaluation of the assigned share then involves an assessment of the combined effect of all exposures. Taking into account that organ-specific cancer incidence rates are small on an absolute scale (as a rule, not exceeding 10^−2^ PY^−1^ for the most frequently occurring cancers and the highest personal ages), and assuming that effects of different exposures on carcinogenesis are independent, the cumulative effect of a series of exposures can be represented as a sum of excess rates from single exposures:

7 $$\lambda = \lambda_{0} + \mathop \sum \limits_{i = 1}^{n} h_{i} ,$$

where $h_{i}$ is the excess rate at diagnosis age *a* due to the *i*th exposure at age *e*_*i*_, in a series of *n* exposures. Consequently, the assigned share from multiple exposures is given by

8 $$Z = \frac{{\mathop \sum \nolimits_{i} h_{i} }}{{\lambda_{0} + \mathop \sum \nolimits_{i} h_{i} }} .$$

Accounting for mixed risk transfer (3), and assuming that the transfer parameter *f* is the same for all exposures, the assigned share is obtained from the following expression:

9 $$Z = \frac{{\left( {1 - f} \right)\mathop \sum \nolimits_{i} h_{{{\text{m}}.i}} + f\mathop \sum \nolimits_{i} h_{{{\text{m}},i}} B_{i} }}{{\lambda_{0} + \left( {1 - f} \right)\mathop \sum \nolimits_{i} h_{{{\text{m}}.i}} + f\mathop \sum \nolimits_{i} h_{{{\text{m}},i}} B_{i} }} ,$$

where $h_{{{\text{m}},i}}$ is the model excess rate of the *i*th exposure, $\lambda_{{0{\text{m}},i}}$ is the model baseline rate for conditions specific to the *i*th exposure, and $B_{i} = \frac{{\lambda_{0} }}{{\lambda_{{0{\text{m}},i}} }}$ is the baseline rates’ ratio.

### Extrapolations of risk beyond the time period supported by the epidemiological data

Risk estimates derived from radioepidemiological studies usually have good statistical support in a parameter domain specified by sex, age and exposure conditions of the studied cohort members. Outside this domain, the model risk estimates lose support from the epidemiological data and become more uncertain. For the LSS cohort (Preston et al. 2007), the follow-up for solid cancer incidence starts from 1958 and continues until 1998 for most of the models implemented in ProZES. Consequently, estimates of radiation risk of cancer incidence are not available within the first 13 years after exposure and for more than 53 years after exposure. Age dependencies of the assigned share can arise not only through the dependence of the ERR and EAR on age directly, but also through temporal trends in the population baseline rates.

To avoid potential problematic extrapolations due to such long-term trends, it was decided to fix the assigned share at time since exposure of 53 years and apply this value for time since exposure exceeding 53 years. For the early period during 13 years after exposure, no such restriction was imposed for solid cancers. The derived model functions were allowed to extrapolate towards zero time since exposure, because the period of latent development of a solid cancer is effectively accounted for by the latency factor at times since exposure below 5 years; thus the excess rates and, correspondingly, the assigned share are properly reduced.

A similar procedure was used for hematopoietic malignancies. Here, the follow-up runs from 1950 to 2001, so the assigned share is set constant for times after exposure exceeding 56 years. Due to reduced support of the risk models for hematopoietic diseases with fewer observed cases and, correspondingly, weaker statistical significance of the risk parameters describing the dependence on time since exposure, no extrapolation towards zero time since exposure was allowed for the model functions for hematopoietic diseases at small times since exposure, and a constant risk value was used corresponding to the model estimates at 5 years since exposure.

### Uncertainty modelling

An essential element of ProZES is a systematic evaluation of the uncertainty of the assigned share. Monte Carlo sampling is used to estimate the total distribution of *Z* from several sources of uncertainties. Starting the calculation, ProZES runs a certain number of iterations (current default value is 5000 with a maximum of 50,000). For each iteration, a risk model is randomly selected from the implemented list of models corresponding to the specific organ in proportion to their MMI weights $w_{i}$. Then, the parameters of the chosen model, both for baseline and radiation risk, are sampled under normality assumptions using multivariate Gaussian distributions with the parameter values and the full covariance matrix derived from the maximum likelihood fit of the epidemiological data. Further sampling for each iteration includes the parameters of the latency factor $F_{\text{L}}$, the transfer factor to the target population $f$, the DREF percentile, and the background incidence rate of the target population. For LSS-based models, factors are sampled to account for uncertainties in dosimetry and the radiation effectiveness factor for neutrons, as described below. These parameters are considered individual specific, so they are sampled in each new iteration remaining fixed within the iteration. The contribution of uncertainty of the target population’s baseline rate to the uncertainty of the assigned share can become large for cancers occurring with low rates, e.g., for rare diseases or diseases in young age, or in small populations. This uncertainty is assessed assuming a Poisson distribution for the number of registered cancer cases in the respective age–sex group of the target population. The combined contributions of multiple exposures to the excess rate are calculated according to Eq. (7). For each exposure within a single iteration, the value of dose is sampled from the user-defined uncertainty distribution, and the individual-specific latency factor and the DREF are applied using the time since exposure and dose rate corresponding to the specific exposure. Finally, *Z* is calculated in each iteration using Eq. (9). The mean, median and percentiles of *Z* are determined from the resulting distribution of *Z* values across all iterations.

### Cancer incidence and demographic data

The default target population of ProZES is the population of the Federal Republic of Germany. The primary source of cancer incidence data is the publicly available database of the “Zentrum für Krebsregisterdaten” (ZfKD) hosted by the Robert Koch Institute (RKI 2020). Cancer incidence rates, grouped according to their ICD10 codes, have been collected and included in the program. Currently, the data set covers the period from 1995 to 2014, with rates being provided for each year. The ZfKD database does not include country-average incidence rates of non-melanoma skin cancer (ICD10: C44). For this cancer type, the program uses incidence rates for the period from 2002 to 2014 that were obtained from the Bavarian cancer registry (LGL 2020). Demographic data for the period from 1980 to 2015 were obtained from the publicly available dataset of the German Federal Statistical Office (DESTATIS 2020). Risk computations for individuals diagnosed in later years, for which the cancer incidence and demographic data are not yet available, are performed using cancer rates from the most recent available year. It is planned to periodically update the cancer incidence and demographic data.

## Framework of radiation risk models

### Design principles for radiation risk models

The greatest amount of effort during the development of ProZES was dedicated to the development and evaluation of the radiation risk models. A decision was made by German authorities that the types of risk models in the current version of ProZES should include only classic, descriptive models of excess relative risk (ERR) and excess absolute rate (EAR). Alternatively, radiation risks can be estimated from epidemiological data using biologically based risk models that have an advantage of accounting for the process of carcinogenesis (Rühm et al. 2017). However, these biologically based models are not included in the current version of ProZES, because, at the time when the ProZES development started, concerns were raised whether these mechanistic models would be accepted widely enough to allow for their use in compensation claims.

In ProZES, the model type (ERR- or EAR-type) is explicitly distinguished from multiplicative or additive types of risk transfer (Eq. 2). Either type of model includes description of excess and baseline rates necessary for risk transfer and estimation of the assigned share.

Risk models for low-LET radiation are mostly based on the Life Span Study (LSS) of the atomic bombing survivors in Hiroshima and Nagasaki (Preston et al. 2007; Grant et al. 2017). With its large number of cohort members, wide range of exposures, and long and high-quality follow-up, the LSS cohort is the most important source of epidemiological evidence on risk of radiation-attributed diseases. The LSS is the only cohort where reliable risk models for many specific and grouped cancer sites can be derived also allowing for selection of statistically significant modifiers of radiation risk, e.g., related to sex, age, time and screening. For breast cancer, several large medical cohorts exist (e.g. Swedish hemangioma cohorts, Lundell et al. 1999), and a study of pooled data from these cohorts by Preston et al. (2002) has been used.

A recent pooled study of large nuclear worker cohorts has estimated the risk of death for all solid cancer and leukaemia (Richardson et al. 2015, 2018; Leuraud et al. 2015; Daniels et al. 2017). However, average doses and, consequently, the number of radiation-attributed cases are much lower than in the LSS, so site-specific risk estimates have large uncertainties (Richardson et al. 2018; Muirhead et al. 2009) and are not implemented in the current version of ProZES.

For lung cancer after radon exposure, it was decided to implement two different risk models, depending on the exposure situation: one model for mining and other underground work, and another for indoor, residential exposure. The miner model was obtained from the German Wismut cohort (Kreuzer et al. 2015). It is the world’s largest cohort of miners occupationally exposed to radon and includes members of the German population. The model for indoor exposures was obtained from results of the study of Darby et al. (2005).

The selection of models and parameter estimation required some general design decisions. The overall aim was to obtain robust, “evidence-based” risk models, that are scientifically supported by epidemiological data. To avoid researchers’ subjective preferences for some particular model or form of effect modification (such as the age dependence of the excess risk), model selection and parameter estimation was guided by the following principles which were applied consistently during the development of the ProZES risk models:

1. No common dependence of excess risk with attained age or age at exposure was assumed across cancer sites. Since the biological processes leading to cancer differ for different organs, organ-specific age dependencies might differ substantially. Therefore, each cancer site or group of cancer types was tested separately for significant age modifiers.
2. Generally, risk models only include statistically significant (at the 95% level) parameters. This provides a uniform criterion for selecting parameter values and ensures that baseline and risk parameters reflect the epidemiological evidence, thus it removes arbitrariness which parameters should be included, and which should be discarded. Furthermore, it avoids possible implausible extrapolations for ages at exposure or attained ages where cancer data are sparse. Finally, including non-significant parameters in the risk models may lead to large parameter uncertainties with possibly large parameter correlations in the covariance matrix. This can result in large uncertainties of the risk estimates, in particular outside the central region of the data.
3. Multi-model inference (MMI) has been applied to two different situations: all models with a similar quality of fit were included for the final risk estimate with a weighting according to the AIC criterion. This avoids bias by selection of one particular model. Furthermore, MMI was employed for some cancer sites with strong differences between sexes. This avoids potentially significant sex-specific underestimation of risk. Details are given below.

### Grouping of cancer sites

Incidence rates for the most frequent cancers do not exceed 1% per person-year (IARC 2014; GEKID 2012; RKI 2017, 2020). Correspondingly, epidemiological studies aiming to quantify the impact of radiation on site-specific cancer development are commonly challenged by low incidence rates of the diseases with relatively small numbers of cancer cases and, more importantly, small number of cases attributable to radiation. This leads to a situation in which reliable radiation risk models can only be developed for the most prevalent types of cancer, such as colon, stomach, female breast, lung and thyroid cancer. For less prevalent cancers, the observed numbers of cases are low, and the data do not allow derivation of reliable evidence-based radiation effect models. Therefore, aggregation of site-specific malignancies into groups of functionally similar diseases is necessary to obtain sufficient statistical power for developing robust risk models.

During the development ProZES, there were extensive discussions of how to group cancer sites for the low-LET models, with goal of being as specific as possible, while taking into account the potential and limitations of the LSS cohort data. Finally, an approach for grouping of cancer sites based on the following criteria was chosen:

1. Cancer sites should be functionally related, as discussed below.
2. Cancer sites should demonstrate a compatible behaviour of baseline incidence rates, i.e., a similar relative age dependency, although absolute values of the baseline rates can differ for different sites, populations, and time periods.
3. The number of cases in the epidemiological cohort should be sufficient for reliable statistical inference and robust radiation risk models. For solid cancers in the LSS, the groups should include about 300 or more cases. For hematopoietic cancers with their larger radiation-attributable fractions, the groups were allowed to be smaller, as described below in the model section.
4. ERR values for the individual cancer sites included in a cancer grouping should be consistent taking into account the error bounds.
5. The compatibility of the diseases in a group was checked by comparing the baseline cancer incidence data for various diagnoses as found in the contemporary population cancer registers in Japan (NCC 2013) and Germany (RKI 2017). This comparison included the investigation of calendar year effects and of relative age dependencies.

It was considered important that the cancers within a group would be functionally or physiologically related. For example, it would be difficult to justify risk estimates for female genital cancers based on a group that includes brain or skin cancer. The relative age dependence of the single cancer sites should be also compatible, otherwise the derived risk estimates and the dose and age dependencies of risk might be distorted. One notable example is female genital cancer. It was found that cervical cancer in Japan has very different age patterns compared to other female genital cancers, and therefore, female genital cancers were split into two separate groups (cervix and female genital organs, excluding cervix). Another example is cancer of the liver which, with its 1494 cases in the LSS, would qualify as a separate group according to these criteria. However, liver cancer is often related to other malignancies, so differentiation between primary and secondary malignancy is not straightforward (Preston et al. 2007). Therefore, it was decided to include liver cancer in the digestive group. A remainder group includes all types of cancers that either could not be reasonably assigned to a specific group (e.g. bone cancer), or that did not fulfil the above criteria (e.g. testis cancer).

For solid cancers, risk models were developed for cancers of the colon (COLON), stomach (STOMACH), female breast (BREAST), lung and trachea (LUNG), thyroid (THYROID), digestive tract without colon and stomach (DIG), urinary tract (URI), cervix (GNF1), female genital organs, excluding cervix (GNF2), male genital organs (GNM), brain and central nervous system (BCNS), non-melanoma skin (SKIN), and remaining organs (REM). Hematopoietic malignant diseases were split into four groups, thus representing acute lymphoblastic leukaemia (HEM1), lymphoma including chronic lymphoblastic leukaemia (HEM2), acute myeloid leukaemia (HEM3), and chronic myeloid leukaemia (HEM4). The full list of risk models together with the diagnoses and ICD10 codes can be found in Table 1.

**Table 1:** Grouping of malignant diseases for modelling in ProZES

Model name	Organ or organ group and diagnose (ICD10 code)
*Solid cancers*	
STOMACH	Stomach (C16)
COLON	Colon (C18)
LUNG	Lung and trachea (C33, C34)
BREAST	Female breast (C50)
THYROID	Thyroid (C73)
DIG	Oral cavity (C00–C14), esophagus (C15), small intestine (C17), rectum (C19–C21), liver (C22), gallbladder (C23, C24), pancreas (C25), other digestive (C26, C48)
URI	Kidneys (C64), renal pelvis and ureter (C65,C66), urinary bladder (C67), other urinary (C68)
GNF1	Uterus/cervix (C53)
GNF2	Uterus/corpus (C54) or uterus/non-specified (C55), ovaries (C56), other female genital organs (C51, C52, C57, C58)
GNM	Prostate (C61), other male genital (C60, C63)
BCNS	Eyes (C69), brain and CNS (C70–C72)
SKIN	Skin (non-melanoma cancer, C44)
REM	Nasal cavity (C30, C31), larynx (C32), thymus (C37), heart and intrathoracic (C38, C39), bone (C40, C41), connective tissue (C45–C47, C49), testis (C62), adrenals (C74), other endocrine (C75, C76)
*Hematopoietic malignant diseases*	
HEM1	Acute lymphoblastic leukaemia (ALL, C91.0), prolymphocytic leukaemia of B-cell type (C91.3), lymphoid leukaemia/unspecified (C91.9)
HEM2	Hodgkin lymphoma (C81), non-Hodgkin lymphoma (C82, C83, C85, C86), lymphoma of peripheral and cutaneous T-cell (C84), malignant immunoproliferative disease (C88), chronic lymphoblastic leukaemia (CLL, C91.1), hairy cell leukaemia (C91.4)
HEM3	Acute myeloid leukaemia (AML, C92.0), sub-acute myeloid leukaemia (C92.2), myeloid sarcoma (C92.3), acute promyelocytic leukaemia (C92.4), acute myelomonocytic leukaemia (C92.5), monocytic leukaemia (C93), other leukaemia of specified cell type (C94), leukaemia of unspecified cell type (C95), other or non-specified (C96)
HEM4	Chronic myeloid leukaemia (CML, C92.1)

Radiation-induced mechanisms of carcinogenesis in single cancer sites within a group can differ, and potentially lead to different risk estimates and age dependencies. However, the chosen methodology of grouping necessarily represents a compromise between model specificity on one side, and the practical epidemiological requirements of feasibility, plausibility and robustness of the radiation risk models on the other side.

### Application of the multi-model inference principle in ProZES

Development of the risk models in ProZES included a systematic use of multi-model inference (MMI). MMI was used with the following goals: to reduce the dependence on one selected model, to better reflect the inherent dose, age and sex dependencies present in the epidemiological data, and to provide a more realistic assessment of uncertainties. To achieve these goals, MMI was applied in two different situations.

First, for many organs, two or more models provided a comparable statistical quality of fit. These models were weighted based on differences in the Akaike information criterion (AIC). Often, both ERR and EAR models qualified providing a similar description of baseline but differing in the age dependency of the radiation risk. The final MMI-averaged model will then reflect an intermediate age dependency.

Second, for some cancer sites, very strong sex differences were observed, including colon cancer and lymphoma including chronic lymphocytic leukaemia. For example, for colon cancer, it was found that the ERR for males was relatively independent of attained age, whereas females had a strongly decreasing risk with increasing age. This difference was considered to be rather implausible on biological grounds. However, using only risk models with a joint age dependency would neglect the statistically significant sex differences observed in the epidemiological data. To account for both aspects, it was decided to give each approach the same weight. Thus, the risk of colon cancer is calculated using a 50% weight to models with sex-specific dependency on attained age and a 50% weight to models with joint attained age dependency. Furthermore, several models were selected and weighted according to AIC within each group.

The section “Description of site-specific risk models” and the supplementary materials provide details for each cancer grouping, including the information about the application of MMI in each case.

### Generic form of risk models

Fitting of radiation risk models, with a few exceptions, was performed using a common generic phenomenological model framework, described below.

The LSS cohort data are represented by categorical cells that contain information on the number of observed cancer cases and person-years obtained by stratification of the individual data depending on sex *s*, attained age *a*, age at exposure *e*, birth year *b*, dose *d*, and diagnosed cancer. The incidence rates were defined either as excess relative risk (ERR) or excess absolute rate (EAR) model:

10 $$\lambda = \lambda_{0} \left( {1 + {\text{ERR}}} \right) = \lambda_{0} + {\text{EAR}} .$$

Here, $\lambda_{0}$ is the baseline incidence rate in the absence of radiation, and $\lambda$ is the total incidence rate after exposure to radiation. Additionally, a screening factor may be present as in the case for thyroid cancer. Such a factor accounts for time-dependent cancer screening in the LSS cohort due to periodical medical examinations and the autopsy program, which was most actively run in the period before 1970 (Hayashi et al. 2010; Grant et al. 2017). The baseline rate fitted to the LSS data is represented as a product of two functions:

11 $$\lambda_{0} = f\left( {{\varvec{\upbeta}}|s,a,b} \right) g\left( {{\varvec{\upbeta}}|c,{\text{IC}}} \right),$$

where ${\varvec{\upbeta}}$ is the vector of fit parameters. The function $f$ describes the sex-, age- and birthyear-dependent baseline incidence rate. The function $g$ depends on cohort-specific parameters $\left( {c,{\text{IC}}} \right)$. The city parameter *c* indicates whether a person lived in Hiroshima or Nagasaki, and the “in-city” parameter IC indicates whether a person was in the city at the time of bombing. These parameters are meaningless for target populations beyond the LSS cohort. Consequently, if the fitted model baseline includes these LSS-specific parameters, they have to be averaged, using the number of cancer cases or person-years as averaging weights, and used as a factor modifying the cohort-independent model baseline $f$. The resulting model baseline rate is represented as a product of the rate $f\left( \cdot \right)$ with all cohort-independent explanatory variables and of the average modifying factor $g$ calculated using the number of cancer cases or, for a few models, person-years in groups of cohort members, stratified according to their residence and location at the time of detonation.

The ERR and EAR have been modelled using the following generic functional form:

12 $$\left. {\begin{array}{*{20}c} {\text{ERR}} \\ {\text{EAR}} \\ \end{array} } \right\} = p\left( {{\varvec{\uprho}} |d,s} \right) r\left( {{\varvec{\uprho}} |s,a,e} \right),$$

where ${\varvec{\uprho}}$ is the vector of risk-specific parameters. The function $p$ characterises sex-specific radiation effects of dose $d$ (linear, linear-quadratic, power, exponential), and the function $r$ includes risk modifiers for sex, age attained and age at exposure. The specific forms of the risk models are provided in the Supplement. The indicator variables were defined as:

13 $$\begin{aligned} &s = \left\{ {\begin{array}{*{20}c} { - 1} & {\text{male}} \\ { + 1} & {\text{female}} \\ \end{array} } \right.\;\,c = \left\{ {\begin{array}{*{20}c} { - 1} & {\text{Hiroshima}} \\ { + 1} & {\text{Nagasaki}} \\ \end{array} } \right.\\&{\text{IC}} = \left\{ {\begin{array}{*{20}c} 1 & {{\text{in}}\;{\text{city}}} \\ 0 & {{\text{not in}}\;{\text{city}}} \\ \end{array} } \right.. \end{aligned}$$

For every cancer grouping, all possible effect modifiers were tested, and generally only the statistically significant ones (at 95% confidence level) were preserved in the final models.

### Details of the fitting procedure

Most of the radiation risk models were obtained from a reanalysis of the LSS data. The reanalysis was considered necessary for ProZES, for the following reasons:

- Model baseline rates were needed in addition to the excess rates for assessment of the assigned share, and for risk transfer to the target population
- For most groupings of cancer sites, no risk models existed in the literature
- Both the parameter uncertainties and the full covariance matrices were necessary for the uncertainty assessment
- To ensure that the general principles of the ProZES methodology were followed, including use of model selection by multi-model inference.

Unless otherwise specified, the model parameters were determined by fitting the LSS cancer incidence data for the follow-up periods 1958–1998 for solid cancers and 1950–2001 for hematopoietic malignancies. The best fit parameters were obtained from Poisson regression by minimization of the deviance, ${\text{dev}} = - 2\mathop \sum \limits_{i} l_{i}$, where $l_{i}$ is the likelihood of the *i*th cell, and the sum runs over all cells *i* in the LSS dataset, which forms a matrix constructed from vectors specifying the cells:

14 $${\mathbf{X}} = \left( {{\mathbf{s}},{\mathbf{a}},{\mathbf{e}},{\mathbf{d}},{\mathbf{c}},{\mathbf{IC}},{\mathbf{n}},{\mathbf{PY}}} \right),$$

where ${\mathbf{n}}$ and ${\mathbf{PY}}$ are the vectors of the observed number of cancer cases and person years. The deviance was then computed as

15 $$\begin{aligned} {\text{dev}}\left( {{\varvec{\upbeta}},{\mathbf{X}}} \right) &= 2\mathop \sum \limits_{{i:n_{i} \ne 0}} \left[ {n_{i} \ln \frac{{n_{i} }}{{\mu_{i} \left( {{\varvec{\upbeta}}, {\mathbf{X}}_{i} } \right)}} - \left( {n_{i} - \mu_{i} \left( {{\varvec{\upbeta}}, {\mathbf{X}}_{i} } \right)} \right)} \right]\\&\quad + 2\mathop \sum \limits_{{i:n_{i} = 0}} \mu_{i} \left( {{\varvec{\upbeta}},{\mathbf{X}}_{i} } \right), \end{aligned}$$

where

16 $$\mu_{i} \left( {{\varvec{\upbeta}}, {\mathbf{X}}_{i} } \right) = \lambda \left( {{\varvec{\upbeta}},{\mathbf{X}}_{i} } \right)PY_{i}$$

is the model-estimated expected number of cases for the *i*th cell with ${\mathbf{X}}_{i} = \left( {s_{i} , a_{i} , e_{i} , d_{i} , c_{i} , IC_{i} , n_{i} , PY_{i} } \right)$. Unconstrained quasi-Newton minimization as implemented in Matlab’s Optimization Toolbox^3^ was used to minimize the deviance. Minimisation results were evaluated using AIC and likelihood ratio test (LRT). The final fitted parameters were independently checked using the EPICURE software tool (Preston et al. 2015) as a part of quality assurance procedures.

If not described otherwise, only statistically significant model parameters ($p \le 0.05$) were kept for the final models. Exceptions were made only for parameters of radiation-attributed excess rate if the epidemiological evidence did not allow for statistically significant estimates and the 95% confidence interval included zero risk, also. In such cases, the relatively large uncertainties of the model parameters are reflected in the uncertainty distribution of the assigned share.

The fitted models for baseline rates in the LSS were checked for plausibility by comparing these rates with cancer incidence rates observed in the entire Japan for various years (NCC 2013), and with incidence rates reported for Hiroshima and Nagasaki cities (IARC 2014). Generally, the model baselines were in good agreement with the incidence rates obtained from the population registries for different time periods. Details and examples can be found in Appendix 2 of the report of Ulanowski et al. (2016).

To account for the cohort-specific uncertainty in dosimetry for members of the LSS cohort, a multiplicative factor described by a lognormal probability density function centred at 1.0 with a geometric standard deviation GSD = 1.1 was applied based on expert judgement. Additional cohort-specific uncertainty accounted for the uncertainty associated with the neutron radiation weighting factor used to calculate the weighted organ dose for the LSS cohort members. The uncertainty in the neutron weighting factor was simulated by a triangle distribution between 5 and 30 with the mode 10 and using the estimated risk response to varying values of the neutron weighting factor for internal and external organs, separately (see details in Jacob et al. 2013).

## Description of site-specific models

This section summarizes the main features of the risk models used in ProZES. Details of each model including formulas for baseline and excess risks, estimated parameter values and covariance matrices are provided in the Supplement. Additional information, such as plots of ERR versus attained age, or dependence of the assigned share *Z* on different exposure scenarios, can be found in the ProZES reports (Jacob et al. 2013; Ulanowski et al. 2016). The properties of several grouped models are summarized in Table 2.

**Table 2:** Main parameters of the models derived for several groupings of solid cancers based on the LSS data

Group	Cases	ERR (Gy^−1^)			EAR (10^−4^ PY^−1^ Gy^−1^)		
		Attrib. fraction^a^ (%)	Constant (*p* value)	Power of att. age^b^ (*p* value)	Attrib. fraction^a^ (%)	Constant (*p* value)	Power of att. age^b^ (*p* value)
DIG	4083	2.8	0.24 (0.001)	−3.04 (< 0.001)	2.4	6.85 (< 0.001)	2.26 (< 0.001)
URI	741	7.9	1.21^c^ (< 0.001)	–	7.3	4.19 (< 0.001)	3.63 (< 0.001)
GNF1	978	0.45	0.06 (0.68)	–	0.8	0.57 (0.4)	–
GNF2	479	2.7	0.35 (0.12)	–	1.5	0.49 (0.3)	–
GNM	403	1.1	0.12 (0.56)	–	1.9	1.39 (0.38)	2.7 (0.3)
		1.6	0.20 (0.37)	−3.7 (0.3)			
BCNS	281	5.0	0.23 (0.23)	−2.97 (0.009)	4.1	0.46 (0.046)	–
SKIN	330	11.7	0.71^d,e^ (0.018)	–	11.2	1.1^f,g^ (0.021)	3.65 (< 0.001)
REM	324	4.8	0.25 (0.20)	−2.77 (0.02)	4.5	0.60 ^h^ (0.03)	–

^a^Fraction of the observed incidence rate, which is attributed to radiation exposure

^b^Centred at attained age 70

^c^Sex-averaged value; effect of sex is significant (*p* = 0.011) and results at age 70 in sex-dependent ERR per 1 Gy ≈ 0.5 (males) and ≈ 1.9 (females)

^d^Non-linear dose response with dose exponent equal to 1.55 (*p *< 0.001)

^e^‘Age-at exposure’ effect modifier of log-risk equals to –89% per decade (*p* < 0.001)

^f^Non-linear dose response with dose exponent 1.60 (*p *< 0.001)

^g^‘Age-at-exposure’ effect modifier of log risk equals to −75% per decade (*p* < 0.001)

^h^Sex-averaged value; effect of sex has low significance (*p* = 0.14) resulting in sex-dependent EAR (10^−4^ PY^−1^ Gy^−1^) of 1.0 (males) and 0.2 (females)

### Models for solid cancers after low-LET exposure

#### Stomach cancer (STOMACH, ICD10:C16)

Four stomach cancer risk models were identified by fitting the LSS data for the follow-up period 1958–1998. The models, two of ERR-type and two of EAR-type, were aggregated using multi-model inference (MMI) based on their AIC weights. All models have a linear dose response. Risk coefficients in the EAR models are the same for the both sexes, while in the ERR models the risk is sex dependent. Three models (two of EAR- and one of ERR-type) with the total AIC weight exceeding 95% include risk modifiers depending on attained age, while the fourth is an ERR-type model and includes age-at-exposure as a risk modifier.

**Fig. 3:**
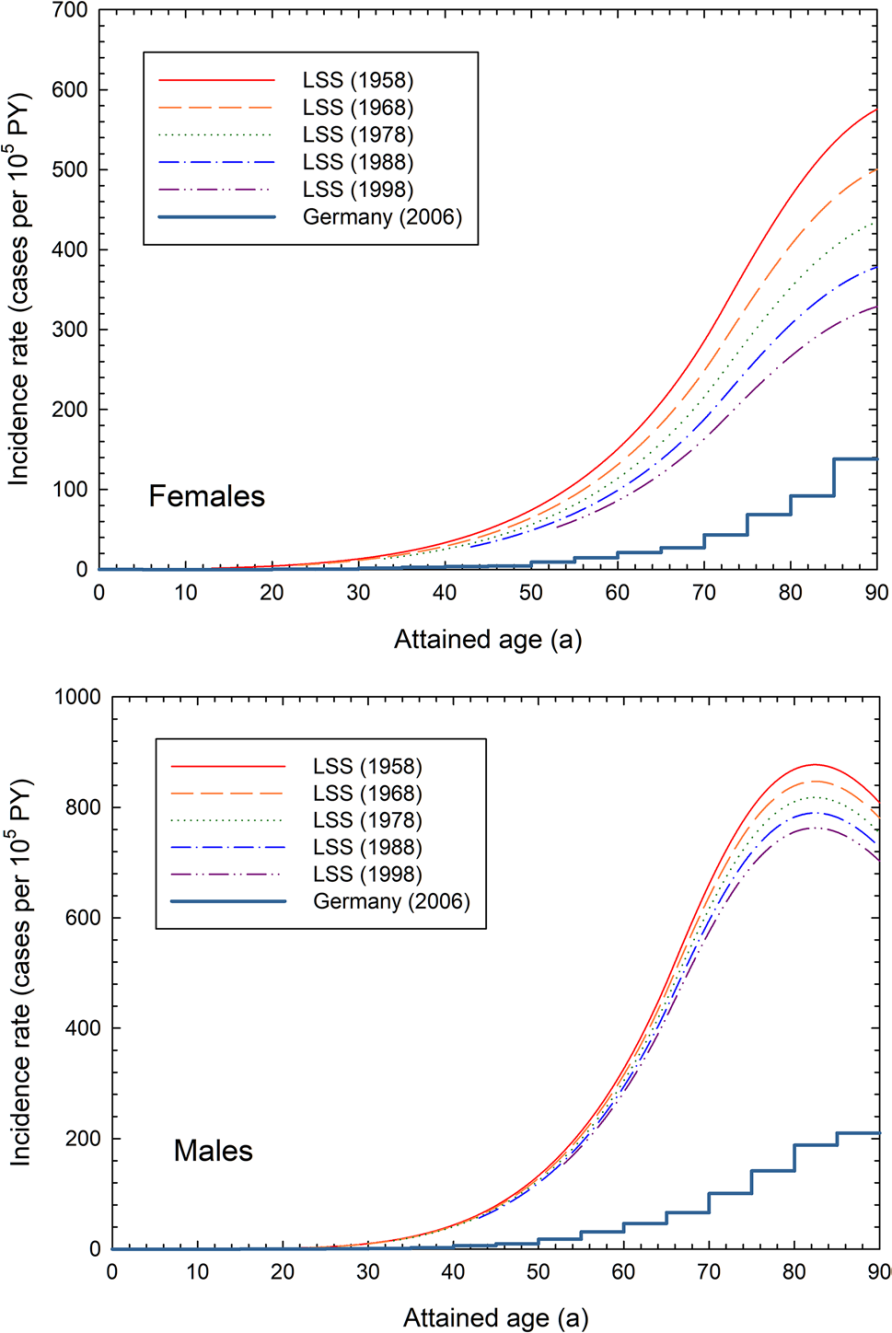
Fitted baseline incidence rates of stomach cancer in the LSS cohort for different calendar years compared to the stomach cancer incidence rate in Germany in 2006 for females (top) and males (bottom)

Figure 3 contrasts the fitted age-specific spontaneous rates seen in the Japanese LSS population with the much lower rates observed in the German population. The figure also indicates how age-specific rates in the LSS have declined over time. Because of the marked difference in Japanese and German stomach cancer rates, additive and multiplicative risk transfer from the LSS cohort to the German population leads to markedly different risk estimates. Therefore, as explained in the methodology section, ProZES systematically uses a risk transfer which involves both transfer pathways combined with a uniformly distributed random transfer factor. This procedure adds additional uncertainty to the estimated values of Z. The larger the differences between the baseline rates in the two populations the greater the uncertainty introduced by the transfer of risk.

#### Colon cancer (COLON, ICD10:C18)

For colon cancer in the LSS, the dependence of ERR on attained age differs strongly between males and females. Whereas for males, the dependence of ERR on attained age is relatively flat, the fitted ERR for females decreases markedly with increasing age. However, no biological mechanism is known that supports such a sex difference. Furthermore, the baseline rates of males and females show similar age dependencies. To account for both possibilities, a decision was made to assign an equal MMI weight of 50% to sets of models with and without sex-dependent attained age effect modification of radiation risk. With this approach, the final ProZES model for radiation risk reflects both the existing sex differences suggested by the epidemiological data and biological plausibility. The increased error bounds for *Z* from the MMI procedure include this additional source of uncertainty.

For each set of models, the usual model selection procedure was performed, including likelihood ratio tests and MMI weighting based on AIC. There were four sex-specific attained-age-dependent models, two for males and two for females, and three sex-independent models (see Fig. S2.1). For each sex, the final model was constructed from the two sex-specific models and the three common models. For risk transfer to the German population, it was important to account for the calendar period. Colon cancer incidence rates in the LSS increase strongly with calendar year. The incidence rates of later calendar years become more compatible to those for the contemporary German population.

#### Cancer of lung and trachea (LUNG, ICD10:C33, C34)

For lung cancer, the model of Furukawa et al. (2010) was selected for use in ProZES. It is based on the LSS data set with additional information on influence of smoking on radiation risk. Among the four selected models, the simple additive (AM) and simple multiplicative (MM) models describe independent effects of smoking and radiation on lung cancer incidence. The more complex generalized additive (GAM) and generalized multiplicative (GMM) models include explicit interaction terms between radiation and smoking. The generalized models provide a significantly better fit to the data, where the ERR depends on smoking intensity in a non-linear way: ERR increases with increasing smoking intensity up to about 5–10 cigarettes per day (cpd), then it decreases strongly and almost vanishes for more than 20–25 cpd.

**Fig. 4:**
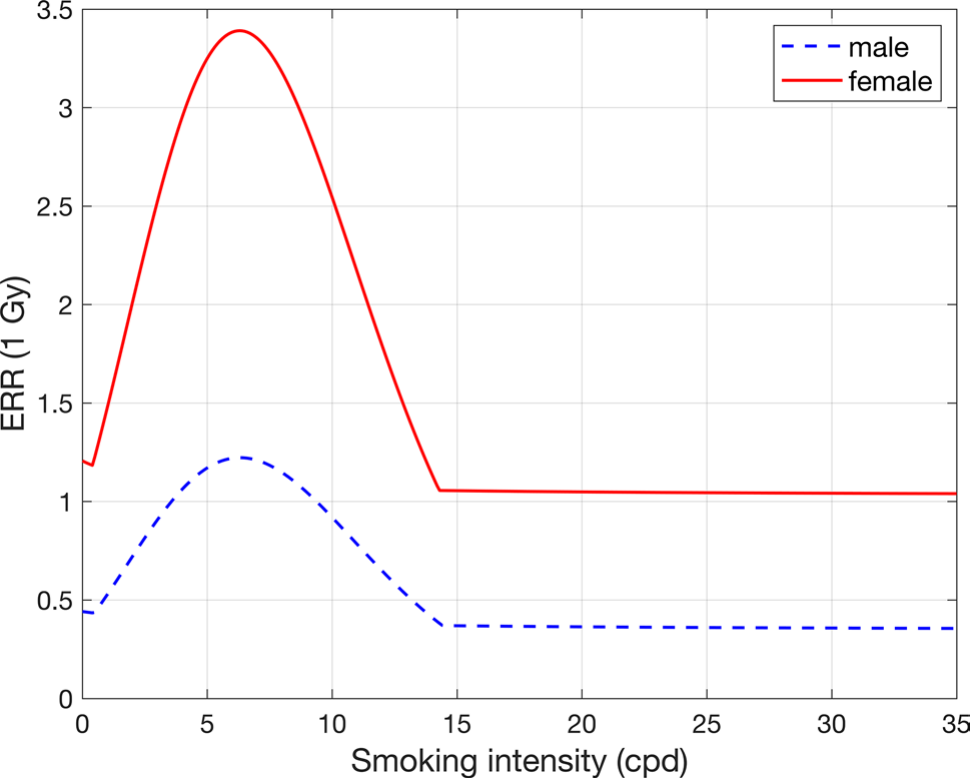
Radiation ERR at 1 Gy of the lung cancer model selected for ProZES as a function of smoking intensity for females (solid red) and males (dashed blue) for age at exposure of 30 years and attained age of 70 years. Smoking started at age 20 until age 70 (colour figure online)

While the vanishing ERR might reflect the large influence of heavy smoking on the spontaneous lung cancer incidence, it is also accompanied by higher uncertainties; therefore, it is questionable if such a strong decrease in relative risk is plausible and can be justified. Furthermore, the radiation risk values at high smoking intensities depend on the assumed model function. Therefore, due to increasing uncertainty of radiation risk at high smoking intensities and trying to avoid potential underestimation of radiation risk, it was decided not to rely on the generalized models alone, but conservatively use the maximum ERR value of either the simple or the generalized models. Effectively, this leads to a constant ERR for large smoking intensities, see Fig. 4.

Although, for calculation of radiation risk of lung cancer, the ProZES models require information on smoking, provision of personal information on smoking behaviour is not mandatory. By selection of “unknown” smoking status, the software applies random sampling for smoking status and related parameters using the distributions describing smoking prevalence and habits in Germany (Lampert 2011, Jacob et al. 2013).

By definition, the lung cancer baseline rates in the risk model of Furukawa et al. (2010) represent spontaneous disease rates for non-exposed never-smokers. Unfortunately, such statistical data are not readily available for the German population. Therefore, the transfer factor from the LSS cohort to the German population is modelled stochastically, assuming that the ratio of baselines *B* (see Eq. 3) is log-uniformly distributed in a range from 1/3 to 3.

#### Female breast cancer (BREAST, ICD10:C50)

The breast cancer model from the pooled study of Preston et al. (2002) was used for ProZES, since it includes not only the LSS data, but also results from several other studies of radiation-induced breast cancer in Western populations. The risk model is an EAR-type model with explicit dependence on attained age and age at exposure. In the pooled study, no age-dependent baseline rates are available. Therefore, the transfer factor to the German population is modelled stochastically with the assumption that the ratio of baselines is log-uniformly distributed in range from 1/3 to 3, similar as it is done for lung cancer. This method of risk transfer modelling additionally increases the uncertainty range of the estimated assigned share. The breast and lung cancer models are the only low-LET exposure risk models that were not fitted explicitly for ProZES. For breast cancer, no data on the other breast cancer studies included in the pooled study were available to the authors, and in case of lung cancer in the LSS additional information on smoking was missing in the available dataset. Male breast cancer is rare and is not considered in ProZES.

#### Thyroid cancer (THYROID, ICD10:C73)

The thyroid cancer model (Jacob et al. 2014), applied in ProZES, is based on an analysis of the LSS data with explicit modelling of a screening effect created by the additional medical surveillance received by the LSS cohort members participating in the Adult Health Study (AHS) (Wong et al. 1993) and the autopsy studies for deceased LSS cohort members (Hayashi et al. 2010). The screening effect resulted in higher thyroid cancer incidence rates in the cohort. The screening factor was found to be significantly different for time periods before 1970 and after, thus likely reflecting the changing screening or autopsy practices for the LSS and the AHS members. The risk model used is an ERR model with explicit dependence on attained age and age at exposure. The relative risk decreases with increasing attained age and increasing age at exposure.

#### Cancer of digestive organs, excluding stomach and colon (DIG, ICD10:C00-C15, C17, C19-C26, C48)

The group DIG combines cancers of the digestive tract organs other than colon and stomach. Baseline incidence rates of these cancers are generally lower than that of colon and stomach, so there are fewer cases in the LSS cohort diagnosed with these diseases and no statistically significant radiation effect can be identified for a specific disease within this group. Plausibility of the grouping was justified by checking for compatibility of their relative baseline rates (see Appendix 2 of the ProZES report, Ulanowski et al. 2016). This assures that the same phenomenological model can be applied to describe the cancer risk for the selected diagnoses.

Fitting of these grouped data resulted in two models, one of ERR-type and one of EAR-type, that were included in ProZES with MMI weights based on their AIC values. The ERR-type model received weight of 71% while the EAR-type model’s weight was 29%. Both models were linear in dose. The relative risk was found to depend explicitly on attained age and resulted in a decreasing ERR, and correspondingly decreasing assigned share *Z*, with increasing attained age.

#### Cancer of urinary organs (URI, ICD10:C64–C68)

The group URI combines cancers of urinary system organs. The plausibility of this grouping was established based on a compatibility check similar to that used for the group DIG. The resulting models of almost equal AIC weights were an EAR-type and an ERR-type model with weights of 53% and 47%, respectively. Both models were linear in dose. The ERR-type model was independent of any age modifiers; the EAR-type model depended on attained age. The resulting MMI average for the assigned share showed a moderately decreasing *Z* with increasing attained age for most exposures.

#### Cervical cancer (GNF1, ICD10:C53)

A combination of all female genital organ cancers into one group resulted in an inconsistent data set, because an analysis of baseline incidence rates in Japan revealed that the age dependence of spontaneous cervical cancer differs substantially from that for other parts of the uterus, ovaries and other genital organs. The cervical cancer baseline incidence rate in Japan in 2010 shows a peak at age 40 and decreases at older ages, whereas such age patterns are not observed for other genital cancers. Since such incompatible time dependence might lead to bias in the shape of dose response and the age dependence of risk and the assigned share, it was decided to split cancers of female genital organs into two groups with a separate group to represent cancer of cervix uteri.

Two models for cervical cancer were selected for MMI, an EAR-type and an ERR-type model with 57% and 43% AIC weights, respectively. Both models are linear in dose without any effect modifiers. The risk estimates are quite low and not statistically significant, resulting in a low assigned share. The cervical cancer baseline in Japan, including Hiroshima and Nagasaki cities, is strongly affected by screening and prevention (vaccination) actions taken during the last decades (Tsuji 2009; Konno et al. 2010). These result in non-standard age dependencies of the baseline, including strong non-linear time trends. Further modelling dedicated for cervical cancer might improve the baseline and risk description.

#### Cancer of female genital organs other than cervix (GNF2, ICD10:C51–C52, C54–C58)

For the group GNF2, two models, an ERR-type and an EAR-type, were selected with 72% and 28% AIC weights, respectively. The statistical significance was not sufficient to identify age dependencies of radiation risk, thus both models are linear in dose without any effect modifiers.

#### Cancer of male genital organs (GNM, ICD10:C60-C61, C63)

The group GNM includes cancers of male genital organs, of which cancer of the prostate accounts for 387 of 403 cases. Originally attributed to the GNM group, 17 cases of testicular cancer were re-assigned to the group of remaining cancers (REM) because of the distinctively different age dependency of the testicular cancer incidence rate. Unlike prostate cancer, the incidence rate of testicular cancer starts to increase after age 15, approaches a maximum between 30 and 35, and falls to minimal levels after age 50–55.

Three final models were identified, two models of ERR-type and one EAR-type model. The ERR model with the largest AIC weight of 76% is a linear dose response model without effect modifiers. The remaining ERR model and the EAR model with 19% and 5% weight, respectively, depend explicitly on attained age. The risk estimates have low statistical significance. The magnitude of risk is relatively low, with assigned shares expected to be lower compared to other cancer sites for the same dose.

#### Cancer of brain and central nervous system (BCNS, ICD10: C69–C72)

The group of brain and central nervous system consists of one ERR-type model with 58% AIC weight, and one EAR-type model with 42% weight. Both models are linear in dose. The EAR-type model does not include any effect modifiers, whereas the ERR-type model depends on attained age. The relative risk and, correspondingly, the assigned share decrease with increasing attained age.

#### Non-melanoma skin cancer (SKIN, ICD10: C44)

The non-melanoma skin cancer group includes one ERR-type and one EAR-type model with 72% and 28% AIC weights, respectively. However, the dose response is different from most other solid cancers. The models show a dose response that is significantly non-linear with a power form: in both models, the risk increases with dose to a power of about 1.5–1.6. Therefore, the risk is very small at low doses but increases more strongly at high doses. Furthermore, both models depend on age at exposure, and risk decreases for older age at exposure. In addition, the EAR-type model depends on attained age.

The non-linear power dose response of the models may result in lower values of radiation risk and assigned share following fractionated exposures, than the values of risk and assigned share estimated for a single exposure with the same dose. Currently, available epidemiological data for the LSS do not suggest alternative shapes of dose responses due to missing evidence of radiation risk at doses less than 1 Gy. In case of fractionated low-dose exposures, it could be advised to calculate additionally, as a conservative upper limit, the assigned share using a single exposure with dose equal to the total dose of fractionated exposures.

ProZES does not contain a model for melanoma skin cancer. It is a rare cancer in the LSS cohort with only 17 reported cases, and no significant estimates of radiation-associated risk can be safely inferred. Additionally, it is unclear if the radiation mechanisms for induction of melanoma are similar to other solid cancers. Furthermore, UV light might have a strong confounding effect.

#### Cancer of the remaining organs (REM, ICD10: C30–C32, C37–C41, C45–C47, C49, C62, C74–C76)

The group of “remaining” solid cancers includes cancers of the nasal cavity, larynx, thymus, heart and intrathoracic, bone, connective tissue, testis, adrenals, and other endocrine organs. The REM group combines all solid cancer types in the LSS which could not be well attributed to the other groups, and for which the number of observed cancer cases was not sufficient for inference of radiation risk. It also includes 17 testicular cancer cases, because the age dependence of the baseline incidence rate of this disease was incompatible to other male genital cancers, in particular, of prostate cancer.

The REM group contains an ERR-type and an EAR-type model with 67% and 33% AIC weights, respectively. The dose response of the both models is linear. The EAR-type model is independent of attained age, whereas the ERR-type model depends on attained age. The relative risk and the assigned share decrease with increasing attained age.

### Models for hematopoietic malignant diseases after low-LET exposure

The models for radiation risk of leukaemia are based on the LSS incidence data from 1950 to 2001. In total, 944 leukaemia cases were observed. The present work uses similar grouping of leukaemia subtypes as suggested by Hsu et al. (2013) where detailed descriptions on the background of the data set and the grouping can be found. Four leukaemia models are implemented in ProZES:

- Acute lymphoblastic leukaemia (ALL), 43 cases
- Lymphoma including chronic lymphoblastic leukaemia (CLL), 449 cases
- Acute myeloid leukaemia (AML), 176 cases
- Chronic myeloid leukaemia (CML), 75 cases

The structure of the risk models is similar to Hsu et al., however, the models were newly developed using the ProZES methodology including multi-model inference. In addition, for risk prediction and potential use in compensation claims so-called ‘twin’ models were introduced, as explained below. Model fits were performed using doses for red bone marrow.

Currently, no model for multiple myeloma (136 cases) is implemented, but is anticipated to be added in future updates of ProZES. Adult T-cell leukaemia is known to be caused by infection of viral origin (Takatsuki 2005), thus the 47 cases of this disease were excluded from the radiation risk modelling. Eighteen (18) cases of other leukaemia types were also not included in any disease group for the analysis. CLL (10 cases) was grouped together with various lymphomas.

The leukaemia risk models are among the most challenging models for risk assessment and compensation claims, for reasons described below:

- Due to the relatively small number of cases and the relatively high fraction (30–50%) attributed to radiation, a clear separation between baseline and radiation-induced cases is more difficult than for solid cancers
- Baseline and radiation risk often depend strongly on sex
- Radiation risk models show strong temporal effects depending on attained age, age at exposure and time since exposure
- Non-linear dose responses are found for several groups and for all leukaemia combined, mainly driven by the strong non-linear dose response of AML
- The period of latency for development for hematopoietic malignant diseases is typically shorter than that for solid cancers

The non-linear dose responses may lead to problematic risk estimates for fractionated and protracted exposures, which are typical exposure types in the case of compensation claims. Risk estimates are usually driven by epidemiological data from the medium-to-high dose range. Straightforward extrapolation of non-linear dose responses to low doses can result in implausible risk predictions. For example, let us assume a pure quadratic dose response like the one obtained for the AML model with the highest AIC weight. Fractionated exposure of 10 × 100 mGy reduces the risk by a factor of 10 compared to the risk estimate for a single exposure of 1 Gy, while a fractionation of 100 × 10 mGy results in risk reduction by a factor of 100 with the same total dose of 1 Gy. Protracted exposure can be seen as exposure of a large number of small doses and, as the number of fractions increases, the risk estimate for the protracted exposure tends to vanish for any value of total dose.

To avoid such wrong extrapolations, the method of model “twins” was introduced for the leukaemia models in ProZES. For any model with a non-linear dose response in the low-dose region, an additional “twin” model was added that shares the same form of baseline and effect modifiers but has a linear low-dose response. Following the standard MMI procedure adopted in ProZES, a model is randomly selected from the list of eligible models with probability defined by its AIC weight. If, however, the selected leukaemia model has the non-linear dose response, then the assigned share is computed both for the selected non-linear model and its linear “twin”. Then, the maximum of these two assigned share values is used, only. Since all non-linear leukaemia models have positive curvature at low doses, i.e. positive second derivative, this method forces selection of the linear “twin” model at sufficiently low doses and guarantees unambiguous risk estimates for fractionated and protracted exposures. For sufficiently high doses this procedure results in the usual MMI involving typically all models.

#### Acute lymphoblastic leukaemia (ALL), prolymphocytic leukaemia of B-cell type, lymphoid leukaemia/unspecified (HEM1, ICD10:C91.0, C91.3, C91.9)

The group HEM1, including ALL, has only 43 cases, but a high fraction of about 50% radiation-induced cases. Thus, it is difficult to provide a reliable description of the baseline, nevertheless, the sex-independent baseline was found to increase with age. Two EAR-type models were selected for MMI, one model linear in dose with 90% AIC weight, and one model quadratic in dose with 10% weight. The linear model serves as a twin for the quadratic one. Both models depend on attained age and sex. The radiation risk, namely, EAR and the corresponding ERR, decreases strongly with increasing attained age.

#### Hodgkin lymphoma, non-Hodgkin lymphoma, chronic lymphoblastic leukaemia (CLL), lymphoma of peripheral and cutaneous T-cell, malignant immunoproliferative disease, hairy cell leukaemia (HEM2, ICD10:C81–C86, C88, C91.1, C91.4)

The lymphoma group HEM2 is dominated by non-Hodgkin lymphoma with 402 of total 449 cases. Cases of Hodgkin lymphoma form the second largest group with 35 cases. While there is controversy in the literature whether CLL is induced by radiation, recent studies find an association between exposure and the disease (Hsu et al. 2013; Zablotska et al. 2013; Ojha et al. 2018), and for ProZES it was decided to include CLL in the HEM2 group. CLL is a very rare disease in Japan, and only 10 cases were observed in the LSS.

A separate fit for males and females resulted in no evidence of radiation risk for females, while for males, the fitted risk estimates were positive and statistically significant. Fitting both sexes together also resulted in significant positive risk. On biological grounds, it is very unlikely that males would be at risk for radiation-induced lymphoma, but females would have zero risk.

Therefore, the following decision was made for implementation of the HEM2 model in ProZES: both groups of sex-independent and of male-specific models are assigned an equal weight of 50%. That is, the MMI-selection procedure starts from a random selection of an appropriate sub-group of models. Then, within each of the two model sub-groups an AIC-based weighting is performed, resulting in one ERR-type and one EAR-type model for the joint set of models, and one ERR-type and one EAR-type model for the male-only model set. All models are linear in dose without effect modifiers, and there are no twin models. Both males and females are then evaluated with the same set of radiation risk models but with sex-specific baselines. This procedure ensures a plausibly conservative upper estimate of radiation risk for females. For males, the risk is between the sex-average and male-only risk and therefore somewhat lower than for the male-only risk model.

#### Acute myeloid leukaemia (AML), sub-acute myeloid leukaemia, myeloid sarcoma, acute promyelocytic leukaemia, acute myelomonocytic leukaemia, monocytic leukaemia, other leukaemia of specified cell type, leukaemia of unspecified cell type, other or non-specified (HEM3, ICD10:C92.0, C92.2, C92.3, C92.4, C92.5, C93–C96)

A characteristic feature of the HEM3 group is the strongly non-linear dose response. All models show a low radiation risk per dose at lower doses and a higher radiation risk at higher doses. The two models with highest AIC weight, one of an ERR-type and one of an EAR-type, have a pure quadratic dose response. These models would lead to vanishing risk and assigned share for protracted or highly fractionated exposures. Therefore, they were allowed to form twin pairs with threshold-linear-spline (TLS) dose response models. In the TLS models, risk increases with a smaller slope until about 0.7 Gy and, after that, risk increases stronger for higher doses. The ERR-type models depend on sex and age at exposure, whereas the EAR models are sex-independent and depend on attained age.

#### Chronic myeloid leukaemia (CML) (HEM4, ICD10:C92.1)

The group HEM4 of CML includes six models, four of an ERR-type and two of an EAR-type. CML shows strong temporal effects, and the radiation risk depends on time since exposure, attained age and age at exposure. Although the dose response is dominantly linear (91% of AIC weight for the linear models), two of the ERR-type models have a quadratic-exponential dose response. These models have “twins”, which are the corresponding ERR-type models linear in dose. Three of the models depend on time since exposure and attained age, and three models on age at exposure and attained age. Relative risk decreases strongly with increasing time since exposure. The ERR-type radiation risk models are sex-independent, whereas the EAR-type radiation risk models depend on sex.

### Models for lung cancer after high-LET exposure (Radon)

Lung cancer is the main health concern after exposure to radon and its progeny. For ProZES, two population target groups with radon exposure were identified: (a) former miners and occupational underground employees and (b) persons with occupational indoor exposure. It was, therefore, decided to implement two separate risk models for the two target groups. For miners, the model is derived from a study of the German Wismut miner cohort (Kreuzer et al. 2015), whereas the model for indoor exposure to radon is based on a pooled analysis of 13 European studies of residential exposure to radon in homes (Darby et al. 2005).

#### Miners

Studies of lung cancer among miners clearly show an increased risk of lung cancer associated with radon exposure (NRC 1999). Risk depends in a complex way on attained age, age at exposure and time since exposure. In addition, an inverse exposure rate effect is observed, where risk increases with decreasing exposure rate for the same total exposure. For ProZES, it was decided to base the risk model on the cohort of German uranium miners with low exposures and exposure rates. The Wismut cohort is the worldwide largest epidemiological cohort of miners exposed to radon. Furthermore, the cohort is relevant for compensation claims after radon exposure in mines in Germany. Patterns of low exposure and exposure rates better reflect current occupational exposure scenarios. Currently, the implemented model is based on a sub-cohort of Wismut employees hired in 1960 or later (Kreuzer et al. 2015). This model is preliminary, since it is a simple linear exposure response model of ERR-type without any effect modifiers. Exposure is specified in terms of working level month (WLM) (ICRP 2010). The miner model for radon exposure is anticipated to be revised soon based on upcoming national and international miner studies which are capable to provide additional evidence to quantify risk modifying effects by time, since exposure and age at exposure also at low radon exposures and exposure rates.

#### Indoor

For lung cancer after indoor radon exposure, the study of Darby et al. (2005) was selected. It is a large pooled analysis using data from 13 European case–control studies of lung cancer after residential radon exposure. Radon exposure was estimated by a time-weighted average of radon concentrations over the past 5–34 years before the diagnosis of (or death from) lung cancer, assuming a time lag of 5 years between exposure and cancer. This ERR-type model depends linearly on total exposure. Risk was stratified by smoking, age, sex, study and region of residence. There was little evidence that relative risk differed by smoking status or age. For males, the relative risk was higher than for females, but not significantly different (*p* = 0.19 for heterogeneity), therefore, only the joint risk estimate is used for ProZES.

For the indoor model, the total radon exposure is quantified by the average indoor air activity concentration (Bq/m^3^) times the duration of exposure. Therefore, the duration is a necessary input to quantify total exposure, and risk depends linearly on exposure duration. This contrasts with other ProZES models where exposure duration is only used for a potential low dose rate correction factor DREF.

## Discussion

### Considerations regarding the use of ProZES in compensation claims

Organisation of radiation protection at workplaces generally follows basic internationally adopted principles (ICRP 2007; IAEA 2014), which presume limiting radiation exposures to exclude (early) deterministic detrimental effects of radiation and to reduce as far as reasonably achievable the probability of (late) harmful stochastic effects.

Principles and implementations of compensation systems vary among countries. A review and comparison of various national compensation systems can be found elsewhere (ILO 2010). ProZES has been designed for use in Germany, where a decision on eligibility for compensation (SGB VII) is based on an assessment whether the observed disease is caused with predominant probability by occupational and insured activities that led to radiation exposure. In the field of compensation, predominant probability means that—under consideration of all circumstances—more reasons for causing the observed disease are related to the occupational field than to other exposure circumstances like naturally occurring radiation exposure or e.g. uninsured occupational exposure or private behaviour like smoking in the case of lung cancer.

ProZES was developed to support experts in the evaluation of this probability. The assigned share *Z* depends on the type of cancer, age at cancer occurrence, exposure history, and person-specific information such as sex and birth year. Furthermore, smoking information can be taken into account in the evaluation of *Z* for lung cancer, if available.

ProZES calculates an uncertainty distribution of the assigned share, from which all required statistics can be derived, including the mean, the median, and all percentiles. In the current version of ProZES, the median of *Z* is of particular relevance, because it provides a value bordering two equiprobable domains. If the median of *Z* exceeds 0.5, then the cancer can be regarded as more likely to be induced by occupational radiation exposures, and the person would be eligible for compensation. However, the expected value (the arithmetic mean) may be considered as an alternative quantity to facilitate advisory and decision making in compensation claims. During the development of ProZES, a substantial effort has been made to carefully assess various sources of uncertainty. Correspondingly, a combination of point estimates of the assigned share *Z* and confidence intervals, expressing uncertainties of these point values, provides essential information for making an informed decision on compensation claims, which should be based not only on point estimates but, additionally, involve consideration of the related uncertainties.

### Application to medical exposures

While the developed radiation risk models in ProZES can be used for compensation claims from occupational exposures, these models also have relevance for a much larger range of exposure situations. For example, medical applications of radiation may result in exposures that are often highly organ specific, so dedicated models of radiation risks are necessary to assess accompanying long-term risks. Diagnostic procedures may lead to exposures in the low- and medium-dose range, which is covered by the ProZES radiation risk models. Data exist suggesting that the risk estimates after diagnostic exposures to low-energy photons may require further adjustments for increased carcinogenic effectiveness; however, the topic is still under discussion and no adopted recommendations are available. Radiotherapy (RT) delivers high doses to a planned treatment target volume and to adjacent organs, while also creating lower dose exposures in other, more distant, organs. In addition, the dose distribution in the organs close to the treatment volume exhibits strong gradients, so a significant part of these organs is usually exposed in the medium dose range. While a number of studies on second primary cancer risk of RT patients estimate the excess relative risk for a therapeutic dose range above about 4 Gy, these studies have little statistical power at lower doses where the LSS-derived models are valid. With the aim to provide organ-specific risk models that cover a full dose range from low doses up to therapeutic doses, the ProZES risk models were combined with risk estimates from RT studies; then these models were integrated into the PASSOS software tool to estimate personalised lifetime risk of secondary cancer and heart diseases after breast RT (Eidemüller et al. 2019).

### Assigned share and probability of association

The definition of the assigned share *Z* of Eq. (1) has important consequences. Radiation risk estimates are obtained from an exposed population group. As the name indicates, the expected share of radiation in total risk of the population group is then assigned to a specific individual, adjusted for exposure characteristics, population, age, and other factors. However, from the perspective of the exposed individual who developed cancer, the situation is fundamentally different. It might be that radiation not only induced the cancer, measured by *Z*, but radiation could also have accelerated cancer development, leading to earlier manifestation of the disease and to lifetime lost. For the particular person who developed cancer at a specific age, the probability that her/his cancer is associated with radiation can therefore be larger than the assigned share.

Interestingly, a quantitative estimate of such probability of association cannot be directly obtained from analysis of epidemiological studies. It also depends on underlying mechanisms of carcinogenesis, and how these mechanisms are affected by radiation exposure. For example, if the effect of radiation is the induction of new pre-malignant cells from healthy stem cells, it can be expected that *Z* approximates the probability of association to a large extent. If, however, radiation acts dominantly on the phase of clonal growth, it is likely that the probability of association is larger than the assigned share calculated in ProZES. These concerns about the validity of *Z* to quantify the individual or etiologic fraction of association of radiation and disease are recognized and discussed elsewhere (Greenland 1999; Greenland and Robins 1988; Beyea and Greenland 1999).

The definition of *Z* used here also implies negligible impact of the competing health risks, including radiation-attributed competing risks. It has recently been shown by Ulanowski et al. (2019) that for compound outcomes with high incidence rates and for higher doses (order of 1 Gy and above), growth of radiation-attributed risks is accompanied by reduced manifestation of spontaneous risks, thus directly affecting not only the excess rate but also the baseline rate in the exposed population.

### Limitations of ProZES

Assessment of the assigned share is a complex task and involves many elements. A number of limitations relate to necessary decisions about the general framework, epidemiological evidence and the specific risk models. These unavoidable limitations reflect current scientific state-of-the-art and do not imply that ProZES is inadequate or unsuitable for use in compensation claims. As a guideline, it was the intention to develop risk models that are based on robust epidemiological evidence and provide unbiased estimates of *Z*. In case of ambiguous data, models were selected to respect a balanced view on statistical evidence and plausibility, and, in particular, to avoid potential strong underestimations of risk, e.g. for specific ages or sex. In most cases, the limitations in data and methodology have already been addressed by assessing the uncertainty range for *Z*. A discussion of the most important limitations is provided below.

#### Risk models

Risk models for low-LET exposure are almost exclusively based on the LSS cohort, which includes individuals who experienced an acute exposure to radiation. While it would be preferable to additionally use studies of cohorts with protracted exposure, e.g. nuclear workers (Richardson et al. 2015, 2018; Leuraud et al. 2015; Daniels et al. 2017; Cardis et al. 2007; Muirhead et al. 2009), site-specific estimates still suffer from low statistical power and cannot be used for reliable risk prediction models. Nevertheless, risk estimates for all solid cancers combined are compatible with risk estimates from the atomic bomb survivors, supporting the validity of risk transfer between populations. ProZES uses the LSS incidence data with a follow-up until 1998. An update of the LSS incidence data with 11 years more of follow-up time and 29% more solid cancer cases is currently in process. At the time of this writing, risk for all solid cancers combined and for lung, breast, colon and uterine cancer was analysed (Grant et al. 2017; Cahoon et al. 2017; Brenner et al. 2018; Utada et al. 2018; Sugiyama et al. 2020). While not yet included in ProZES, it is intended to keep ProZES up to date with scientific developments.

Although the organ-specific models have been discussed previously, some models deserve special considerations:

- The lung cancer model after low-LET exposure is likely the most complex model implemented in ProZES due to the interaction of radiation and smoking. The radiation ERR of the best generalized multiplicative model decreases strongly at high smoking intensities. This could prevent compensation for heavy smokers even at high radiation exposures and might be caused by the particular functional form of this complex model. Thus, it was decided to use the maximum ERR value from different models. A further complication is missing population and health statistics data for lung cancer of never-smokers in Germany. Therefore, a generic transfer of risk between the Japanese and German population was applied, leading to additional uncertainties.
- The breast cancer model is the only low-LET model that is not exclusively based on the LSS, but on pooled data from several cohorts (Preston et al. 2002). It has the advantage that not only the LSS, but also several other cohorts with more protracted exposures and other populations are included. However, the follow-up of the cohorts used in the pooled analysis is no longer up-to-date. This applies not only to the LSS as the most important cohort in the pooled study, but, in particular, also to the Swedish haemangioma cohort, which is the second most important cohort for radiation-induced breast cancer in the world. Recently, the risk estimates for this cohort were increased by a factor of two following revision of its dosimetry system when known dosimetric issues for a part of the high-exposed women were corrected (Lundell et al. 2015; Eidemüller et al. 2015). Furthermore, due to the very different exposure situations in the cohorts, it is not clear whether derived age dependencies are a general property of radiation-induced breast cancer, or an artefact of pooling the data. Further complications can arise from differences in the biological effectiveness of lower energy X-rays in exposures of medical patients compared with high-energy photons in the LSS cohort and from comparing risks per unit dose from a single acute exposure in the LSS cohort with risks per unit dose from fractionated exposures of varying dose rates. Finally, the pooled study has no baseline parametrisation. Due to the absent model baseline rate, only a generic transfer of radiation risk to the German population can be applied. This may become problematic since breast cancer rates in Germany are higher than in Japan.
- The leukaemia risk models are among the most challenging models due to the small number of cases in combination with high radiation-attributed fractions, non-linear dose responses, strong temporal effects, sex differences, and differences in population baseline rates. To avoid strong underestimation of risk for protracted exposures, for the non-linear dose response models, the method of “twin” models was introduced (see below). The radiation risk models depend strongly on attained age, age at exposure or time since exposure. While the leukaemia models were designed to represent well the epidemiological data in the LSS cohort, due to the strong modifying effects it is not clear whether the models are robust enough to allow for risk extrapolations beyond exposure situations typical for the LSS cohort. In future updates, the ProZES leukaemia models will be re-evaluated with emphasis on models’ robustness and secure extrapolations of radiation risk.
- Lung cancer risk models for miners occupationally exposed to radon and its progeny usually demonstrate complex exposure and age dependencies (NRC 1999). They are primarily derived from highly exposed miners’ cohorts, and it is not clear if these modifying effects remain valid at lower exposures. The current risk model for ProZES is derived from a study of Wismut employees hired in 1960 or later with low exposures and is a linear exposure response model without effect modifiers (Kreuzer et al. 2015). A recent study with emphasis on temporal effects in the Wismut cohort at low radon exposures and exposure rates found evidence for modifying effects by time since and age at exposure also for low exposures (Kreuzer et al. 2018). It is foreseen that the ProZES miner radon model will be revised according to new scientific evidence. The Wismut study is based on lung cancer deaths. The risk estimates are then transferred to the incidence of lung cancer. It is assumed that the relative risk estimates and age dependencies are similar between incidence and mortality studies.
- The lung cancer risk model after indoor radon exposure (Darby et al. 2005) is linear in total exposure and has no modifying effects with time since exposure, age at exposure or attained age. While miner studies with high exposures have shown strong modifying effects, residential studies have weaker statistical evidence. More fundamentally, in residential studies, it is not possible to distinguish between effects of exposures received in different periods of the past, because radon exposures in different time periods are highly correlated (Darby et al. 2006). For ProZES, it is assumed that the risk of exposures received during a shorter period of time is the same as for the long-term exposures in the residential studies, for the same total exposure. Furthermore, breathing rates are different for light and heavy physical work and can be higher than that in residential places. An extension of the model to allow for different breathing rates is planned.

#### Transfer of risk

Radiation risk is transferred from the historic Japanese population to the current German population. The transfer of risk allows to adequately account for population-specific differences in secular trends of baseline incidence rates, and it assumes a random mixture of additive and multiplicative transfer types. As no prior knowledge on the type of risk transfer is assumed, a uniform distribution between both types was applied. Therefore, the central estimate of *Z* is associated to the mean transfer factor $f = 0.5$. If the type of risk transfer for a particular cancer site was different, then the result for the central value of assigned share would change accordingly. This is particularly relevant for cancer sites with strong differences in background cancer rates between both populations.

Future versions of ProZES may have the capability to distinguish between the two possible risk transfer types by taking into account new epidemiological evidences or biological mechanistic insights for specific cancers. Technically, this can be achieved with a non-uniform distribution function for the factor *f*, which would allow for additional or reduced weights towards pure additive ($f = 0$) or pure multiplicative ($f = 1$) types of the radiation risk transfer.

#### Multi-model inference

The current development includes a systematic use of the MMI principle. Therefore, the resulting uncertainty distribution incorporates contributions from model uncertainty. Nevertheless, the chosen models are not exhaustive, and the number and type of models depend on the selection criteria. For some cancers with strong sex differences, a mixture between sex-averaged and sex-specific risk models was applied. For exposure situations with weak epidemiological evidence due to poor statistics, e.g. for cancer at young ages, the risk models provide an extrapolation that can become more and more uncertain for more extreme situations. Use of MMI can lead to extrapolations that depend less on properties of a particular model, thus this technique translates part of this uncertainty to the error bounds of *Z*.

#### Correction factors for low dose rates and latency time

The use of a correction factor for low doses and low dose rates is under active international discussion (Rühm et al. 2015; Shore et al. 2017; Kocher et al. 2018). For ProZES, currently, only a correction factor for low dose rates (DREF) was introduced with geometric mean of 1. This is supported by the recent large workers study INWORKS (Richardson et al. 2015), whereas the approaches of the BEIR committee and of ICRP reduce the risk estimate (NRC 2006; ICRP 2007). Conditional on future international developments and recommendations, the use of the risk correction factors in ProZES for effects of low dose and low dose rate exposures might be revisited.

Cancer needs time to develop, and the latency correction factor was introduced for radiation-induced cancer which raises from zero and reaches its maximum after about 6 years for solid cancers, and 2 years for haematopoietic cancers. If the time between radiation exposure and the observed cancer is smaller, the assigned share depends on the selected functional form chosen for the latency correction factor. However, the form of the latency function cannot be derived from the epidemiological data of the LSS cohort, since the follow-up starts only 13 years after exposure for solid cancers and 5 years after exposure for hematopoietic cancers. Other known studies do not provide conclusive evidence on the cancer latency times.

#### Enhanced biological effectiveness of different radiation types

Currently, ProZES does not explicitly account for the effect of different radiation types but requires the user to provide input doses (expressed in mSv) that may already account for an enhanced biological effectiveness of different types of radiation other than high-energy gamma radiation, e.g. for low-energy photons and electrons, neutrons, protons and alpha particles other than from radon and its progeny. The uncertainty distributions used for input doses may include the contribution of uncertainties associated with radiation effectiveness.

Dose responses from major epidemiological studies, including the LSS, are representative of exposures to low-LET radiation. In particular, Japanese atomic bomb survivors received acute doses of mainly high-energy photons. In addition, several epidemiological studies are available for cases of exposure to high-LET radiation, however, they are generally limited to exposures to alpha particles (e.g. from inhalation of radon or plutonium and their decay products) and to a small number of cancer sites (e.g. lung, liver, bone). Evidence from limited human data and from more abundant animal and in vitro cell studies indicates that high-LET radiation is more effective than low-LET radiation in inducing cancer (NCRP 2012; UNSCEAR 2012). Recent analyses of data indicate that low-energy photons and electrons may also exhibit an enhanced biological effectiveness compared with high-energy photons (NCRP 2018; Kocher and Hoffman 2019).

In the system of radiological protection, absorbed doses (in Gy) are modified by quality factors or radiation weighting factors, which are intended to represent the enhanced biological effectiveness of each type of radiation relative to low-LET radiation. The current radiation weighting factors recommended by the ICRP (2007) for radiation protection of humans are 1.0 for photons, electrons and muons, 2.0 for protons and charged pions, 20 for alpha particles, fission fragments and heavy ions, and 2–20 for neutrons of various energies. These values of the radiation weighting factors are “… selected by judgement on the basis of a broad range of experimental RBE data which are relevant for stochastic effects” (ICRP 2007, paragraph 114). Correspondingly, ProZES currently accepts the dose input in terms of equivalent dose (mSv). An explicit consideration of carcinogenic effectiveness of various types of radiation can become an important topic for future advancing of ProZES.

Estimation of risks in cases of actual exposures is also carried out using modifying factors, with the difference that such factors are customized for the risk assessment of interest (e.g. adjudication of compensation claims for workers in the US; mission planning for astronauts exposed to heavy ions in space). These factors are based mostly on animal data, include estimates of uncertainty in the possible effectiveness of each radiation type, and account for differences in effectiveness of inducing leukaemia and solid cancers (Kocher et al. 2005; Cucinotta et al. 2013). In IREP, assigned share is estimated using probability distributions to describe the enhanced biological effectiveness of high-LET radiation and of low-energy photons (< 250 keV) and electrons (< 15 keV) (Kocher et al. 2005, 2008).

#### Twin models

Several risk models, in particular, for hematopoietic cancers, have a non-linear dose response which leads to problematic risk estimates for multiple and protracted exposures. Estimates of *Z* would depend on the number of exposures and might become arbitrarily small. The method of “twins” was introduced to provide stable risk estimates. For each non-linear model, a corresponding linear model is assigned, and the maximum value of either model is used, thus providing a conservative “LNT-like” risk estimate. Although the method appears reasonable for the ProZES leukaemia risk models, it is still unclear whether this procedure could be generalized to other types of non-linear relationships.

#### Non-cancer diseases

Currently, only malignant neoplasms are considered in ProZES. However, epidemiological studies provide strong evidence that radiation exposure induces cardiovascular diseases as well. Recent studies include analysis of cardiovascular mortality in the LSS cohort (Schöllnberger et al. 2018) and in a pooled cohort of nuclear workers (Gillies et al. 2017), as well as incidence of heart and cerebrovascular diseases among the Mayak workers (Simonetto et al. 2014, 2015). Notably, there is some suggestion for a non-linear dose response relationship for radiation risk, though uncertainties concerning the linearity of the dose response relationship are still large. Further complications originate from confounding factors affecting the baseline, and from the definition and classification of endpoints. Biological mechanisms underlying the disease development and radiation risk transfer to different populations are still under intense scientific debates. The same applies to the induction of cataracts by ionising radiation. Therefore, inclusion of these endpoints in the current version of ProZES was considered as premature.

Also, currently the program does not consider radiation risks of benign tumours. Following recent growing interest to establish quantitative dose response relationships for non-malignant neoplasms (SSK 2017), future development of ProZES may also include consideration of benign tumours as harmful outcomes of radiation exposure.

### Software implementation and usage

ProZES is implemented as a Windows standalone desktop application with a graphical user interface in German and English. The tool is implemented using the .NET framework^4^ and, during installation, availability of the .NET framework on the user PC is checked. If required prerequisites are missing, then the installation utility attempts to automatically download and install the required software.

**Fig. 5:**
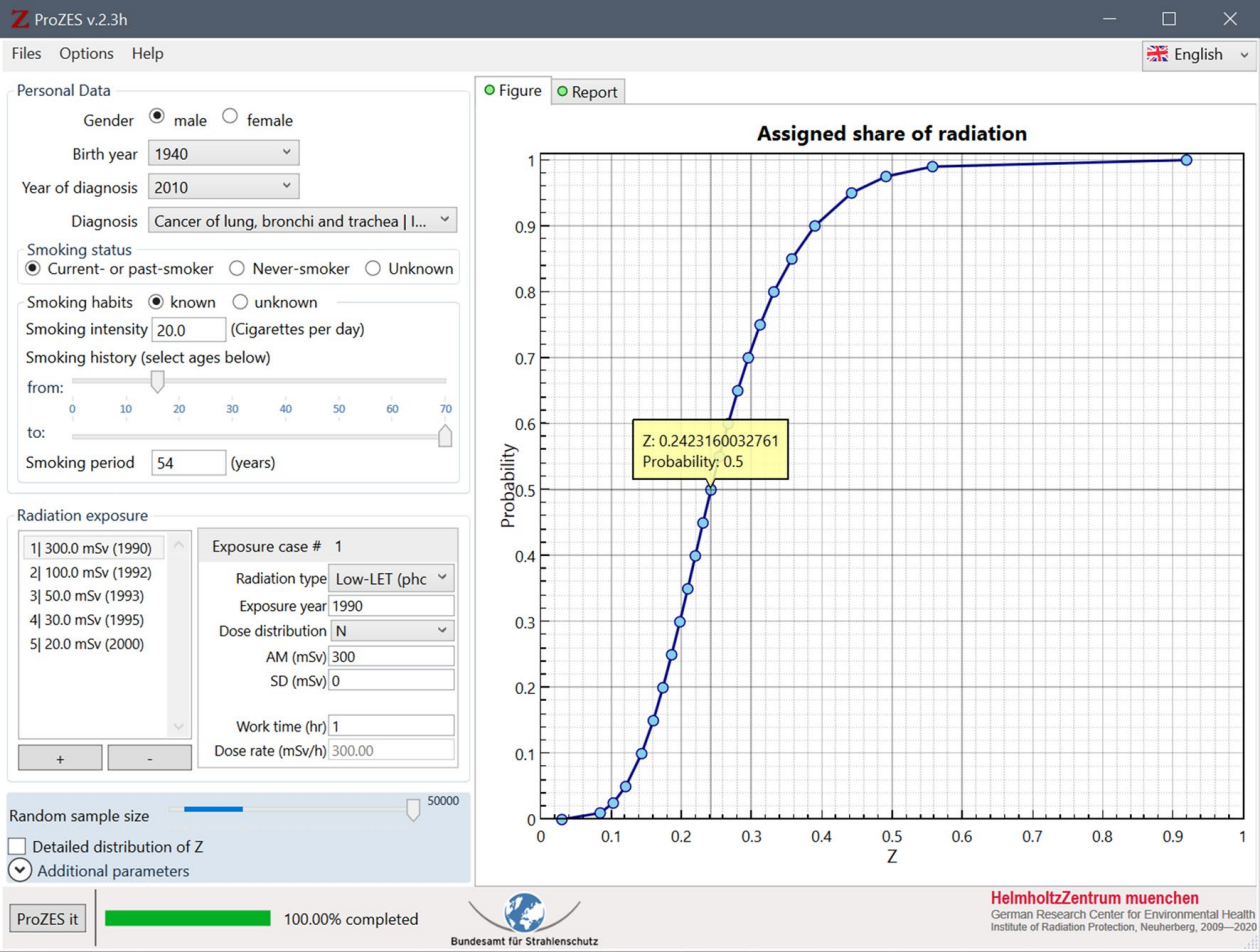
Screenshot of the ProZES tool running a lung cancer case for a current smoker with exposure history. A tab with a plot of the cumulative distribution of *Z* is selected and displayed in the right panel. The dots indicate the percentiles of the distribution

**Fig. 6:**
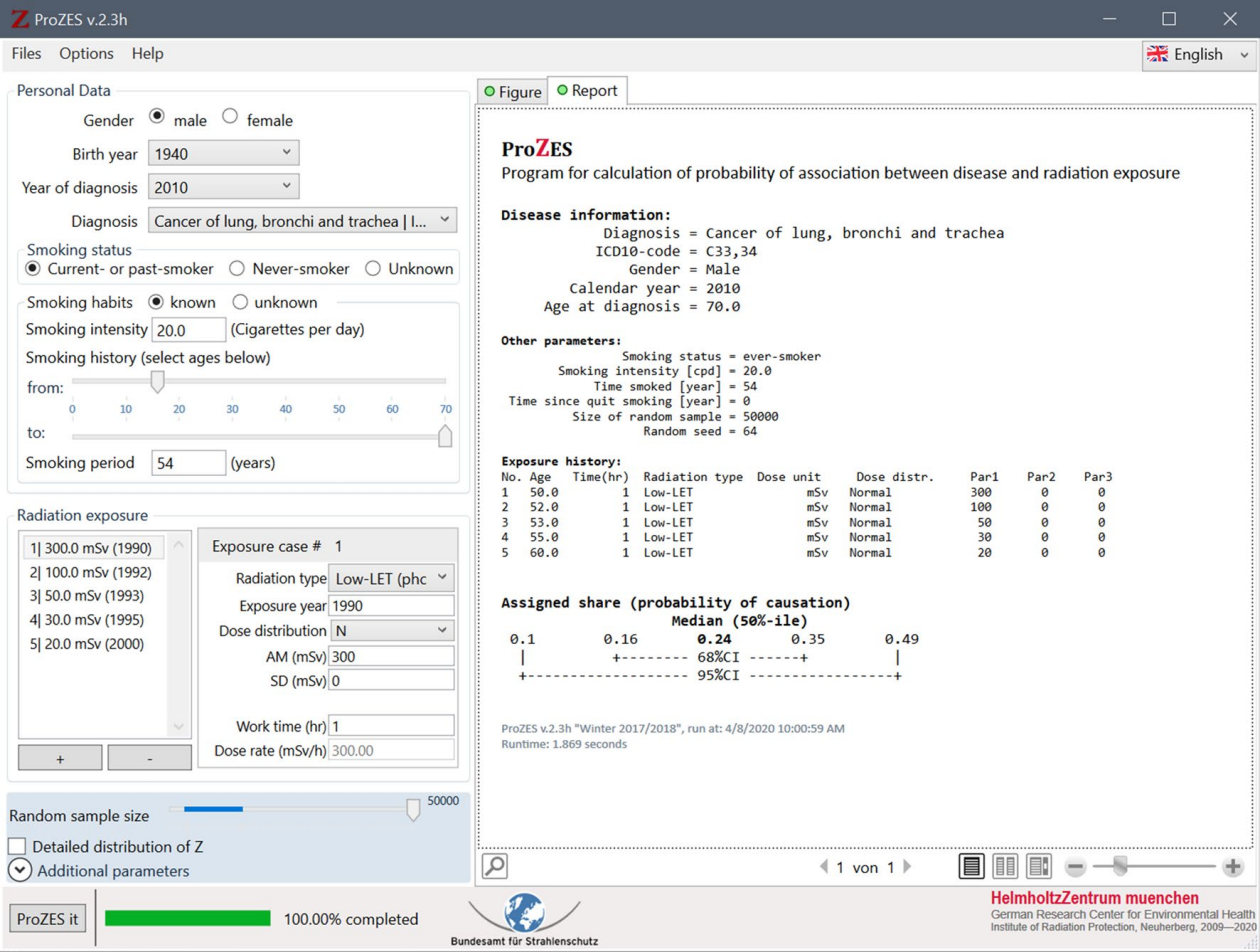
Screenshot of the ProZES tool running a lung cancer case for a current smoker with exposure history. A tab with the summary report (concise form) is selected and displayed in the right panel

Starting the calculation, from the user-provided input data the tool generates a random sample of assigned share values and presents the results in graphical and text forms (see Figs. 5 and 6). The assigned share is displayed as a cumulative probability distribution. The output report with a summary of the input and the output statistics is generated and can be viewed either in concise form with the central estimate and 68%- and 95%-confidence intervals or in the expanded form with detailed percentiles of *Z*. The plot and the output report can be saved either as bitmap (PNG) or vector (PDF) images. A user manual is available under the program’s “Help” menu. The user can vary the sample size to give more emphasis to the speed of the calculation or to produce more precise evaluation of the percentiles.

**Fig. 7:**
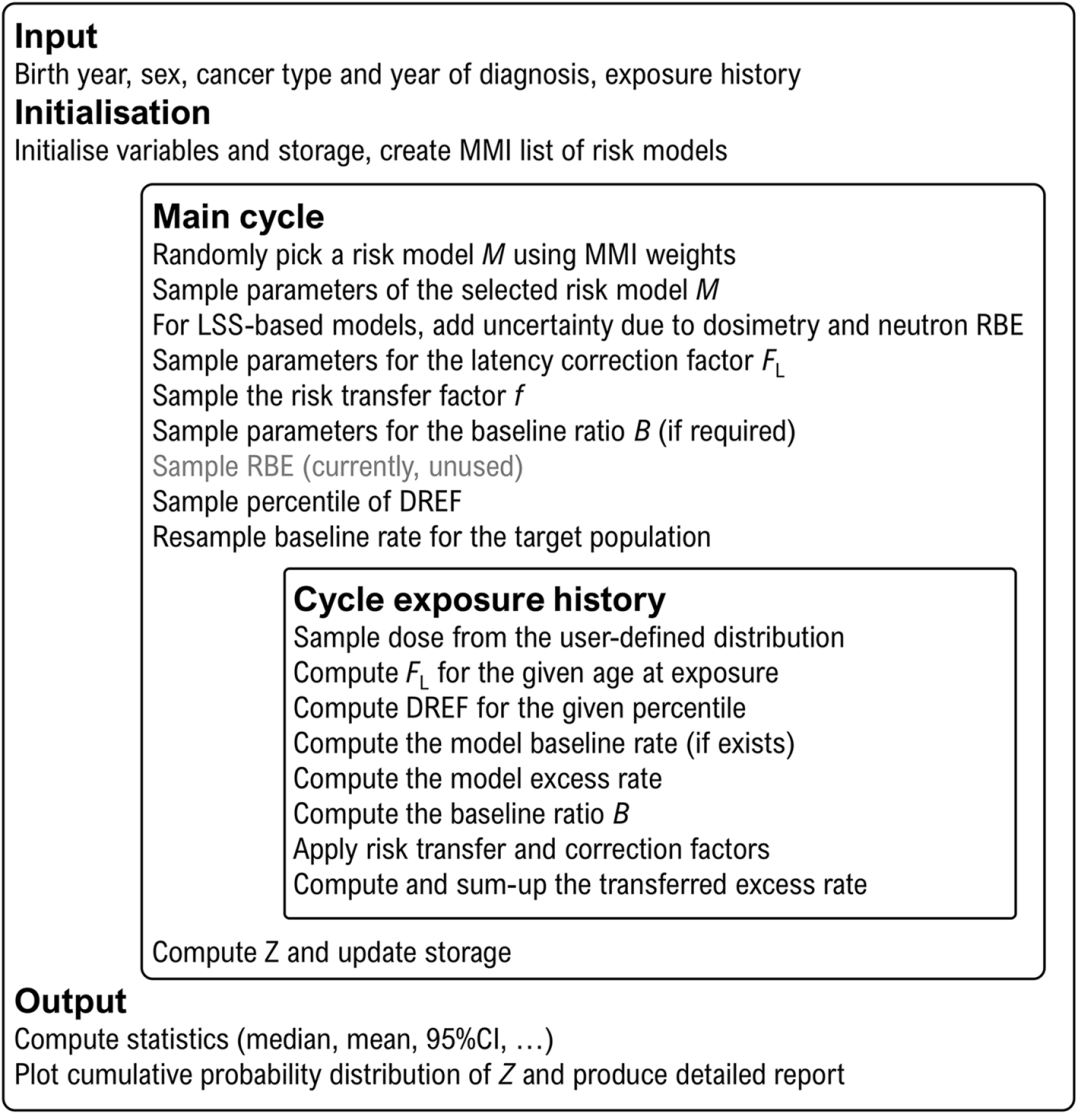
Computational flow of the ProZES software tool

Uncertainties from various sources are combined using Monte Carlo simulations and reported as a probability distribution of the assigned share. In case of multiple exposure events, each exposure is described by an organ dose (mSv) that has its own uncertainty and it is sampled uncorrelated to doses from other exposures. For lung cancer after inhalation of radon, exposures, specified in WLM or Bq/m^3^, are also treated as independent events in the exposure history. Sources of uncertainties implemented in ProZES include statistical uncertainties in parameters of the selected model (represented by the corresponding covariance matrix), model uncertainty given by the MMI weights, and parameter uncertainties related to DREF, latency factor, and population transfer factor. While each parameter set is sampled independently and uncorrelated to the others, the parameter sets are assumed to be fully correlated among various exposures. The principal flow of computation is shown in Fig. 7.

### Input to ProZES

The following user-defined input is required to run the ProZES tool:

- Cancer type and year of diagnosis
- Sex and birth year
- History of radiation exposure including the radiation doses and year of each exposure. For indoor exposure to radon, also the working time (hours) is required. The doses are provided in mSv for exposures to low-LET ionising radiation, and in WLM or Bq m^−3^ for exposures to radon and progeny from underground/mines and indoor exposures, respectively.

For each exposure, a probability distribution can be specified to describe the uncertainty in dose. The currently implemented uncertainty distribution types are Gaussian (normal), log-normal, uniform and triangular. In the case of small dose rates below 6 mSv h^−1^, that can be calculated from user-provided duration of each exposure (hours), the corresponding DREF distribution is used for low-LET radiation (Fig. 1). For exposures to radon in underground/mines or indoor a DREF correction is not applied (i.e., assumed to be equal to 1.0).

For lung cancer only, the user can provide information on smoking history. For a current or past smoker, the smoking intensity and period can be provided. If individual smoking history for a current or past smoker is unknown, then the computations are performed using nation-average statistics for Germany. Currently, only the risk model for lung cancer after external radiation exposure depends on the individual smoking history.

The model for lung cancer from indoor radon exposure depends on the average indoor air activity concentration (Bq m^−3^). Therefore, the total radon exposure is the product of the concentration and the exposure duration, and the risk scales linearly with the duration.

ProZES allows to load and save the input data in Excel format. A user-defined template can be created by saving the input data for a specific case. This Excel file can then be edited and loaded back into the program.

The ProZES tool allows to choose the year of cancer diagnosis up to the current calendar year. However, cancer incidence and population data are not yet available for the most recent years. Therefore, if the year of diagnosis is later than the last available year from the registries, the cancer incidence rates and population data from that last available year are used.

### ProZES and IREP

Both ProZES and IREP (Kocher et al. 2008) calculate the assigned share *Z* with associated uncertainty bounds. In both applications, the low-LET risk models are based primarily on epidemiological evidence from the LSS cohort of the Japanese atomic bomb survivors. Both programs share common central elements of the calculation such as grouping of cancer sites, transfer of risk between populations, and factors accounting for the dose rate experienced by the exposed individual and for changes of risk during the minimum latency period.

Nevertheless, there are a number of conceptual differences. First, ProZES includes a systematic use of multi-model inference. Another difference stems from the fact that IREP uses a dose and dose rate effectiveness factor (DDREF) specified as a distribution with uncertainty range extending below and above 1.0, whereas ProZES only uses a dose rate effectiveness factor with geometric mean of 1.0 and an uncertainty range conditional on the value of the dose rate. However, like in IREP, the uncertainty range of DREF in ProZES includes values less than 1.0.

As opposed to ProZES, IREP explicitly uses uncertain radiation effectiveness factors (REF) for cases of exposure to radiation types other than high-energy gamma radiation (Kocher et al. 2005, 2008). Thus, IREP can be directly applied for exposures to high-LET radiation (e.g., inhalation of plutonium or uranium; exposure to neutrons), or to low-energy photons and electrons (e.g., intake of tritium). Currently, ProZES assumes that the user-provided doses, except from exposure to radon, are given in terms of equivalent dose, and are, therefore, already adjusted for radiation type effectiveness.

For the transfer factor between populations, ProZES uses a uniform distribution between multiplicative and additive risk transfer. IREP includes a trapezoidal mixture that goes beyond the limits of $f = \left[ {0, 1} \right]$ to account for chances that the pure multiplicative risk transfer model and the pure additive risk transfer models are plausible outcomes. In ProZES, this additional uncertainty is generically accounted for by explicit modelling of the uncertainties of the parameters of the model baseline rate and use of the full covariance matrix.

The compensation system in the US is different from that used in Germany. In the US, compensation is awarded if the 99th percentile of *Z* exceeds 0.5. To ensure a good precision at the 99th percentile, IREP generates 2000 samples obtained using mid-point Latin Hypercube Sampling for its Monte Carlo simulations. The number of samples is increased to 300,000 to enhance precision for cases that produce an assigned share with a 99th percentile within 5% of the decision criteria.

The grouping of cancer sites is different in the two programs. While based on similar epidemiological low-LET data, the ProZES models are based on the LSS data with a longer follow-up and more cancer cases. Cancer groupings used in IREP include at least 50 cases among the atomic bomb survivors with doses greater than 10 mSv. In ProZES, the cancer risk models have been developed independently using the methodology described in the previous sections and are different from the IREP models. The radon model in IREP is based on U.S. uranium miner data (Kocher et al. 2008), while ProZES relies on the studies of the German Wismut workers. The method of “twin” models was introduced for the ProZES leukaemia models with a non-linear dose response relationship.

More detailed comparisons of ProZES and IREP predictions of the assigned share for various cancer sites can be found elsewhere (see Jacob et al. 2013; Ulanowski et al. 2016, Appendix 3).

## Summary and outlook

ProZES is a software tool that estimates the probability that a given cancer in an individual was caused by previous radiation exposure. This probability is called the assigned share, *Z*, and depends on the type of cancer, age at cancer diagnosis, exposure history and person-specific information such as, e.g., sex and birth year. ProZES has been developed at the Helmholtz Zentrum München and can be used to provide scientific support for expert judgements in the context of compensation claims for cancer after occupational exposure by ionising radiation. Its functionality is in many aspects similar to its US-counterpart, the program IREP (Kocher et al. 2008). However, the development of ProZES progressed independently following critical assessment of methodology and models. Its development was accompanied by intense scientific discussions with leading national and international experts.

The cancer risk models, which are the core part of ProZES, have been newly developed or re-evaluated from up-to-date radioepidemiological data. The risk models for low-LET radiation are based on incidence of cancer of the atomic bomb survivors of Hiroshima and Nagasaki (the LSS cohort), except for breast cancer where pooled data from several cohorts are used. For lung cancer after radon inhalation, separate risk models are implemented for exposure in mines and indoor exposure. ProZES includes dedicated risk models for the most frequent types of radiation-induced cancers, including cancers of the lung, female breast, colon, stomach, and thyroid. Risks for other cancers are estimated based on models developed for groupings of cancers with similar physiology.

The calculation of the assigned share has several methodological challenges that required design decisions. The most important elements are:

- Risks observed in a Japanese population during the time since the Second World War must be applied (or transferred) to a current Western population (i.e., the population of Germany). Since cancer incidence rates in the two populations can be significantly different for specific organs, and additionally can vary with calendar year, additive or multiplicative risk transfer modes can result in different values of the assigned share. In ProZES, no preference to either mode of transfer is given. All values between these two transfer modes are considered to be equally likely.
- ProZES includes a systematic use of multi-model inference which reduces the dependence on one selected model and allows accounting for model uncertainty. MMI is also applied for some problematic cancer sites to avoid potential strong underestimation or bias of *Z* for specific ages or sex.
- To account for dose rate effects, instead of applying a conventionally used DDREF, an approach based on a dose rate effectiveness factor DREF with geometric mean of 1.0 was introduced. The uncertainty of the DREF is assumed to increase with decreasing dose rate.
- Cancer groupings were formed by combining functionally similar cancer sites with compatible age dependence and risk estimates. The grouping represents a compromise between model specificity and the requirement of sufficient sample size to allow for reliable statistical inference and robust radiation risk models.
- For leukaemia models with non-linear dose responses, the method of corresponding linear “twin” models was introduced to avoid potential strong underestimation of risk and assigned share for fractionated and protracted exposures.

Particular attention was given to careful consideration of various sources of uncertainty. These include the statistical uncertainties and correlations between the parameter estimates for specific fitted models, model selection uncertainty addressed by multi-model inference, uncertainty distributions of the radiation doses and uncertainties related to the DREF, latency factor, and population transfer factor. Monte Carlo simulation was used to integrate these contributions into the total uncertainty represented by the generated distribution of *Z*.

A substantial effort has been made to incorporate the best available science during the model development. It was designed to provide evidence-based, robust and unbiased estimates of the probability of cancer causation from previous radiation exposure. With a view to its use in compensation claims, in case of ambiguous data, the selected models should avoid potential strong underestimation of risk. It is intended to keep ProZES up to date with scientific progress, including updates of risk models from extended epidemiological evidence, methodological improvements and new health statistics and demographic data.

Currently, ProZES is available as a Windows stand-alone program with a graphical user interface in German and English. Since the program has been designed for use in the Federal Republic of Germany, the calculation of *Z* is based on German national statistics on cancer incidence rates and demographic data. However, it is possible to extend its applicability to other countries as well using pertinent national data. The ProZES software tool, documentation and information on current developments are freely available from the ProZES webpage (BfS 2020) hosted by the German Federal Office for Radiation Protection.

## Electronic supplementary material

Below is the link to the electronic supplementary material.

Supplementary file1 (PDF 5383 kb)

## Footnotes

1 ProZES stands for German “**Pro**gramm zur Berechnung der **Z**usammenhangswahrscheinlichkeit einer **E**rkrankung und einer **S**trahlenexposition”, program for calculation of probability of association between disease and radiation exposure.

2 The Interactive RadioEpidemiological Program (IREP) exists in two versions, IREP-NIOSH, https://www.niosh-irep.com/irep_niosh/, and IREP-NCI, https://www.irep.nci.nih.gov/irep/.

3 Matlab and Optimization Toolbox. The Mathworks Inc., Natick, MA, USA. https://www.mathworks.com.

4 https://dotnet.microsoft.com/.
