# Supplementary material for: ProZES: the methodology and software tool for assessment of assigned share of radiation in probability of cancer occurrence
Source: Radiat Environ Biophys. 2020 Aug 26;59(4):601–29. doi: 10.1007/s00411-020-00866-7 (PMC7544726; doi:10.1007/s00411-020-00866-7)
Supplement: Supplementary file 1 — Supplementary file1 (PDF 5383 kb) [file 411_2020_866_MOESM1_ESM.pdf]

ProZES risk models

Supplement to
“ProZES – the methodology and software tool for assessment of assigned share of radiation in probability of cancer occurrence”

Alexander Ulanowski, Elena Shemiakina, Denise Güthlin, Janine Becker, Dale Preston, A. Iulian Apostoaei, F. Owen Hoffman, Peter Jacob, Jan Christian Kaiser, Markus Eidemüller

Contents

S1. Stomach cancer, group STOMACH (ICD10: C16) ... 3
Model description ... 3
S2. Colon cancer, group COLON (ICD10: C18) ... 9
Model description ... 9
Baseline ... 10
Excess risks ... 11
Group 1: Sex-specific models ... 11
Group 2: Models with common age dependence for both sexes ... 11
S3. Cancer of lung and trachea, group LUNG (ICD10: C33, C34) ... 17
Model description ... 17
Radiation-related risk of lung cancer ... 18
Model of radiation related ERR of lung cancer used in ProZES ... 19
Transfer of lung cancer risk from the LSS to the German population ... 20
S4. Female breast cancer, group BREAST (ICD10: C50) ... 30
S5. Thyroid cancer, group THYROID (ICD10: C73) ... 31
Model description ... 31
S6. Cancer of digestive organs, excluding stomach and colon, group DIG (ICD10: C00–C15, C17, C19–C26, C48) ... 34
S7. Cancer of the remaining organs, group REM (ICD10: C30–C32, C37–C41, C45–C47, C49, C62, C74–C76) ... 38
S8. Cancers of female genital organs, group GNF1 – Uterus/Cervix (ICD10: C53) ... 40
S9. Cancer of female genital organs, group GNF2 – Uterus/Corpus and other (ICD10: C51–C52, C54–C58) ... 42
S10. Cancer of male genital organs, group GNM (ICD10: C60–C61, C63) ... 44
S11. Cancer of urinary organs, group URI (ICD10: C64–C68) ... 47
S12. Cancer of brain and central nervous system, group BCNS (ICD10: C69–C72) ... 50
S13. Non-melanoma skin cancer, group SKIN (ICD10: C44) ... 52
S14. Leukaemia group HEM1 (ICD10: C91.0, C91.3, C91.9) ... 54
S15. Leukaemia group HEM2 (ICD10: C81–C86, C88, C91.1, C91.4) ... 56
S16. Leukaemia group HEM3 (ICD10: C92.0, C92.2, C92.3, C92.4, C92.5, C93–C96) ... 60
S17. Leukaemia group HEM4 (ICD10: C92.1) ... 66
S18. Lung cancer after exposure to radon in mines ... 72
S19. Lung cancer after indoor exposure to radon ... 73
References ... 74

S1. Stomach cancer, group STOMACH (ICD10: C16)

Model description

The baseline models are sex-dependent but described by the same equation:

$$\lambda_{0,fit}(10^{-5}PY^{-1}) = \exp\left(\beta_0 + \beta_1 \ln\frac{a}{70} + \beta_2 \ln^2\frac{a}{70} + \beta_3 \max^2\left(0, \ln\frac{a}{\beta_4}\right) + \beta_7(e-30)\right).$$

The baseline in the LSS cohort depends on attained age a and age at exposure e (related to birthyear). Stomach cancer rates also depend on the city of residence which are weighted according to the distribution of person years (Jacob 2013):

$$\lambda_{0,LSS} = \lambda_{0,fit}\frac{PY_H + PY_N \exp(\beta_{city})}{PY_H + PY_N},$$

where PY_H and PY_N are person years accumulated in the Hiroshima and Nagasaki sub-cohorts, respectively. For males, PY_H=731077 and PY_N=306213. For females, PY_H=1232155 and PY_N=488748. The baseline parameters are shown in Table S1.3.

The general form for the stomach cancer risk functions is represented as

$$\left.\begin{matrix} ERR \\ EAR \end{matrix}\right\} = \alpha_0 d \exp\left(\alpha_1 s + \alpha_2 \ln\frac{a}{70} + \alpha_3 \ln^2\frac{a}{70} + \alpha_4(e-30)\right),$$

where s indicates sex, and the first parameter α_0 has dimension Gy^{-1} for the ERR-type model, or $(PY\ Gy)^{-1}$ for the EAR-type model, and the parameter α_4 has dimension yr^{-1}. Table S1.1 shows the four selected risk models with their corresponding weights from multi-model inference (MMI), and the parameter values are shown in Table S1.2. Further information can be found in the first ProZES report (Jacob et al. 2013).

Table S1.1: Characteristics of the stomach cancer models selected for ProZES with MMI weights

Model/Tag	No. of cases		K	AIC	Weight
	baseline	excess			
EAR(d, a, a^2)/EAR16a^2	4577.4	125.6	16	4688.3	0.4277
EAR(d, a)/EAR15a	4570.8	132.2	15	4688.6	0.3581
ERR(d, s, a)/ERR16a	4571.6	131.4	16	4690.1	0.1741
ERR(d, s, e)/ERR16e	4576.6	126.4	16	4693.0	0.0402

Table S1.2: The MLE estimates of the risk function parameters

Model/Tag	MLE estimates for parameter:				
	α_0	α_1	α_2	α_3	α_4
EAR(d, a, a^2)/EAR16a^2	7.640×10^{-4}	–	−0.5840	−3.541	–
EAR(d, a)/EAR15a	9.152×10^{-4}	–	1.992	–	–
ERR(d, s, a)/ERR16a	0.2676	0.5451	−1.8183	–	–
ERR(d, s, e)/ERR16e	0.3215	0.5620	–	–	−0.0309

Table S1.3: Sex-dependent parameters of the baseline functions for stomach cancer models.

Model/Tag	Parameter						
	β_0	β_1	β_2	β_3	β_4, yr	β_7, yr^{-1}	β_{city}
Male							
EAR(d, a, a^2)/EAR16a^2	−5.023	4.639	−0.242	−10.46	65.4	3.5×10^{-3}	−0.150
EAR(d, a)/EAR15	−5.028	4.580	−0.313	−10.49	65.6	3.5×10^{-3}	−0.149
ERR(d, s, a)/ERR16a	−5.040	4.487	−0.413	−10.28	65.9	3.4×10^{-3}	−0.141
ERR(d, s, e)/ERR16e	−5.042	4.478	−0.365	−10.30	65.8	3.9×10^{-3}	−0.142
Female							
EAR(d, a, a^2)/EAR16a^2	−6.194	3.349	0.477	−8.78	71.8	1.40×10^{-2}	−0.150
EAR(d, a)/EAR15	−6.197	3.267	0.354	–8.50	71.9	1.40×10^{-2}	−0.149
ERR(d, s, a)/ERR16a	−6.199	3.231	0.234	−8.32	72.3	1.36×10^{-2}	−0.141
ERR(d, s, e)/ERR16e	−6.209	3.187	0.348	−8.49	72.2	1.48×10^{-2}	−0.142

Table S1.4: Covariance matrix for the model STOMACH-ERR16a for males.

	β_0	β_1	β_2	β_3	β_4	β_7	β_{city}	α_0	α_1	α_2
β_0	4,716E-03	3,050E-02	3,392E-02	5,280E-02	-1,870E-01	-3,000E-05	-3,046E-04	1,498E-03	-4,730E-04	-1,011E-03
β_1	3,050E-02	2,614E-01	3,222E-01	1,185E-01	-1,222E+00	-2,733E-04	-1,213E-05	-2,001E-03	7,510E-04	3,625E-03
β_2	3,392E-02	3,222E-01	4,688E-01	-5,930E-03	-1,402E+00	-1,928E-04	-1,594E-05	3,537E-03	4,205E-04	2,295E-02
β_3	5,280E-02	1,185E-01	-5,930E-03	7,563E+00	-6,464E+00	5,713E-04	3,457E-04	-1,363E-03	-1,385E-04	-8,670E-03
β_4	-1,870E-01	-1,222E+00	-1,402E+00	-6,464E+00	1,113E+01	-3,855E-05	1,159E-04	-3,925E-03	-1,296E-04	-1,987E-02
β_7	-3,000E-05	-2,733E-04	-1,928E-04	5,713E-04	-3,855E-05	3,047E-06	1,872E-06	3,850E-06	-9,269E-07	5,249E-07
β_{city}	-3,046E-04	-1,213E-05	-1,594E-05	3,457E-04	1,159E-04	1,872E-06	1,191E-03	1,548E-05	5,940E-05	2,964E-04
α_0	1,498E-03	-2,001E-03	3,537E-03	-1,363E-03	-3,925E-03	3,850E-06	1,548E-05	6,403E-02	-1,140E-02	-1,007E-02
α_1	-4,730E-04	7,510E-04	4,205E-04	-1,385E-04	-1,296E-04	-9,269E-07	5,940E-05	-1,140E-02	6,236E-03	2,503E-02
α_2	-1,011E-03	3,625E-03	2,295E-02	-8,670E-03	-1,987E-02	5,249E-07	2,964E-04	-1,007E-02	2,503E-02	3,746E-01

Table S1.5: Covariance matrix for the model STOMACH-ERR16a for females.

	β_0	β_1	β_2	β_3	β_4	β_7	β_{city}	α_0	α_1	α_2
β_0	1,294E-03	4,994E-03	3,027E-03	-1,394E-03	-4,657E-02	-2,803E-05	-2,951E-04	-7,796E-04	-4,420E-04	-3,090E-03
β_1	4,994E-03	7,536E-02	8,670E-02	-2,036E-01	-3,663E-01	-2,133E-04	2,129E-05	1,501E-03	6,241E-04	3,085E-03
β_2	3,027E-03	8,670E-02	1,541E-01	-3,308E-01	-4,114E-01	-9,396E-05	1,226E-05	-3,830E-03	3,556E-03	6,177E-02
β_3	-1,394E-03	-2,036E-01	-3,308E-01	1,610E+01	-9,461E+00	9,488E-05	9,132E-04	3,479E-03	-4,247E-03	-7,023E-02
β_4	-4,657E-02	-3,663E-01	-4,114E-01	-9,461E+00	1,122E+01	4,937E-05	-1,470E-04	2,607E-03	-2,370E-03	-4,132E-02
β_7	-2,803E-05	-2,133E-04	-9,396E-05	9,488E-05	4,937E-05	4,003E-06	2,030E-06	3,849E-07	6,837E-07	7,110E-06
β_{city}	-2,951E-04	2,129E-05	1,226E-05	9,132E-04	-1,470E-04	2,030E-06	1,191E-03	1,548E-05	5,940E-05	2,964E-04
α_0	-7,796E-04	1,501E-03	-3,830E-03	3,479E-03	2,607E-03	3,849E-07	1,548E-05	6,403E-02	-1,140E-02	-1,007E-02
α_1	-4,420E-04	6,241E-04	3,556E-03	-4,247E-03	-2,370E-03	6,837E-07	5,940E-05	-1,140E-02	6,236E-03	2,503E-02
α_2	-3,090E-03	3,085E-03	6,177E-02	-7,023E-02	-4,132E-02	7,110E-06	2,964E-04	-1,007E-02	2,503E-02	3,746E-01

Table S1.6: Covariance matrix for the model STOMACH-ERR16e for males.

	β_0	β_1	β_2	β_3	β_4	β_7	β_{city}	α_0	α_1	α_4
β_0	4,717E-03	3,037E-02	3,363E-02	4,899E-02	-1,822E-01	-3,119E-05	-3,067E-04	1,964E-03	-7,227E-04	4,927E-05
β_1	3,037E-02	2,599E-01	3,204E-01	9,187E-02	-1,187E+00	-2,720E-04	-2,874E-05	-3,570E-04	-6,267E-05	1,095E-04
β_2	3,363E-02	3,204E-01	4,651E-01	-3,644E-02	-1,358E+00	-1,931E-04	-3,731E-05	-2,015E-04	3,630E-06	3,710E-05
β_3	4,899E-02	9,187E-02	-3,644E-02	7,331E+00	-6,159E+00	5,668E-04	3,737E-04	-2,867E-05	4,317E-04	-2,535E-04
β_4	-1,822E-01	-1,187E+00	-1,358E+00	-6,159E+00	1,068E+01	-4,360E-05	1,712E-04	-1,158E-03	1,141E-03	-4,732E-04
β_7	-3,119E-05	-2,720E-04	-1,931E-04	5,668E-04	-4,360E-05	3,110E-06	1,716E-06	-3,587E-05	7,267E-06	2,691E-06
β_{city}	-3,067E-04	-2,874E-05	-3,731E-05	3,737E-04	1,712E-04	1,716E-06	1,192E-03	-2,429E-05	8,205E-05	-1,809E-05
α_0	1,964E-03	-3,570E-04	-2,015E-04	-2,867E-05	-1,158E-03	-3,587E-05	-2,429E-05	6,967E-02	-1,601E-02	9,510E-04
α_1	-7,227E-04	-6,267E-05	3,630E-06	4,317E-04	1,141E-03	7,267E-06	8,205E-05	-1,601E-02	8,896E-03	-7,473E-04
α_4	4,927E-05	1,095E-04	3,710E-05	-2,535E-04	-4,732E-04	2,691E-06	-1,809E-05	9,510E-04	-7,473E-04	2,737E-04

Table S1.7: Covariance matrix for the model STOMACH-ERR16e for females.

	β_0	β_1	β_2	β_3	β_4	β_7	β_{city}	α_0	α_1	α_4
β_0	1,338E-03	5,304E-03	3,583E-03	1,645E-03	-5,024E-02	-2,974E-05	-2,946E-04	-1,055E-03	-4,351E-04	2,513E-05
β_1	5,304E-03	7,694E-02	8,853E-02	-1,792E-01	-3,897E-01	-2,102E-04	3,318E-06	8,237E-04	-9,936E-05	9,405E-05
β_2	3,583E-03	8,853E-02	1,464E-01	-2,921E-01	-4,294E-01	-1,016E-04	-1,358E-05	-5,587E-04	5,198E-04	-1,820E-04
β_3	1,645E-03	-1,792E-01	-2,921E-01	1,638E+01	-9,838E+00	5,634E-05	1,037E-03	-6,399E-03	2,689E-03	-1,227E-03
β_4	-5,024E-02	-3,897E-01	-4,294E-01	-9,838E+00	1,151E+01	3,064E-05	-6,387E-05	-1,795E-03	1,660E-03	-5,822E-04
β_7	-2,974E-05	-2,102E-04	-1,016E-04	5,634E-05	3,064E-05	4,325E-06	1,572E-06	5,869E-05	-1,420E-05	8,565E-06
β_{city}	-2,946E-04	3,318E-06	-1,358E-05	1,037E-03	-6,387E-05	1,572E-06	1,192E-03	-2,429E-05	8,205E-05	-1,809E-05
α_0	-1,055E-03	8,237E-04	-5,587E-04	-6,399E-03	-1,795E-03	5,869E-05	-2,429E-05	6,967E-02	-1,601E-02	9,510E-04
α_1	-4,351E-04	-9,936E-05	5,198E-04	2,689E-03	1,660E-03	-1,420E-05	8,205E-05	-1,601E-02	8,896E-03	-7,473E-04
α_4	2,513E-05	9,405E-05	-1,820E-04	-1,227E-03	-5,822E-04	8,565E-06	-1,809E-05	9,510E-04	-7,473E-04	2,737E-04

Table S1.8: Covariance matrix for the model STOMACH-EAR15a for males.

	β_0	β_1	β_2	β_3	β_4	β_7	β_{city}	α_0	α_2
β_0	5,244E-03	3,424E-02	3,797E-02	5,216E-02	-1,968E-01	-3,119E-05	-3,203E-04	-4,778E-07	-1,482E-03
β_1	3,424E-02	2,887E-01	3,548E-01	9,612E-02	-1,276E+00	-2,825E-04	-4,132E-05	1,733E-06	-6,323E-03
β_2	3,797E-02	3,548E-01	5,117E-01	-4,448E-02	-1,453E+00	-1,995E-04	-3,359E-05	2,941E-06	1,525E-03
β_3	5,216E-02	9,612E-02	-4,448E-02	7,229E+00	-6,050E+00	5,816E-04	3,251E-04	-1,175E-05	-2,469E-02
β_4	-1,968E-01	-1,276E+00	-1,453E+00	-6,050E+00	1,076E+01	-4,596E-05	2,015E-04	-1,556E-06	2,642E-02
β_7	-3,119E-05	-2,825E-04	-1,995E-04	5,816E-04	-4,596E-05	3,142E-06	1,897E-06	3,960E-10	4,668E-07
β_{city}	-3,203E-04	-4,132E-05	-3,359E-05	3,251E-04	2,015E-04	1,897E-06	1,257E-03	1,487E-07	6,428E-04
α_0	-4,778E-07	1,733E-06	2,941E-06	-1,175E-05	-1,556E-06	3,960E-10	1,487E-07	4,493E-08	7,026E-05
α_2	-1,482E-03	-6,323E-03	1,525E-03	-2,469E-02	2,642E-02	4,668E-07	6,428E-04	7,026E-05	3,125E-01

Table S1.9: Covariance matrix for the model STOMACH-EAR15a for females.

	β_0	β_1	β_2	β_3	β_4	β_7	β_{city}	α_0	α_2
β_0	1,428E-03	6,177E-03	4,239E-03	6,600E-03	-6,049E-02	-3,060E-05	-3,080E-04	-1,442E-06	-2,347E-03
β_1	6,177E-03	8,942E-02	1,044E-01	-1,699E-01	-4,740E-01	-2,281E-04	-1,628E-05	5,567E-07	-7,665E-03
β_2	4,239E-03	1,044E-01	1,760E-01	-3,033E-01	-5,275E-01	-1,007E-04	5,502E-06	3,568E-06	1,171E-02
β_3	6,600E-03	-1,699E-01	-3,033E-01	1,649E+01	-1,033E+01	9,375E-05	1,023E-03	-3,533E-06	3,042E-03
β_4	-6,049E-02	-4,740E-01	-5,275E-01	-1,033E+01	1,266E+01	5,775E-05	-1,721E-05	-1,855E-06	1,181E-02
β_7	-3,060E-05	-2,281E-04	-1,007E-04	9,375E-05	5,775E-05	4,282E-06	1,869E-06	1,045E-08	8,667E-06
β_{city}	-3,080E-04	-1,628E-05	5,502E-06	1,023E-03	-1,721E-05	1,869E-06	1,257E-03	1,487E-07	6,428E-04
α_0	-1,442E-06	5,567E-07	3,568E-06	-3,533E-06	-1,855E-06	1,045E-08	1,487E-07	4,493E-08	7,026E-05
α_2	-2,347E-03	-7,665E-03	1,171E-02	3,042E-03	1,181E-02	8,667E-06	6,428E-04	7,026E-05	3,125E-01

Table S1.10: Covariance matrix for the model STOMACH-EAR16a[2] for males.

	β_0	β_1	β_2	β_3	β_4	β_7	β_{city}	α_0	α_2	α_3
β_0	5,484E-03	3,568E-02	3,937E-02	5,225E-02	-2,026E-01	-3,111E-05	-3,249E-04	-1,344E-06	-1,085E-02	-1,057E-02
β_1	3,568E-02	2,977E-01	3,634E-01	9,342E-02	-1,304E+00	-2,811E-04	-8,245E-05	-5,622E-06	-1,007E-01	-1,285E-01
β_2	3,937E-02	3,634E-01	5,193E-01	-4,897E-02	-1,473E+00	-1,973E-04	-6,933E-05	-4,764E-06	-1,203E-01	-1,842E-01
β_3	5,225E-02	9,342E-02	-4,897E-02	6,961E+00	-5,898E+00	5,751E-04	3,502E-04	-6,649E-06	4,668E-02	1,211E-01
β_4	-2,026E-01	-1,304E+00	-1,473E+00	-5,898E+00	1,077E+01	-5,259E-05	3,204E-04	1,854E-05	2,153E-01	2,106E-01
β_7	-3,111E-05	-2,811E-04	-1,973E-04	5,751E-04	-5,259E-05	3,139E-06	1,899E-06	-7,858E-10	-2,710E-05	-4,578E-05
β_{city}	-3,249E-04	-8,245E-05	-6,933E-05	3,502E-04	3,204E-04	1,899E-06	1,256E-03	3,111E-07	2,395E-03	1,815E-03
α_0	-1,344E-06	-5,622E-06	-4,764E-06	-6,649E-06	1,854E-05	-7,858E-10	3,111E-07	7,327E-08	4,647E-04	4,701E-04
α_2	-1,085E-02	-1,007E-01	-1,203E-01	4,668E-02	2,153E-01	-2,710E-05	2,395E-03	4,647E-04	5,329E+00	6,934E+00
α_3	-1,057E-02	-1,285E-01	-1,842E-01	1,211E-01	2,106E-01	-4,578E-05	1,815E-03	4,701E-04	6,934E+00	1,042E+01

Table S1.11: Covariance matrix for the model STOMACH-EAR16a[2] for females.

	β_0	β_1	β_2	β_3	β_4	β_7	β_{city}	α_0	α_2	α_3
β_0	1,509E-03	6,831E-03	4,925E-03	1,022E-02	-6,520E-02	-3,059E-05	-3,126E-04	-2,427E-06	-1,444E-02	-1,349E-02
β_1	6,831E-03	9,602E-02	1,111E-01	-1,513E-01	-5,000E-01	-2,273E-04	-6,972E-05	-1,092E-05	-1,677E-01	-2,235E-01
β_2	4,925E-03	1,111E-01	1,812E-01	-2,825E-01	-5,429E-01	-9,911E-05	-5,438E-05	-1,067E-05	-2,264E-01	-3,625E-01
β_3	1,022E-02	-1,513E-01	-2,825E-01	1,627E+01	-1,022E+01	9,911E-05	1,249E-03	4,653E-05	6,991E-01	9,887E-01
β_4	-6,520E-02	-5,000E-01	-5,429E-01	-1,022E+01	1,227E+01	5,076E-05	1,545E-04	3,177E-05	3,713E-01	4,367E-01
β_7	-3,059E-05	-2,273E-04	-9,911E-05	9,911E-05	5,076E-05	4,262E-06	1,872E-06	1,362E-08	4,500E-05	2,480E-05
β_{city}	-3,126E-04	-6,972E-05	-5,438E-05	1,249E-03	1,545E-04	1,872E-06	1,256E-03	3,111E-07	2,395E-03	1,815E-03
α_0	-2,427E-06	-1,092E-05	-1,067E-05	4,653E-05	3,177E-05	1,362E-08	3,111E-07	7,327E-08	4,647E-04	4,701E-04
α_2	-1,444E-02	-1,677E-01	-2,264E-01	6,991E-01	3,713E-01	4,500E-05	2,395E-03	4,647E-04	5,329E+00	6,934E+00
α_3	-1,349E-02	-2,235E-01	-3,625E-01	9,887E-01	4,367E-01	2,480E-05	1,815E-03	4,701E-04	6,934E+00	1,042E+01

S2. Colon cancer, group COLON (ICD10: C18)

Model description

For colon cancer, the attained-age dependence of the ERR in the LSS differs considerably between males and females. However, no biological mechanism is known that supports such a difference. Furthermore, the baseline rates of males and females show similar age dependencies. To take both aspects into account it was decided to apply multi-model inference (MMI): first, a set of models with sex-specific age dependencies, and a set of models with common age dependence were selected. Each set of models was then assigned an equal weight of 50% (see Fig. S2.1).

For each group the criteria of likelihood ratio test and MMI based on AIC values were applied. Of a total of 46 models applied to the LSS data set for colon cancer, both selection criteria led to a set of six models, with five models being the same in both approaches (see Fig. S2.1). Then, both models different in both criteria were included in the final set of models. Thus, MMI was realized among a set of seven models.

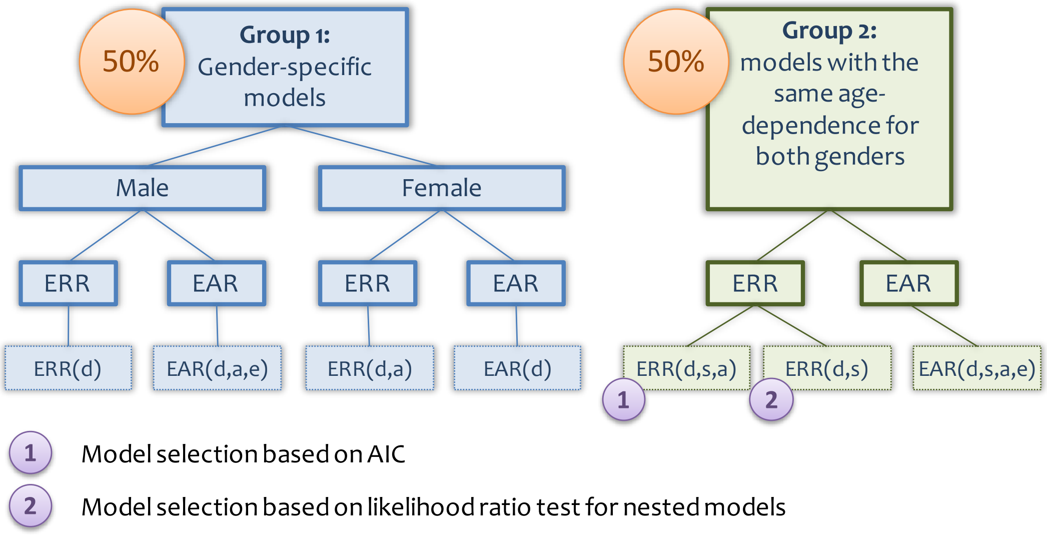

Fig. S2.1 Taxonomy of selection of colon cancer models for multi-model inference.

MMI was implemented in the following way: For given sex, the final model is constructed from five models: two models from the sex-specific models (Group 1), and three models from the group of models with common age dependence (Group 2). The models of groups 1 and 2 are given a total weight of 0.5 each.

The set of selected models together with their characteristics are summarized in Table S2.1 and described explicitly in the following sub-sections.

Table S2.1: Selected colon cancer risk models and their characteristics

Group	Model	Gender	No. of cases		K	AIC	Weight
			baseline	excess			
1	*ERR*(d)[a]	m	632.1	55.9	7	1223.3	0.5×0.9121=0.45605
1	*EAR*(d,a,e)[b]	m	634.4	53.5	9	1228.0	0.5×0.0879=0.04395
1	*ERR*(d,a)	f	800.3	19.7	9	1238.4	0.5×0.1096=0.0548
1	*EAR*(d)	f	805.9	14.1	8	1234.3	0.5×0.8904=0.4452
2	*ERR*(d,s)	m+f	1432.6	75.4	15	2464.6	0.5×0.4080=0.2040
2	*ERR*(d,s,a)[c]	m+f	1431.7	76.3	16	2464.0	0.5×0.5371=0.26855
2	*EAR*(d,s,a,e)	m+f	1434.5	73.5	17	2468.6	0.5×0.0549=0.02745

[a] 'constant' ERR model

[b] EAR model depends on attained age (a) and on age at exposure (e)

[c] ERR model with a scale factor depending on sex (s) and having common dependence on attained age (a) for both sexes

Baseline

For all selected models (see Table S2.1) the functional form of the fitted baseline is the same for given sex. Baseline for males and females are slightly different and for males it is:

$$\lambda_{0,m}(10^{-5}\ PY^{-1}) = \exp\left[\beta_0 + \beta_1 \ln\frac{a}{70} + \beta_2 \ln^2\frac{a}{70} + \beta_3 \max^2\left(0, \ln\frac{a}{\beta_4}\right) + \beta_7(e-30)\right],$$

while for females there is an additional term with parameter β_8:

$$\lambda_{0,f}(10^{-5}\ PY^{-1}) = \exp\left[\beta_0 + \beta_1 \ln\frac{a}{70} + \beta_2 \ln^2\frac{a}{70} + \beta_3 \max^2\left(0, \ln\frac{a}{\beta_4}\right) + \beta_7(e-30) + \beta_8(e-30)^2\right].$$

Parameters of the baseline functions are given in Table S2.2. Factor β_{city}, accounting for differences in baselines between the two cities, was found insignificant and is set equal to zero.

Table S2.2: Parameters of baseline functions for the selected colon cancer models.

Model		Parameter						
		β_0	β_1	β_2	β_3	β_4	β_7, yr^{-1}	β_8, yr^{-2}
		Group 1 – male						
ERR(d)	ERR7m	−5.5836	12.513	4.1526	−9.8817	53.60	−0.0713	–
EAR(d,a,e)	EAR9m	−5.5186	12.896	4.5514	−10.429	53.47	−0.0708	–
		Group 1 – female						
ERR(d,a)	ERR9f	−6.7953	9.6498	1.9637	−8.2620	62.08	−0.0590	3.69×10^{-4}
EAR(d)	EAR8f	−6.6772	10.635	3.1439	−9.3033	59.69	−0.0608	4.34×10^{-4}
		Group 2 – male						
ERR(d,s)	ERR15m	−5.5836	12.513	4.1527	−9.8818	53.60	−0.0713	–
ERR(d,s,a)	ERR16m	−5.5782	12.575	3.9071	−9.7864	53.64	−0.0713	–
EAR(d,s,a,e)	EAR17m	−5.5080	13.005	4.6323	−10.530	53.47	−0.0713	–
		Group 2 – female						
ERR(d,s)	ERR15f	−6.7332	10.256	3.6330	−9.6270	60.85	−0.0590	3.67×10^{-4}
ERR(d,s,a)	ERR16f	−6.7407	10.269	3.4968	−9.5641	60.93	−0.0590	3.69×10^{-4}
EAR(d,s,a,e)	EAR17f	−6.7044	10.501	3.7446	−9.7891	60.30	−0.0589	3.82×10^{-4}

Excess risks

For all fitted models the dimension of dose in the equations is Gy. Correspondingly, linear dose response coefficients are either Gy^{-1} for ERR-type models or $(PY\ Gy)^{-1}$ for EAR-type models.

Group 1: Sex-specific models

For males, the model with a constant ERR,

$$ERR(d) = \alpha_0 d = 1.0758\ d$$

and the following model for EAR with modifiers on attained age (a) and age at exposure (e),

$$EAR(d,a,e) = \alpha_0\ d\ \exp\left(-\alpha_3(e-30) + \alpha_5 \ln\frac{a}{70}\right)$$
$$= 1.579 \times 10^{-3}\ d\ \exp\left(-0.0818\ (e-30) + 7.805\ \ln\frac{a}{70}\right)$$

were selected.

For females, the model with a constant EAR,

$$EAR(d) = \alpha_0 d = 1.0287 \times 10^{-4}\ d$$

and the model with an ERR depending on attained age,

$$ERR(d,a) = \alpha_0\ d\ \exp\left(\alpha_5 \ln\frac{a}{70}\right) = 0.2042\ d\ \exp\left(-4.914\ \ln\frac{a}{70}\right)$$

were selected.

Group 2: Models with common age dependence for both sexes

This group consists of the three models ERR(d,s,a), ERR(d,s) and EAR(d,s,a,e) (see Fig. S2.1):

ERR(d,s,a) depends on attained age and is given by

$$ERR(d,s,a) = \alpha_0\ d\ \exp\left(\alpha_5 \ln\frac{a}{70} + \alpha_7 s\right)$$
$$= 0.555\ d\ \exp\left(-1.935\ \ln\frac{a}{70} - 0.443\ s\right)$$

The constant ERR(d,s) model is given by

$$ERR(d,s) = \alpha_0 d\ \exp(\alpha_7 s) = 0.588\ d\ \exp(-0.6041\ s) = \begin{cases} 1.0758\ d, \text{male} \\ 0.3214\ d, \text{female} \end{cases}$$

The EAR(d,s,a,e) model depends on attained age and age at exposure and is given by

$$EAR(d,s,\mathrm{a},e) = \alpha_0\ d\ \exp\left(-\alpha_3(e-30) + \alpha_5 \ln\frac{a}{70} + \alpha_7 s\right)$$
$$= 6.78 \times 10^{-4}\ d\ \exp\left(-0.0718\ (e-30) + 6.706\ \ln\frac{a}{70} - 0.784\ s\right)$$

Table S2.3: Covariance matrix for the model ERR(d) for males (ERR7m)

	β_0	β_1	β_2	β_3	β_4	β_7	α_0
β_0	2,419E-01	1,139E+00	1,101E+00	-1,117E+00	-1,608E+00	1,589E-04	-6,203E-03
β_1	1,139E+00	5,801E+00	5,878E+00	-6,315E+00	-6,814E+00	-6,714E-04	-5,014E-03
β_2	1,101E+00	5,878E+00	6,518E+00	-7,069E+00	-5,889E+00	-4,664E-04	-4,751E-03
β_3	-1,117E+00	-6,315E+00	-7,069E+00	1,016E+01	3,299E+00	1,964E-05	1,325E-02
β_4	-1,608E+00	-6,814E+00	-5,889E+00	3,299E+00	1,435E+01	-1,386E-03	-3,016E-04
β_7	1,589E-04	-6,714E-04	-4,664E-04	1,964E-05	-1,386E-03	1,587E-05	-1,351E-05
α_0	-6,203E-03	-5,014E-03	-4,751E-03	1,325E-02	-3,016E-04	-1,351E-05	6,751E-02

Table S2.4: Covariance matrix for the model EAR(d,a,e) for males (EAR9m)

	β_0	β_1	β_2	β_3	β_4	β_7	α_0	α_3	α_5
β_0	2,768E-01	1,273E+00	1,176E+00	-1,182E+00	-1,778E+00	2,041E-04	-9,405E-06	7,001E-05	-2,190E-02
β_1	1,273E+00	6,337E+00	6,132E+00	-6,581E+00	-7,375E+00	-8,083E-04	-7,409E-06	-2,608E-03	-2,801E-01
β_2	1,176E+00	6,132E+00	6,519E+00	-7,072E+00	-6,124E+00	-5,212E-04	-5,041E-06	-1,643E-03	-2,119E-01
β_3	-1,182E+00	-6,581E+00	-7,072E+00	1,049E+01	3,281E+00	6,217E-05	1,004E-05	2,081E-03	2,531E-01
β_4	-1,778E+00	-7,375E+00	-6,124E+00	3,281E+00	1,506E+01	-1,667E-03	1,660E-05	-6,776E-04	2,967E-02
β_7	2,041E-04	-8,083E-04	-5,212E-04	6,217E-05	-1,667E-03	2,015E-05	-2,712E-07	3,913E-05	1,723E-03
α_0	-9,405E-06	-7,409E-06	-5,041E-06	1,004E-05	1,660E-05	-2,712E-07	2,067E-07	-4,847E-06	7,764E-07
α_3	7,001E-05	-2,608E-03	-1,643E-03	2,081E-03	-6,776E-04	3,913E-05	-4,847E-06	7,296E-04	3,271E-02
α_5	-2,190E-02	-2,801E-01	-2,119E-01	2,531E-01	2,967E-02	1,723E-03	7,764E-07	3,271E-02	2,594E+00

Table S2.5: Covariance matrix for the model ERR(d,a) for females (ERR9f)

	β_0	β_1	β_2	β_3	β_4	β_7	β_8	α_0	α_5
β_0	3,628E-02	2,333E-01	2,875E-01	-6,086E-02	-1,050E+00	3,467E-05	-5,162E-07	7,331E-03	1,359E-01
β_1	2,333E-01	2,038E+00	2,738E+00	-1,991E+00	-6,491E+00	-1,148E-03	5,761E-05	1,024E-01	1,641E+00
β_2	2,875E-01	2,738E+00	4,605E+00	-3,838E+00	-7,491E+00	-7,350E-04	3,834E-05	1,905E-01	3,510E+00
β_3	-6,086E-02	-1,991E+00	-3,838E+00	1,120E+01	-5,025E+00	1,183E-03	-1,549E-04	-1,827E-01	-3,225E+00
β_4	-1,050E+00	-6,491E+00	-7,491E+00	-5,025E+00	3,708E+01	-4,951E-04	-1,267E-05	-2,099E-01	-3,386E+00
β_7	3,467E-05	-1,148E-03	-7,350E-04	1,183E-03	-4,951E-04	1,511E-05	-1,896E-07	-1,156E-05	-1,005E-04
β_8	-5,162E-07	5,761E-05	3,834E-05	-1,549E-04	-1,267E-05	-1,896E-07	3,057E-08	9,032E-07	8,279E-06
α_0	7,331E-03	1,024E-01	1,905E-01	-1,827E-01	-2,099E-01	-1,156E-05	9,032E-07	2,833E-02	2,892E-01
α_5	1,359E-01	1,641E+00	3,510E+00	-3,225E+00	-3,386E+00	-1,005E-04	8,279E-06	2,892E-01	4,608E+00

Table S2.6: Covariance matrix for the model EAR(d) for males (EAR8f)

	β_0	β_1	β_2	β_3	β_4	β_7	β_8	α_0
β_0	1,0618E-01	6,3332E-01	7,0906E-01	-3,7787E-01	-1,9096E+00	1,6310E-05	1,9777E-06	-7,1385E-08
β_1	6,3332E-01	4,3510E+00	5,0803E+00	-3,9687E+00	-1,0708E+01	-1,4978E-03	8,1583E-05	2,6774E-06
β_2	7,0906E-01	5,0803E+00	6,7075E+00	-5,6975E+00	-1,1290E+01	-1,0601E-03	6,2546E-05	-3,9385E-06
β_3	-3,7787E-01	-3,9687E+00	-5,6975E+00	1,0926E+01	7,5133E-01	1,6123E-03	-1,8365E-04	1,2251E-06
β_4	-1,9096E+00	-1,0708E+01	-1,1290E+01	7,5133E-01	3,9172E+01	-2,9029E-04	-1,3035E-05	-3,8568E-06
β_7	1,6310E-05	-1,4978E-03	-1,0601E-03	1,6123E-03	-2,9029E-04	1,6198E-05	-2,2695E-07	-1,7660E-08
β_8	1,9777E-06	8,1583E-05	6,2546E-05	-1,8365E-04	-1,3035E-05	-2,2695E-07	3,2359E-08	4,4640E-10
α_0	-7,1385E-08	2,6774E-06	-3,9385E-06	1,2251E-06	-3,8568E-06	-1,7660E-08	4,4640E-10	2,1844E-09

Table S2.7: Covariance matrix for the model ERR(d,s) for males (ERR15m)

	β_0	β_1	β_2	β_3	β_4	β_7	α_0	α_7
β_0	2,713E-01	1,284E+00	1,248E+00	-1,280E+00	-1,783E+00	1,793E-04	3,058E-03	-1,829E-03
β_1	1,284E+00	6,512E+00	6,595E+00	-7,104E+00	-7,689E+00	-5,693E-04	3,256E-03	-1,948E-03
β_2	1,248E+00	6,595E+00	7,239E+00	-7,855E+00	-6,783E+00	-3,634E-04	3,112E-03	-1,862E-03
β_3	-1,280E+00	-7,104E+00	-7,855E+00	1,099E+01	4,326E+00	-9,672E-05	-7,051E-03	4,218E-03
β_4	-1,783E+00	-7,689E+00	-6,783E+00	4,326E+00	1,535E+01	-1,504E-03	-1,166E-03	6,978E-04
β_7	1,793E-04	-5,693E-04	-3,634E-04	-9,672E-05	-1,504E-03	1,587E-05	6,362E-06	-3,806E-06
α_0	3,058E-03	3,256E-03	3,112E-03	-7,051E-03	-1,166E-03	6,362E-06	9,809E-02	4,053E-02
α_7	-1,829E-03	-1,948E-03	-1,862E-03	4,218E-03	6,978E-04	-3,806E-06	4,053E-02	3,395E-02

Table S2.8: Covariance matrix for the model ERR(d,s) for females (ERR15f)

	β_0	β_1	β_2	β_3	β_4	β_7	β_8	α_0	α_7
β_0	4,628E-02	2,576E-01	2,524E-01	-3,846E-02	-9,709E-01	4,142E-05	-4,295E-07	-4,689E-03	-2,758E-03
β_1	2,576E-01	1,847E+00	1,895E+00	-1,337E+00	-5,127E+00	-1,101E-03	5,698E-05	-1,786E-03	-1,050E-03
β_2	2,524E-01	1,895E+00	2,291E+00	-1,864E+00	-4,752E+00	-6,737E-04	3,573E-05	-1,677E-03	-9,862E-04
β_3	-3,846E-02	-1,337E+00	-1,864E+00	7,946E+00	-4,137E+00	1,106E-03	-1,507E-04	2,737E-03	1,610E-03
β_4	-9,709E-01	-5,127E+00	-4,752E+00	-4,137E+00	2,465E+01	-6,308E-04	5,859E-06	1,129E-02	6,639E-03
β_7	4,142E-05	-1,101E-03	-6,737E-04	1,106E-03	-6,308E-04	1,512E-05	-1,900E-07	-1,236E-07	-7,271E-08
β_8	-4,295E-07	5,698E-05	3,573E-05	-1,507E-04	5,859E-06	-1,900E-07	3,065E-08	4,007E-07	2,357E-07
α_0	-4,689E-03	-1,786E-03	-1,677E-03	2,737E-03	1,129E-02	-1,236E-07	4,007E-07	9,809E-02	4,053E-02
α_7	-2,758E-03	-1,050E-03	-9,862E-04	1,610E-03	6,639E-03	-7,271E-08	2,357E-07	4,053E-02	3,395E-02

Table S2.9: Covariance matrix for the model ERR(d,s,a) for males (ERR16m)

	β_0	β_1	β_2	β_3	β_4	β_7	α_0	α_5	α_7
β_0	2,647E-01	1,253E+00	1,224E+00	-1,250E+00	-1,772E+00	1,802E-04	1,323E-03	-1,222E-04	2,751E-02
β_1	1,253E+00	6,368E+00	6,488E+00	-6,962E+00	-7,636E+00	-5,632E-04	-1,037E-02	1,002E-02	1,864E-01
β_2	1,224E+00	6,488E+00	7,250E+00	-7,791E+00	-6,761E+00	-3,422E-04	-2,233E-02	2,383E-02	5,011E-01
β_3	-1,250E+00	-6,962E+00	-7,791E+00	1,087E+01	4,249E+00	-1,140E-04	1,312E-02	-1,541E-02	-3,572E-01
β_4	-1,772E+00	-7,636E+00	-6,761E+00	4,249E+00	1,554E+01	-1,524E-03	7,113E-03	-7,603E-03	-1,603E-01
β_7	1,802E-04	-5,632E-04	-3,422E-04	-1,140E-04	-1,524E-03	1,588E-05	6,149E-06	-2,584E-06	3,842E-05
α_0	1,323E-03	-1,037E-02	-2,233E-02	1,312E-02	7,113E-03	6,149E-06	7,535E-02	1,789E-02	-7,513E-02
α_5	-1,222E-04	1,002E-02	2,383E-02	-1,541E-02	-7,603E-03	-2,584E-06	1,789E-02	2,578E-02	6,947E-02
α_7	2,751E-02	1,864E-01	5,011E-01	-3,572E-01	-1,603E-01	3,842E-05	-7,513E-02	6,947E-02	1,212E+00

Table S2.10: Covariance matrix for the model ERR(d,s,a) for females (ERR16f)

	β_0	β_1	β_2	β_3	β_4	β_7	β_8	α_0	α_5	α_7
β_0	4,447E-02	2,479E-01	2,461E-01	-3,830E-02	-9,460E-01	4,158E-05	-5,059E-07	-4,555E-03	-1,518E-03	1,102E-02
β_1	2,479E-01	1,797E+00	1,859E+00	-1,333E+00	-5,005E+00	-1,101E-03	5,656E-05	-3,524E-03	4,906E-03	7,481E-02
β_2	2,461E-01	1,859E+00	2,302E+00	-1,891E+00	-4,676E+00	-6,711E-04	3,520E-05	-3,031E-02	7,577E-03	2,660E-01
β_3	-3,830E-02	-1,333E+00	-1,891E+00	8,017E+00	-4,188E+00	1,105E-03	-1,507E-04	1,808E-02	-6,980E-03	-1,855E-01
β_4	-9,460E-01	-5,005E+00	-4,676E+00	-4,188E+00	2,447E+01	-6,318E-04	6,448E-06	2,728E-02	-2,309E-03	-1,903E-01
β_7	4,158E-05	-1,101E-03	-6,711E-04	1,105E-03	-6,318E-04	1,512E-05	-1,902E-07	-3,402E-06	-8,435E-07	1,139E-05
β_8	-5,059E-07	5,656E-05	3,520E-05	-1,507E-04	6,448E-06	-1,902E-07	3,064E-08	4,697E-07	2,125E-07	-5,259E-07
α_0	-4,555E-03	-3,524E-03	-3,031E-02	1,808E-02	2,728E-02	-3,402E-06	4,697E-07	7,535E-02	1,789E-02	-7,513E-02
α_5	-1,518E-03	4,906E-03	7,577E-03	-6,980E-03	-2,309E-03	-8,435E-07	2,125E-07	1,789E-02	2,578E-02	6,947E-02
α_7	1,102E-02	7,481E-02	2,660E-01	-1,855E-01	-1,903E-01	1,139E-05	-5,259E-07	-7,513E-02	6,947E-02	1,212E+00

Table S2.11: Covariance matrix for the model EAR(d,s,a,e) for males (EAR17m)

	β_0	β_1	β_2	β_3	β_4	β_7	α_0	α_3	α_5	α_7
β_0	3,144E-01	1,450E+00	1,341E+00	-1,363E+00	-1,984E+00	2,351E-04	4,449E-03	-2,567E-06	-1,127E-05	-3,279E-02
β_1	1,450E+00	7,169E+00	6,904E+00	-7,423E+00	-8,363E+00	-6,704E-04	1,826E-02	-7,984E-07	-2,764E-03	-3,137E-01
β_2	1,341E+00	6,904E+00	7,234E+00	-7,848E+00	-7,057E+00	-3,828E-04	1,267E-02	-1,027E-06	-1,688E-03	-2,305E-01
β_3	-1,363E+00	-7,423E+00	-7,848E+00	1,130E+01	4,368E+00	-1,170E-04	-1,829E-02	3,773E-06	1,852E-03	2,780E-01
β_4	-1,984E+00	-8,363E+00	-7,057E+00	4,368E+00	1,606E+01	-1,825E-03	-8,126E-03	5,980E-06	-4,515E-04	6,319E-02
β_7	2,351E-04	-6,704E-04	-3,828E-04	-1,170E-04	-1,825E-03	2,029E-05	-4,460E-05	-9,860E-08	3,833E-05	1,838E-03
α_0	4,449E-03	1,826E-02	1,267E-02	-1,829E-02	-8,126E-03	-4,460E-05	8,838E-02	3,066E-05	-1,092E-03	-1,583E-01
α_3	-2,567E-06	-7,984E-07	-1,027E-06	3,773E-06	5,980E-06	-9,860E-08	3,066E-05	4,188E-08	-1,595E-06	-2,323E-05
α_5	-1,127E-05	-2,764E-03	-1,688E-03	1,852E-03	-4,515E-04	3,833E-05	-1,092E-03	-1,595E-06	6,898E-04	3,436E-02
α_7	-3,279E-02	-3,137E-01	-2,305E-01	2,780E-01	6,319E-02	1,838E-03	-1,583E-01	-2,323E-05	3,436E-02	2,802E+00

Table S2.12: Covariance matrix for the model EAR(d,s,a,e) for females (EAR17f)

	β_0	β_1	β_2	β_3	β_4	β_7	β_8	α_0	α_3	α_5	α_7
β_0	6,239E-02	3,483E-01	3,350E-01	-8,270E-02	-1,206E+00	2,589E-05	5,426E-07	1,761E-03	1,310E-07	-3,344E-04	-2,938E-02
β_1	3,483E-01	2,410E+00	2,406E+00	-1,717E+00	-6,366E+00	-1,369E-03	6,966E-05	4,089E-02	1,788E-05	-4,051E-03	-3,297E-01
β_2	3,350E-01	2,406E+00	2,838E+00	-2,295E+00	-5,776E+00	-7,892E-04	4,227E-05	1,479E-02	8,478E-06	-1,195E-03	-8,557E-02
β_3	-8,270E-02	-1,717E+00	-2,295E+00	8,192E+00	-3,404E+00	1,333E-03	-1,630E-04	-2,327E-02	-9,778E-06	2,205E-03	1,850E-01
β_4	-1,206E+00	-6,366E+00	-5,776E+00	-3,404E+00	2,746E+01	-3,213E-04	-6,681E-06	-8,380E-02	-3,786E-05	7,681E-03	6,277E-01
β_7	2,589E-05	-1,369E-03	-7,892E-04	1,333E-03	-3,213E-04	1,663E-05	-2,343E-07	-3,451E-05	-3,117E-08	1,940E-05	1,106E-03
β_8	5,426E-07	6,966E-05	4,227E-05	-1,630E-04	-6,681E-06	-2,343E-07	3,289E-08	2,717E-06	1,597E-09	-5,200E-07	-3,356E-05
α_0	1,761E-03	4,089E-02	1,479E-02	-2,327E-02	-8,380E-02	-3,451E-05	2,717E-06	8,838E-02	3,066E-05	-1,092E-03	-1,583E-01
α_3	1,310E-07	1,788E-05	8,478E-06	-9,778E-06	-3,786E-05	-3,117E-08	1,597E-09	3,066E-05	4,188E-08	-1,595E-06	-2,323E-05
α_5	-3,344E-04	-4,051E-03	-1,195E-03	2,205E-03	7,681E-03	1,940E-05	-5,200E-07	-1,092E-03	-1,595E-06	6,898E-04	3,436E-02
α_7	-2,938E-02	-3,297E-01	-8,557E-02	1,850E-01	6,277E-01	1,106E-03	-3,356E-05	-1,583E-01	-2,323E-05	3,436E-02	2,802E+00

S3. Cancer of lung and trachea, group LUNG (ICD10: C33, C34)

Model description

For radiation risk of lung cancer, the model of Furukawa et al. (2010) was selected for use in ProZES. It is based on the LSS cohort and takes into account information on smoking. An important part of the model is the interaction of radiation and smoking, as described below.

Denoting the effect of radiation exposure as $\rho(D)$, the effect of smoking as $\phi(S)$, and total and baseline lung cancer incidence rates as λ and λ_0, correspondingly, the combined effect of both factors can be described either with an additive model (AM):

$$\lambda = \lambda_0(1 + \phi(S) + \rho(D))$$

or with a multiplicative model (MM):

$$\lambda = \lambda_0(1 + \phi(S))(1 + \rho(D))\,.$$

Consequently, λ_0 is the baseline incidence rate of lung cancer among never-smokers. If both factors are not independent and there is an interaction between radiation exposure and smoking, then a generalized additive model (GAM):

$$\lambda = \lambda_0\big(1 + \phi(S) + \rho(D)\omega(S)\big) = \lambda_0(1 + \phi(S) + \rho'(D,S))$$

and a generalized multiplicative model (GMM):

$$\lambda = \lambda_0\big(1 + \phi(S))(1 + \rho(D)\omega(S)\big) = \lambda_0(1 + \phi(S))(1 + \rho'(D,S))$$

can be applied.

Table S3.1: Selection of models for risk of lung cancer using two groups of additive and multiplicative models from Furukawa et al. (2010)

Model	K	dev	AIC	Weight (%)
Simple models				
Additive (AM)	25	9428.75	9478.75	13.73
Multiplicative (MM)	25	9425.07	9475.07	86.27
Generalized models				
Additive (GAM)	27	9415.70	9469.70	6.21
Multiplicative (GMM)	27	9410.27	9464.27	93.79

Furukawa et al. (2010) built their models using an extended LSS cohort, which included lung cancer cases being absent in the city at the time of bombing. Correspondingly, the baseline was modelled by accounting for place of residence (Hiroshima or Nagasaki) and presence in either city at the time of detonation:

$$\lambda_0(10^{-4}PY) = \exp\left[\beta_{0,s} + \beta_{city}(c-1) + \beta_1 \ln\frac{a}{70} + \beta_2 \ln^2\frac{a}{70} + \beta_7\frac{e-30}{10} + \beta_9(c)NIC\right]$$

where c is the city index

$$c = \begin{cases} 1, & \text{for Hiroshima} \\ 2, & \text{for Nagasaki} \end{cases}$$

and the index *NIC* (‘not-in-the-city’ status) equals to zero for 23.46% of the cohort members and equals to one, otherwise. In the total cohort, 28.8% of members were residents of Nagasaki, others resided in Hiroshima.

The modified radiation effect is modelled as follows:

$$\rho'(D,S) = \alpha_0 D \exp\left[\alpha_3\frac{e-30}{10} + \alpha_5 \ln\frac{a}{70} + \alpha_8 \ln\left(\frac{cpd}{20}+1\right) + \alpha_9 \ln^2\left(\frac{cpd}{20}+1\right) + \alpha_{10}s\right]$$

where *cpd* is the smoking intensity, i.e. the number of cigarettes smoked per day. Correspondingly, the simple radiation-only effect is represented as $\rho(D) = \rho'(D,0)$.

The modelling of the smoking effect, (S), depends on individual smoking habits. For never-smokers, the smoking effect is apparently zero:

$$\phi(S) = 0 \text{ and } \rho'(D,S) = \rho(D)\ .$$

For current and past smokers (also called ever-smokers), modelling of the smoking effect depends on availability of information on smoking habits. That is, when information on smoking habits is available, then the function for the smoking effect appears as follows:

$$\phi(S) = \frac{\Pi}{50}\exp\left[\delta_{1,s} + \delta_2\frac{e-30}{10} + \delta_3 \ln\frac{t_s}{50} + \delta_4 \ln^2\frac{t_s}{50} + \delta_5 \ln(t_q+1)\right]$$

where Π is the smoking ‘dose’ (pack-year), t_s is the smoking duration (year), t_q is the number of years since quit smoking for ex-smokers. For persons with unknown smoking status, the average smoking effect is modelled as a constant factor:

$$\phi(S) = \exp(\delta_0)$$

where δ_0 is a constant dependent on sex and birth cohort (expressed in case of the LSS cohort via age at exposure e).

Radiation-related risk of lung cancer

The relative risk of radiation-induced lung cancer can be expressed as follows:

$$RR(D,S) = \frac{\lambda}{\lambda_0(1+\phi(S))}$$

i.e. the equations for the relative risk are different for additive and multiplicative models:

$$RR(D,S) = \frac{1 + \phi(S) + \rho'(D,S)}{1 + \phi(S)} \text{ (additive)},$$

$$RR(D,S) = 1 + \rho'(D,S) \text{ (multiplicative)}.$$

The excess relative risk due to radiation is correspondingly:

$$ERR_{rad} = \frac{\rho'(D,S)}{1 + \phi(S)} \text{ (additive)},$$

$$ERR_{rad} = \rho'(D,S) \text{ (multiplicative)}.$$

Applying multi-model inference, the risk for generalized models with non-zero interaction between radiation and smoking can be written as:

$$ERR_{rad,G} = \rho'(D,S)\left(\frac{w_A}{1 + \phi(S)} + w_M\right)$$

and for simple models with independently acting factors for radiation and smoking:

$$ERR_{rad,S} = \rho(D)\left(\frac{w_A}{1 + \phi(S)} + w_M\right),$$

where w_A and w_M are the AIC-weights of the additive and multiplicative models, correspondingly.

Model of radiation related ERR of lung cancer used in ProZES

Based on AIC alone, the simple models are inferior to the generalized ones and would not be used for the final MMI. The generalized models can better express the complex interaction between radiation and smoking. However, for large smoking intensities the functional form of the generalized models predicts a vanishing ERR. Although this might reflect the large influence of heavy smoking and strongly increased baseline risk, it is questionable if such a strong decrease in relative radiation risk is plausible. Therefore, with the aim to be used in compensation claims, the following model is suggested for ProZES:

$$ERR_{rad} = \max\left(ERR_{rad,S}, ERR_{rad,G}\right).$$

As a result, for large smoking intensities the ERR levels to a constant value instead of going to zero. This ensures that also heavy smokers might get compensated after occupational exposure.

For never-smokers only generalized models are used because generalized models are preferable based on AIC, and without smoking term they are indistinguishable from the simple ones.

Implementation of the model of lung cancer depends on availability of information on personal smoking habits. If such information is absent, then ProZES accounts for German-specific behavioral patterns regarding smoking (Schulze and Lampert, 2006). If personal smoking status is unknown, then random sampling is applied to decide whether the person should be regarded as never-smoker (35% of males and 53% of females), or ever-smoker (65% of males and 47% of females), for which average smoking habits are assumed based on personal age and birth cohort (Tables S3.2 and S3.3).

Table S3.2: Adopted average smoking intensity for smokers in Germany based on data from (Schulze and Lampert, 2006)

Age	Gender-averaged smoking intensity (cpd)	
	Males	Females
18–19	13.3	10.3
20–29	15.6	12.0
30–39	18.5	14.3
40–49	20.2	15.6
50–59	18.6	14.4
60–69	16.3	12.5
70–79	11.0	8.4

Table S3.3: Sex-specific median ages of start and quit smoking in Germany for various birth cohorts based on data from (Schulze and Lampert, 2006)

Birthyear	Start smoking		Quit smoking	
	male	female	male	female
1921-25	20	22	≈55	≈63
1941-45	19	20–21	≈55	≈63
1961-65	17	16.5	≈55	≈50–55
1976-80	16	16	n.d.	n.d.

Transfer of lung cancer risk from the LSS to the German population

The lung cancer model takes into account radiation and smoking effects; thus, the baseline incidence rate is defined for non-exposed never-smokers. Unfortunately, these data are not readily available for the German population. Therefore, the transfer factor is modelled stochastically with the assumption that the ratio of baselines is log-uniformly distributed in range from 1/3 to 3, as outlined in the methodology section.

Table S3.4: Parameters and their statistics for lung cancer risk models

Parameter	Simple models				Generalized models			
	AM		MM		GAM		GMM	
	Mean	SD	Mean	SD	Mean	SD	Mean	SD
$\beta_{0,m}$	2,29E+00	1,23E-01	2,31E+00	1,16E-01	2,29E+00	1,23E-01	2,26E+00	1,21E-01
$\beta_{0,f}$	1,68E+00	7,50E-02	1,71E+00	7,13E-02	1,68E+00	7,44E-02	1,71E+00	7,17E-02
β_{city}	2,01E-01	5,96E-02	2,08E-01	5,96E-02	2,03E-01	5,96E-02	2,09E-01	5,96E-02
β_7	-1,39E-01	4,44E-02	-1,31E-01	4,22E-02	-1,49E-01	4,40E-02	-1,35E-01	4,25E-02
$\beta_{9,Hi}$	-8,10E-02	6,75E-02	-6,70E-02	6,78E-02	-7,77E-02	6,75E-02	-6,57E-02	6,76E-02
$\beta_{9,Na}$	-1,01E-01	1,12E-01	-9,48E-02	1,12E-01	-1,01E-01	1,12E-01	-9,63E-02	1,12E-01
$\beta_{1,m}$	4,98E+00	3,59E-01	4,94E+00	3,56E-01	4,93E+00	3,59E-01	4,94E+00	3,59E-01
$\beta_{1,f}$	4,99E+00	3,24E-01	4,95E+00	3,24E-01	5,02E+00	3,25E-01	4,96E+00	3,24E-01
$\beta_{2,m}$	-5,05E+00	1,03E+00	-5,02E+00	1,03E+00	-4,94E+00	1,03E+00	-5,00E+00	1,03E+00
$\beta_{2,f}$	-2,03E+00	8,91E-01	-1,99E+00	8,83E-01	-1,93E+00	8,77E-01	-1,96E+00	8,77E-01
α_0	9,75E-01	2,38E-01	6,87E-01	1,50E-01	6,43E-01	1,98E-01	5,91E-01	1,72E-01
α_3	2,76E-01	1,59E-01	2,31E-01	1,51E-01	4,07E-01	1,65E-01	2,58E-01	1,54E-01
α_5	-2,67E+00	1,16E+00	-2,49E+00	1,15E+00	-3,04E+00	1,19E+00	-2,79E+00	1,18E+00
α_8	0,00E+00	0,00E+00	0,00E+00	0,00E+00	1,02E+01	2,62E+00	9,17E+00	2,66E+00
α_9	0,00E+00	0,00E+00	0,00E+00	0,00E+00	-1,53E+01	5,50E+00	-1,67E+01	5,68E+00
α_{10}	2,94E-01	1,98E-01	5,50E-01	1,37E-01	5,42E-01	1,61E-01	5,16E-01	1,39E-01
$\delta_{1,m}$	1,25E+00	1,71E-01	1,19E+00	1,64E-01	1,26E+00	1,70E-01	1,28E+00	1,66E-01
$\delta_{1,f}$	1,96E+00	1,61E-01	1,83E+00	1,57E-01	1,86E+00	1,72E-01	1,76E+00	1,70E-01
δ_2	3,21E-01	7,79E-02	3,09E-01	7,54E-02	3,15E-01	7,83E-02	2,85E-01	7,50E-02
δ_3	-3,00E-01	4,78E-01	-2,57E-01	4,72E-01	-2,19E-01	4,88E-01	-2,41E-01	4,71E-01
δ_4	-2,59E+00	1,21E+00	-2,55E+00	1,18E+00	-2,82E+00	1,30E+00	-2,50E+00	1,18E+00
δ_5	-5,03E-01	1,15E-01	-4,92E-01	1,13E-01	-4,85E-01	1,15E-01	-4,66E-01	1,08E-01
$\delta_{0,m,1}$	1,77E-01	3,30E-01	1,43E-01	3,22E-01	1,34E-01	3,34E-01	2,13E-01	3,19E-01
$\delta_{0,m,2}$	2,47E-01	3,31E-01	1,61E-01	3,30E-01	2,56E-01	3,27E-01	2,44E-01	3,22E-01
$\delta_{0,m,3}$	3,96E-01	2,75E-01	3,47E-01	2,64E-01	4,64E-01	2,67E-01	4,38E-01	2,60E-01
$\delta_{0,f,1}$	-1,00E+01	0,00E+00	-1,00E+01	0,00E+00	-1,00E+01	0,00E+00	-1,00E+01	0,00E+00
$\delta_{0,f,2}$	-1,42E+00	6,06E-01	-1,97E+00	9,01E-01	-1,39E+00	5,88E-01	-1,86E+00	8,22E-01
$\delta_{0,f,3}$	-9,92E-01	5,06E-01	-1,29E+00	5,87E-01	-9,33E-01	4,84E-01	-1,20E+00	5,50E-01

Table S3.5: Covariance matrix for AM lung cancer model.

	$\beta_{0,m}$	$\beta_{0,f}$	β_{city}	β_7	$\beta_{9,Hi}$	$\beta_{9,Na}$	$\beta_{1,m}$	$\beta_{1,f}$	$\beta_{2,m}$	$\beta_{2,f}$	α_0	α_3	α_5
$\beta_{0,m}$	1,521E-02	9,430E-04	-1,142E-03	2,691E-05	-1,234E-03	-1,890E-04	-3,249E-03	-7,953E-04	-2,707E-02	-3,884E-03	-8,935E-03	7,561E-04	-8,764E-03
$\beta_{0,f}$	9,430E-04	5,618E-03	-1,006E-03	3,461E-04	-1,246E-03	-2,147E-04	-1,095E-03	-3,916E-03	-1,913E-03	-2,731E-02	-6,968E-03	8,212E-04	-1,638E-02
β_{city}	-1,142E-03	-1,006E-03	3,556E-03	2,281E-04	1,128E-03	-2,420E-03	-1,120E-03	-7,489E-04	-3,374E-04	-3,349E-04	2,372E-04	1,351E-04	7,229E-04
β_7	2,691E-05	3,461E-04	2,281E-04	1,968E-03	5,967E-05	-1,081E-04	-6,425E-03	-7,609E-03	-3,812E-03	-3,321E-03	1,395E-04	-3,061E-03	9,868E-03
$\beta_{9,Hi}$	-1,234E-03	-1,246E-03	1,128E-03	5,967E-05	4,556E-03	3,109E-04	-1,566E-03	-5,359E-04	2,147E-04	1,648E-04	2,711E-03	-8,679E-05	3,718E-03
$\beta_{9,Na}$	-1,890E-04	-2,147E-04	-2,420E-03	-1,081E-04	3,109E-04	1,248E-02	-2,594E-04	6,793E-04	2,208E-03	1,067E-03	2,491E-03	-4,078E-04	2,736E-03
$\beta_{1,m}$	-3,249E-03	-1,095E-03	-1,120E-03	-6,425E-03	-1,566E-03	-2,594E-04	1,286E-01	3,689E-02	1,511E-01	1,865E-02	7,869E-04	9,975E-03	-7,547E-02
$\beta_{1,f}$	-7,953E-04	-3,916E-03	-7,489E-04	-7,609E-03	-5,359E-04	6,793E-04	3,689E-02	1,047E-01	1,578E-02	8,469E-02	-1,332E-03	1,201E-02	-1,209E-01
$\beta_{2,m}$	-2,707E-02	-1,913E-03	-3,374E-04	-3,812E-03	2,147E-04	2,208E-03	1,511E-01	1,578E-02	1,066E+00	4,958E-02	3,865E-03	6,628E-03	4,811E-02
$\beta_{2,f}$	-3,884E-03	-2,731E-02	-3,349E-04	-3,321E-03	1,648E-04	1,067E-03	1,865E-02	8,469E-02	4,958E-02	7,943E-01	2,289E-02	-3,649E-03	2,511E-01
α_0	-8,935E-03	-6,968E-03	2,372E-04	1,395E-04	2,711E-03	2,491E-03	7,869E-04	-1,332E-03	3,865E-03	2,289E-02	5,651E-02	-1,780E-02	1,490E-01
α_3	7,561E-04	8,212E-04	1,351E-04	-3,061E-03	-8,679E-05	-4,078E-04	9,975E-03	1,201E-02	6,628E-03	-3,649E-03	-1,780E-02	2,541E-02	-1,104E-01
α_5	-8,764E-03	-1,638E-02	7,229E-04	9,868E-03	3,718E-03	2,736E-03	-7,547E-02	-1,209E-01	4,811E-02	2,511E-01	1,490E-01	-1,104E-01	1,351E+00
α_{10}	9,349E-03	-2,592E-03	5,209E-05	-2,583E-04	-4,781E-04	-6,820E-04	-4,963E-03	3,819E-03	2,441E-03	-3,956E-03	-1,152E-02	2,452E-03	-9,822E-03
$\delta_{1,m}$	-1,877E-02	-3,380E-04	2,132E-04	1,475E-04	-5,832E-05	-3,121E-04	2,278E-03	6,220E-04	2,257E-02	4,747E-03	9,203E-03	-8,532E-04	7,495E-03
$\delta_{1,f}$	4,758E-04	-5,166E-03	1,925E-04	7,528E-04	5,005E-04	1,782E-04	-5,591E-03	-1,017E-02	3,019E-03	3,793E-03	5,731E-03	-2,097E-03	2,257E-02
δ_2	8,782E-04	-3,517E-04	-1,500E-04	-2,449E-03	-2,903E-04	-9,292E-05	5,596E-03	8,452E-03	3,591E-03	2,606E-03	-4,463E-04	3,310E-03	-9,338E-03
δ_3	6,545E-03	1,825E-03	1,148E-03	5,445E-03	1,572E-03	-2,258E-05	-1,040E-01	-3,499E-02	-1,707E-01	-3,569E-02	-2,031E-03	-7,383E-03	4,318E-02
δ_4	-9,924E-03	-3,692E-03	8,090E-04	3,821E-03	1,328E-03	-2,124E-04	-9,333E-02	-3,285E-03	-4,316E-01	-1,664E-02	5,821E-03	-4,679E-03	-2,238E-03
δ_5	-3,364E-03	-2,206E-04	2,612E-04	3,090E-04	7,814E-04	4,912E-04	-1,122E-02	-3,145E-03	-1,336E-02	-3,591E-03	1,579E-03	-4,741E-04	4,058E-03
$\delta_{0,m,1}$	-2,406E-02	7,999E-04	-1,455E-04	4,120E-03	-2,198E-03	-2,367E-03	1,851E-02	-1,057E-02	-2,986E-02	-1,377E-03	1,272E-02	-7,482E-03	1,148E-02
$\delta_{0,m,2}$	-2,424E-02	-7,160E-04	-1,204E-03	-1,944E-03	-5,509E-04	4,891E-04	2,202E-02	1,026E-02	7,742E-03	9,828E-03	1,262E-02	9,743E-04	-8,848E-04
$\delta_{0,m,3}$	-2,323E-02	-1,771E-03	-9,742E-04	-6,707E-03	-5,150E-05	8,793E-04	1,530E-02	2,644E-02	1,894E-02	1,520E-02	1,161E-02	7,536E-03	-1,623E-02
$\delta_{0,f,2}$	-7,085E-04	-2,169E-02	-2,603E-03	-5,214E-03	-1,993E-03	1,159E-03	2,070E-02	3,967E-02	9,492E-03	4,181E-02	2,196E-02	9,542E-04	1,627E-02
$\delta_{0,f,3}$	-9,882E-04	-1,774E-02	-2,661E-03	-1,353E-02	-4,520E-04	2,422E-03	4,282E-02	3,758E-02	2,479E-02	4,743E-02	1,825E-02	1,051E-02	4,044E-03

Table S3.5: Covariance matrix for AM lung cancer model. (cont'd)

	α_{10}	$\delta_{1,m}$	$\delta_{1,f}$	δ_2	δ_3	δ_4	δ_5	$\delta_{0,m,1}$	$\delta_{0,m,2}$	$\delta_{0,m,3}$	$\delta_{0,f,2}$	$\delta_{0,f,3}$
$\beta_{0,m}$	9,349E-03	-1,877E-02	4,758E-04	8,782E-04	6,545E-03	-9,924E-03	-3,364E-03	-2,406E-02	-2,424E-02	-2,323E-02	-7,085E-04	-9,882E-04
$\beta_{0,f}$	-2,592E-03	-3,380E-04	-5,166E-03	-3,517E-04	1,825E-03	-3,692E-03	-2,206E-04	7,999E-04	-7,160E-04	-1,771E-03	-2,169E-02	-1,774E-02
β_{city}	5,209E-05	2,132E-04	1,925E-04	-1,500E-04	1,148E-03	8,090E-04	2,612E-04	-1,455E-04	-1,204E-03	-9,742E-04	-2,603E-03	-2,661E-03
β_7	-2,583E-04	1,475E-04	7,528E-04	-2,449E-03	5,445E-03	3,821E-03	3,090E-04	4,120E-03	-1,944E-03	-6,707E-03	-5,214E-03	-1,353E-02
$\beta_{9,Hi}$	-4,781E-04	-5,832E-05	5,005E-04	-2,903E-04	1,572E-03	1,328E-03	7,814E-04	-2,198E-03	-5,509E-04	-5,150E-05	-1,993E-03	-4,520E-04
$\beta_{9,Na}$	-6,820E-04	-3,121E-04	1,782E-04	-9,292E-05	-2,258E-05	-2,124E-04	4,912E-04	-2,367E-03	4,891E-04	8,793E-04	1,159E-03	2,422E-03
$\beta_{1,m}$	-4,963E-03	2,278E-03	-5,591E-03	5,596E-03	-1,040E-01	-9,333E-02	-1,122E-02	1,851E-02	2,202E-02	1,530E-02	2,070E-02	4,282E-02
$\beta_{1,f}$	3,819E-03	6,220E-04	-1,017E-02	8,452E-03	-3,499E-02	-3,285E-03	-3,145E-03	-1,057E-02	1,026E-02	2,644E-02	3,967E-02	3,758E-02
$\beta_{2,m}$	2,441E-03	2,257E-02	3,019E-03	3,591E-03	-1,707E-01	-4,316E-01	-1,336E-02	-2,986E-02	7,742E-03	1,894E-02	9,492E-03	2,479E-02
$\beta_{2,f}$	-3,956E-03	4,747E-03	3,793E-03	2,606E-03	-3,569E-02	-1,664E-02	-3,591E-03	-1,377E-03	9,828E-03	1,520E-02	4,181E-02	4,743E-02
α_0	-1,152E-02	9,203E-03	5,731E-03	-4,463E-04	-2,031E-03	5,821E-03	1,579E-03	1,272E-02	1,262E-02	1,161E-02	2,196E-02	1,825E-02
α_3	2,452E-03	-8,532E-04	-2,097E-03	3,310E-03	-7,383E-03	-4,679E-03	-4,741E-04	-7,482E-03	9,743E-04	7,536E-03	9,542E-04	1,051E-02
α_5	-9,822E-03	7,495E-03	2,257E-02	-9,338E-03	4,318E-02	-2,238E-03	4,058E-03	1,148E-02	-8,848E-04	-1,623E-02	1,627E-02	4,044E-03
α_{10}	3,910E-02	-1,091E-02	2,836E-03	9,063E-04	2,878E-03	-5,449E-03	-1,373E-03	-1,822E-02	-1,430E-02	-1,126E-02	1,095E-02	6,362E-03
$\delta_{1,m}$	-1,091E-02	2,925E-02	1,135E-03	-2,848E-03	-6,898E-03	-1,381E-02	3,598E-03	3,301E-02	3,188E-02	2,971E-02	8,810E-04	-4,286E-04
$\delta_{1,f}$	2,836E-03	1,135E-03	2,581E-02	-2,887E-03	1,504E-02	-3,256E-02	8,007E-04	-1,434E-03	-2,601E-03	-3,450E-03	2,144E-02	1,510E-02
δ_2	9,063E-04	-2,848E-03	-2,887E-03	6,075E-03	-1,384E-02	-7,172E-03	-1,311E-03	-7,474E-03	4,726E-04	7,106E-03	6,215E-03	1,708E-02
δ_3	2,878E-03	-6,898E-03	1,504E-02	-1,384E-02	2,289E-01	2,289E-01	1,660E-02	-1,614E-02	-2,236E-02	-1,902E-02	-2,089E-02	-3,752E-02
δ_4	-5,449E-03	-1,381E-02	-3,256E-02	-7,172E-03	2,289E-01	1,453E+00	7,821E-03	4,405E-02	2,675E-02	2,091E-02	1,499E-02	-1,348E-02
δ_5	-1,373E-03	3,598E-03	8,007E-04	-1,311E-03	1,660E-02	7,821E-03	1,315E-02	3,583E-03	4,255E-03	5,342E-03	-9,831E-04	-1,314E-03
$\delta_{0,m,1}$	-1,822E-02	3,301E-02	-1,434E-03	-7,474E-03	-1,614E-02	4,405E-02	3,583E-03	1,091E-01	4,670E-02	2,496E-02	-5,985E-03	-2,705E-02
$\delta_{0,m,2}$	-1,430E-02	3,188E-02	-2,601E-03	4,726E-04	-2,236E-02	2,675E-02	4,255E-03	4,670E-02	1,093E-01	4,618E-02	9,229E-03	1,560E-02
$\delta_{0,m,3}$	-1,126E-02	2,971E-02	-3,450E-03	7,106E-03	-1,902E-02	2,091E-02	5,342E-03	2,496E-02	4,618E-02	7,554E-02	2,054E-02	4,861E-02
$\delta_{0,f,2}$	1,095E-02	8,810E-04	2,144E-02	6,215E-03	-2,089E-02	1,499E-02	-9,831E-04	-5,985E-03	9,229E-03	2,054E-02	3,677E-01	1,094E-01
$\delta_{0,f,3}$	6,362E-03	-4,286E-04	1,510E-02	1,708E-02	-3,752E-02	-1,348E-02	-1,314E-03	-2,705E-02	1,560E-02	4,861E-02	1,094E-01	2,565E-01

Table S3.6: Covariance matrix for MM lung cancer model.

	$\beta_{0,m}$	$\beta_{0,f}$	β_{city}	β_7	$\beta_{9,Hi}$	$\beta_{9,Na}$	$\beta_{1,m}$	$\beta_{1,f}$	$\beta_{2,m}$	$\beta_{2,f}$	α_0	α_3	α_5
$\beta_{0,m}$	1,349E-02	8,569E-04	-1,153E-03	3,505E-08	-1,201E-03	-1,441E-04	-2,711E-03	-7,313E-04	-2,707E-02	-3,036E-03	-1,529E-03	3,234E-04	-5,516E-03
$\beta_{0,f}$	8,569E-04	5,088E-03	-1,040E-03	2,613E-04	-1,255E-03	-1,944E-04	-1,108E-03	-3,960E-03	-1,729E-03	-2,681E-02	-3,704E-03	1,495E-03	-1,518E-02
β_{city}	-1,153E-03	-1,040E-03	3,550E-03	2,087E-04	1,135E-03	-2,406E-03	-1,017E-03	-6,637E-04	-2,622E-04	-4,055E-04	2,844E-04	3,017E-04	1,925E-05
β_7	3,505E-08	2,613E-04	2,087E-04	1,781E-03	5,504E-05	-9,256E-05	-6,084E-03	-7,345E-03	-3,856E-03	-3,355E-03	3,903E-04	-1,626E-03	7,008E-03
$\beta_{9,Hi}$	-1,201E-03	-1,255E-03	1,135E-03	5,504E-05	4,593E-03	3,438E-04	-1,431E-03	-4,510E-04	5,281E-04	1,176E-04	1,914E-03	-5,570E-05	3,211E-03
$\beta_{9,Na}$	-1,441E-04	-1,944E-04	-2,406E-03	-9,256E-05	3,438E-04	1,250E-02	-2,586E-04	6,817E-04	2,457E-03	1,098E-03	1,641E-03	-5,778E-04	3,083E-03
$\beta_{1,m}$	-2,711E-03	-1,108E-03	-1,017E-03	-6,084E-03	-1,431E-03	-2,586E-04	1,266E-01	3,527E-02	1,493E-01	2,460E-02	-1,621E-03	7,374E-03	-5,504E-02
$\beta_{1,f}$	-7,313E-04	-3,960E-03	-6,637E-04	-7,345E-03	-4,510E-04	6,817E-04	3,527E-02	1,050E-01	1,675E-02	8,884E-02	-8,700E-04	7,267E-03	-1,089E-01
$\beta_{2,m}$	-2,707E-02	-1,729E-03	-2,622E-04	-3,856E-03	5,281E-04	2,457E-03	1,493E-01	1,675E-02	1,065E+00	4,789E-02	2,306E-03	5,053E-03	5,221E-02
$\beta_{2,f}$	-3,036E-03	-2,681E-02	-4,055E-04	-3,355E-03	1,176E-04	1,098E-03	2,460E-02	8,884E-02	4,789E-02	7,792E-01	9,912E-03	-4,986E-03	2,209E-01
α_0	-1,529E-03	-3,704E-03	2,844E-04	3,903E-04	1,914E-03	1,641E-03	-1,621E-03	-8,700E-04	2,306E-03	9,912E-03	2,238E-02	-1,193E-02	8,872E-02
α_3	3,234E-04	1,495E-03	3,017E-04	-1,626E-03	-5,570E-05	-5,778E-04	7,374E-03	7,267E-03	5,053E-03	-4,986E-03	-1,193E-02	2,289E-02	-1,020E-01
α_5	-5,516E-03	-1,518E-02	1,925E-05	7,008E-03	3,211E-03	3,083E-03	-5,504E-02	-1,089E-01	5,221E-02	2,209E-01	8,872E-02	-1,020E-01	1,312E+00
α_{10}	1,498E-03	-1,014E-03	1,159E-04	-1,925E-04	-5,246E-04	-7,850E-04	-2,381E-03	3,422E-03	2,399E-03	-1,770E-03	-1,239E-04	1,982E-03	-8,821E-03
$\delta_{1,m}$	-1,663E-02	-1,650E-04	2,135E-04	1,251E-04	-1,984E-04	-4,624E-04	1,596E-03	7,445E-04	2,238E-02	3,374E-03	-1,866E-04	5,433E-04	-2,748E-04
$\delta_{1,f}$	6,036E-04	-3,865E-03	1,745E-04	7,435E-04	2,712E-04	-3,549E-06	-4,579E-03	-9,315E-03	1,808E-03	4,331E-04	-1,061E-03	-6,705E-04	-1,415E-03
δ_2	7,996E-04	-2,985E-04	-1,427E-04	-2,171E-03	-2,861E-04	-9,175E-05	4,969E-03	8,089E-03	3,489E-03	2,560E-03	-1,063E-04	5,983E-04	-3,571E-03
δ_3	5,975E-03	2,042E-03	1,073E-03	5,079E-03	1,435E-03	-3,853E-05	-1,001E-01	-3,204E-02	-1,682E-01	-4,389E-02	-4,237E-06	-3,412E-03	4,616E-03
δ_4	-8,906E-03	-3,023E-03	7,661E-04	3,594E-03	1,120E-03	-3,658E-04	-9,165E-02	-4,067E-03	-4,246E-01	-1,627E-02	-3,447E-03	-1,387E-03	-8,046E-03
δ_5	-3,130E-03	-1,794E-04	2,498E-04	2,936E-04	7,555E-04	4,809E-04	-1,081E-02	-2,857E-03	-1,296E-02	-4,328E-03	3,072E-04	-2,745E-04	5,853E-04
$\delta_{0,m,1}$	-2,111E-02	6,934E-04	-1,635E-04	3,697E-03	-2,307E-03	-2,480E-03	1,816E-02	-1,080E-02	-3,342E-02	9,268E-05	4,053E-04	-2,695E-03	6,252E-03
$\delta_{0,m,2}$	-2,192E-02	-5,836E-04	-1,151E-03	-1,860E-03	-6,897E-04	2,810E-04	2,163E-02	9,894E-03	6,584E-03	1,087E-02	2,741E-04	6,041E-04	2,678E-03
$\delta_{0,m,3}$	-2,075E-02	-1,396E-03	-8,861E-04	-6,050E-03	-1,845E-04	6,548E-04	1,364E-02	2,565E-02	1,982E-02	1,410E-02	-1,421E-04	2,742E-03	-1,045E-02
$\delta_{0,f,2}$	-9,484E-04	-2,855E-02	-3,514E-03	-7,439E-03	-2,784E-03	1,554E-03	2,929E-02	5,916E-02	1,655E-02	6,817E-02	9,796E-03	-1,008E-02	8,994E-02
$\delta_{0,f,3}$	-6,657E-04	-1,857E-02	-2,916E-03	-1,438E-02	-5,993E-04	2,606E-03	4,718E-02	4,375E-02	2,694E-02	6,044E-02	5,855E-03	-6,947E-03	4,102E-02

Table S3.6: Covariance matrix for MM lung cancer model. (cont'd)

	α_{10}	$\delta_{1,m}$	$\delta_{1,f}$	δ_2	δ_3	δ_4	δ_5	$\delta_{0,m,1}$	$\delta_{0,m,2}$	$\delta_{0,m,3}$	$\delta_{0,f,2}$	$\delta_{0,f,3}$
$\beta_{0,m}$	1,498E-03	-1,663E-02	6,036E-04	7,996E-04	5,975E-03	-8,906E-03	-3,130E-03	-2,111E-02	-2,192E-02	-2,075E-02	-9,484E-04	-6,657E-04
$\beta_{0,f}$	-1,014E-03	-1,650E-04	-3,865E-03	-2,985E-04	2,042E-03	-3,023E-03	-1,794E-04	6,934E-04	-5,836E-04	-1,396E-03	-2,855E-02	-1,857E-02
β_{city}	1,159E-04	2,135E-04	1,745E-04	-1,427E-04	1,073E-03	7,661E-04	2,498E-04	-1,635E-04	-1,151E-03	-8,861E-04	-3,514E-03	-2,916E-03
β_7	-1,925E-04	1,251E-04	7,435E-04	-2,171E-03	5,079E-03	3,594E-03	2,936E-04	3,697E-03	-1,860E-03	-6,050E-03	-7,439E-03	-1,438E-02
$\beta_{9,Hi}$	-5,246E-04	-1,984E-04	2,712E-04	-2,861E-04	1,435E-03	1,120E-03	7,555E-04	-2,307E-03	-6,897E-04	-1,845E-04	-2,784E-03	-5,993E-04
$\beta_{9,Na}$	-7,850E-04	-4,624E-04	-3,549E-06	-9,175E-05	-3,853E-05	-3,658E-04	4,809E-04	-2,480E-03	2,810E-04	6,548E-04	1,554E-03	2,606E-03
$\beta_{1,m}$	-2,381E-03	1,596E-03	-4,579E-03	4,969E-03	-1,001E-01	-9,165E-02	-1,081E-02	1,816E-02	2,163E-02	1,364E-02	2,929E-02	4,718E-02
$\beta_{1,f}$	3,422E-03	7,445E-04	-9,315E-03	8,089E-03	-3,204E-02	-4,067E-03	-2,857E-03	-1,080E-02	9,894E-03	2,565E-02	5,916E-02	4,375E-02
$\beta_{2,m}$	2,399E-03	2,238E-02	1,808E-03	3,489E-03	-1,682E-01	-4,246E-01	-1,296E-02	-3,342E-02	6,584E-03	1,982E-02	1,655E-02	2,694E-02
$\beta_{2,f}$	-1,770E-03	3,374E-03	4,331E-04	2,560E-03	-4,389E-02	-1,627E-02	-4,328E-03	9,268E-05	1,087E-02	1,410E-02	6,817E-02	6,044E-02
α_0	-1,239E-04	-1,866E-04	-1,061E-03	-1,063E-04	-4,237E-06	-3,447E-03	3,072E-04	4,053E-04	2,741E-04	-1,421E-04	9,796E-03	5,855E-03
α_3	1,982E-03	5,433E-04	-6,705E-04	5,983E-04	-3,412E-03	-1,387E-03	-2,745E-04	-2,695E-03	6,041E-04	2,742E-03	-1,008E-02	-6,947E-03
α_5	-8,821E-03	-2,748E-04	-1,415E-03	-3,571E-03	4,616E-03	-8,046E-03	5,853E-04	6,252E-03	2,678E-03	-1,045E-02	8,994E-02	4,102E-02
α_{10}	1,870E-02	1,798E-04	-1,093E-03	3,129E-04	-8,982E-04	-2,321E-04	-1,776E-04	-2,618E-03	-1,377E-05	1,213E-03	5,904E-04	-1,600E-03
$\delta_{1,m}$	1,798E-04	2,682E-02	1,033E-03	-2,671E-03	-6,284E-03	-1,365E-02	3,311E-03	2,951E-02	2,943E-02	2,711E-02	5,381E-04	-1,434E-03
$\delta_{1,f}$	-1,093E-03	1,033E-03	2,450E-02	-2,852E-03	1,420E-02	-3,163E-02	6,766E-04	-1,112E-03	-2,774E-03	-3,651E-03	2,594E-02	1,492E-02
δ_2	3,129E-04	-2,671E-03	-2,852E-03	5,686E-03	-1,325E-02	-6,942E-03	-1,253E-03	-6,732E-03	4,864E-04	6,423E-03	9,459E-03	1,882E-02
δ_3	-8,982E-04	-6,284E-03	1,420E-02	-1,325E-02	2,226E-01	2,276E-01	1,592E-02	-1,534E-02	-2,214E-02	-1,786E-02	-3,247E-02	-4,269E-02
δ_4	-2,321E-04	-1,365E-02	-3,163E-02	-6,942E-03	2,276E-01	1,400E+00	8,104E-03	4,304E-02	2,633E-02	1,991E-02	2,141E-02	-1,466E-02
δ_5	-1,776E-04	3,311E-03	6,766E-04	-1,253E-03	1,592E-02	8,104E-03	1,273E-02	3,276E-03	3,968E-03	4,998E-03	-1,612E-03	-1,559E-03
$\delta_{0,m,1}$	-2,618E-03	2,951E-02	-1,112E-03	-6,732E-03	-1,534E-02	4,304E-02	3,276E-03	1,035E-01	4,382E-02	2,259E-02	-9,147E-03	-2,918E-02
$\delta_{0,m,2}$	-1,377E-05	2,943E-02	-2,774E-03	4,864E-04	-2,214E-02	2,633E-02	3,968E-03	4,382E-02	1,086E-01	4,328E-02	1,397E-02	1,748E-02
$\delta_{0,m,3}$	1,213E-03	2,711E-02	-3,651E-03	6,423E-03	-1,786E-02	1,991E-02	4,998E-03	2,259E-02	4,328E-02	6,978E-02	3,002E-02	5,251E-02
$\delta_{0,f,2}$	5,904E-04	5,381E-04	2,594E-02	9,459E-03	-3,247E-02	2,141E-02	-1,612E-03	-9,147E-03	1,397E-02	3,002E-02	8,123E-01	1,870E-01
$\delta_{0,f,3}$	-1,600E-03	-1,434E-03	1,492E-02	1,882E-02	-4,269E-02	-1,466E-02	-1,559E-03	-2,918E-02	1,748E-02	5,251E-02	1,870E-01	3,450E-01

Table S3.7: Covariance matrix for GAM lung cancer model.

	$\beta_{0,m}$	$\beta_{0,f}$	β_{city}	β_7	$\beta_{9,Hi}$	$\beta_{9,Na}$	$\beta_{1,m}$	$\beta_{1,f}$	$\beta_{2,m}$	$\beta_{2,f}$	α_0	α_3	α_5	α_8
$\beta_{0,m}$	1,501E-02	9,000E-04	-1,136E-03	9,004E-05	-1,214E-03	-1,677E-04	-2,992E-03	-1,237E-03	-2,691E-02	-2,851E-03	-4,567E-03	6,154E-04	-5,642E-03	9,924E-03
$\beta_{0,f}$	9,000E-04	5,534E-03	-1,017E-03	3,678E-04	-1,237E-03	-1,933E-04	-1,310E-03	-3,952E-03	-1,174E-03	-2,636E-02	-5,576E-03	1,118E-03	-1,565E-02	1,762E-02
β_{city}	-1,136E-03	-1,017E-03	3,557E-03	2,337E-04	1,132E-03	-2,416E-03	-1,166E-03	-7,616E-04	-4,174E-04	-3,274E-04	2,417E-04	1,034E-05	1,226E-03	-5,633E-04
β_7	9,004E-05	3,678E-04	2,337E-04	1,934E-03	5,468E-05	-1,218E-04	-6,389E-03	-7,580E-03	-3,791E-03	-3,247E-03	-2,907E-05	-2,859E-03	9,828E-03	8,110E-04
$\beta_{9,Hi}$	-1,214E-03	-1,237E-03	1,132E-03	5,468E-05	4,550E-03	2,980E-04	-1,604E-03	-4,843E-04	2,582E-04	-4,548E-06	1,850E-03	-1,530E-04	3,619E-03	-1,030E-02
$\beta_{9,Na}$	-1,677E-04	-1,933E-04	-2,416E-03	-1,218E-04	2,980E-04	1,246E-02	-2,718E-04	7,694E-04	2,410E-03	8,982E-04	1,556E-03	-3,066E-04	1,921E-03	-9,550E-03
$\beta_{1,m}$	-2,992E-03	-1,310E-03	-1,166E-03	-6,389E-03	-1,604E-03	-2,718E-04	1,291E-01	3,595E-02	1,546E-01	2,087E-02	-1,309E-04	9,966E-03	-6,804E-02	-1,460E-02
$\beta_{1,f}$	-1,237E-03	-3,952E-03	-7,616E-04	-7,580E-03	-4,843E-04	7,694E-04	3,595E-02	1,055E-01	1,940E-02	8,690E-02	3,002E-04	1,130E-02	-1,252E-01	-3,408E-04
$\beta_{2,m}$	-2,691E-02	-1,174E-03	-4,174E-04	-3,791E-03	2,582E-04	2,410E-03	1,546E-01	1,940E-02	1,069E+00	3,364E-02	-1,064E-03	7,346E-03	-5,577E-03	-6,861E-04
$\beta_{2,f}$	-2,851E-03	-2,636E-02	-3,274E-04	-3,247E-03	-4,548E-06	8,982E-04	2,087E-02	8,690E-02	3,364E-02	7,694E-01	1,091E-02	-3,424E-03	2,040E-01	-5,747E-02
α_0	-4,567E-03	-5,576E-03	2,417E-04	-2,907E-05	1,850E-03	1,556E-03	-1,309E-04	3,002E-04	-1,064E-03	1,091E-02	3,908E-02	-1,779E-02	1,242E-01	-2,349E-01
α_3	6,154E-04	1,118E-03	1,034E-05	-2,859E-03	-1,530E-04	-3,066E-04	9,966E-03	1,130E-02	7,346E-03	-3,424E-03	-1,779E-02	2,716E-02	-1,246E-01	5,526E-02
α_5	-5,642E-03	-1,565E-02	1,226E-03	9,828E-03	3,619E-03	1,921E-03	-6,804E-02	-1,252E-01	-5,577E-03	2,040E-01	1,242E-01	-1,246E-01	1,411E+00	-6,804E-01
α_8	9,924E-03	1,762E-02	-5,633E-04	8,110E-04	-1,030E-02	-9,550E-03	-1,460E-02	-3,408E-04	-6,861E-04	-5,747E-02	-2,349E-01	5,526E-02	-6,804E-01	6,875E+00
α_9	-1,179E-02	-2,313E-02	7,627E-04	-1,893E-03	1,916E-02	1,851E-02	3,135E-02	3,916E-03	4,543E-02	1,244E-01	2,600E-01	-2,619E-02	8,973E-01	-1,354E+01
α_{10}	7,438E-03	-1,456E-03	1,000E-04	-8,888E-05	-3,595E-04	-5,357E-04	-1,881E-03	1,022E-03	7,205E-04	-3,152E-04	-9,244E-03	3,982E-03	-1,584E-02	5,266E-02
$\delta_{1,m}$	-1,843E-02	-3,038E-04	2,042E-04	8,205E-05	-6,620E-05	-3,134E-04	1,884E-03	9,096E-04	2,319E-02	3,614E-03	4,632E-03	-8,612E-04	5,340E-03	1,301E-03
$\delta_{1,f}$	5,902E-04	-5,143E-03	1,817E-04	7,229E-04	4,260E-04	1,154E-04	-5,225E-03	-1,044E-02	2,788E-03	1,881E-03	5,894E-03	-2,686E-03	2,153E-02	-2,459E-02
δ_2	8,097E-04	-3,884E-04	-1,537E-04	-2,421E-03	-3,020E-04	-9,798E-05	5,633E-03	8,609E-03	3,282E-03	2,610E-03	-1,610E-05	3,095E-03	-9,574E-03	-4,177E-03
δ_3	7,038E-03	2,034E-03	1,190E-03	5,465E-03	1,557E-03	-6,288E-05	-1,072E-01	-3,438E-02	-1,739E-01	-3,533E-02	-2,668E-03	-6,453E-03	3,665E-02	1,111E-02
δ_4	-1,397E-02	-4,045E-03	7,841E-04	3,917E-03	1,363E-03	-2,656E-04	-9,049E-02	-3,346E-03	-4,723E-01	-1,193E-02	1,283E-02	-8,824E-03	2,031E-02	3,810E-02
δ_5	-3,239E-03	-1,880E-04	2,659E-04	3,157E-04	7,915E-04	5,003E-04	-1,163E-02	-2,999E-03	-1,387E-02	-3,700E-03	1,028E-03	-5,594E-04	3,595E-03	2,400E-03
$\delta_{0,m,1}$	-2,392E-02	8,158E-04	-1,639E-04	3,998E-03	-2,265E-03	-2,441E-03	1,859E-02	-1,058E-02	-3,196E-02	-7,234E-04	5,841E-03	-6,924E-03	1,345E-02	-7,185E-03
$\delta_{0,m,2}$	-2,388E-02	-6,695E-04	-1,193E-03	-2,053E-03	-5,323E-04	4,870E-04	2,205E-02	1,073E-02	6,839E-03	9,046E-03	5,799E-03	1,650E-03	-5,608E-03	-5,952E-03
$\delta_{0,m,3}$	-2,265E-02	-1,704E-03	-9,480E-04	-6,544E-03	-2,757E-06	9,028E-04	1,492E-02	2,623E-02	1,824E-02	1,383E-02	5,575E-03	7,782E-03	-2,199E-02	-2,581E-03
$\delta_{0,f,1}$	-6,679E-04	-2,084E-02	-2,499E-03	-5,153E-03	-1,947E-03	1,084E-03	2,060E-02	3,861E-02	8,687E-03	3,896E-02	1,647E-02	6,303E-04	1,206E-02	-1,348E-02
$\delta_{0,f,2}$	-1,125E-03	-1,694E-02	-2,541E-03	-1,283E-02	-4,584E-04	2,292E-03	4,114E-02	3,576E-02	2,263E-02	4,343E-02	1,361E-02	8,861E-03	3,740E-03	-5,271E-03

Table S3.7: Covariance matrix for GAM lung cancer model. (cont'd)

	α_9	α_{10}	$\delta_{1,m}$	$\delta_{1,f}$	δ_2	δ_3	δ_4	δ_5	$\delta_{0,m,1}$	$\delta_{0,m,2}$	$\delta_{0,m,3}$	$\delta_{0,f,1}$	$\delta_{0,f,2}$
$\beta_{0,m}$	-1,179E-02	7,438E-03	-1,843E-02	5,902E-04	8,097E-04	7,038E-03	-1,397E-02	-3,239E-03	-2,392E-02	-2,388E-02	-2,265E-02	-6,679E-04	-1,125E-03
$\beta_{0,f}$	-2,313E-02	-1,456E-03	-3,038E-04	-5,143E-03	-3,884E-04	2,034E-03	-4,045E-03	-1,880E-04	8,158E-04	-6,695E-04	-1,704E-03	-2,084E-02	-1,694E-02
β_{city}	7,627E-04	1,000E-04	2,042E-04	1,817E-04	-1,537E-04	1,190E-03	7,841E-04	2,659E-04	-1,639E-04	-1,193E-03	-9,480E-04	-2,499E-03	-2,541E-03
β_7	-1,893E-03	-8,888E-05	8,205E-05	7,229E-04	-2,421E-03	5,465E-03	3,917E-03	3,157E-04	3,998E-03	-2,053E-03	-6,544E-03	-5,153E-03	-1,283E-02
$\beta_{9,Hi}$	1,916E-02	-3,595E-04	-6,620E-05	4,260E-04	-3,020E-04	1,557E-03	1,363E-03	7,915E-04	-2,265E-03	-5,323E-04	-2,757E-06	-1,947E-03	-4,584E-04
$\beta_{9,Na}$	1,851E-02	-5,357E-04	-3,134E-04	1,154E-04	-9,798E-05	-6,288E-05	-2,656E-04	5,003E-04	-2,441E-03	4,870E-04	9,028E-04	1,084E-03	2,292E-03
$\beta_{1,m}$	3,135E-02	-1,881E-03	1,884E-03	-5,225E-03	5,633E-03	-1,072E-01	-9,049E-02	-1,163E-02	1,859E-02	2,205E-02	1,492E-02	2,060E-02	4,114E-02
$\beta_{1,f}$	3,916E-03	1,022E-03	9,096E-04	-1,044E-02	8,609E-03	-3,438E-02	-3,346E-03	-2,999E-03	-1,058E-02	1,073E-02	2,623E-02	3,861E-02	3,576E-02
$\beta_{2,m}$	4,543E-02	7,205E-04	2,319E-02	2,788E-03	3,282E-03	-1,739E-01	-4,723E-01	-1,387E-02	-3,196E-02	6,839E-03	1,824E-02	8,687E-03	2,263E-02
$\beta_{2,f}$	1,244E-01	-3,152E-04	3,614E-03	1,881E-03	2,610E-03	-3,533E-02	-1,193E-02	-3,700E-03	-7,234E-04	9,046E-03	1,383E-02	3,896E-02	4,343E-02
α_0	2,600E-01	-9,244E-03	4,632E-03	5,894E-03	-1,610E-05	-2,668E-03	1,283E-02	1,028E-03	5,841E-03	5,799E-03	5,575E-03	1,647E-02	1,361E-02
α_3	-2,619E-02	3,982E-03	-8,612E-04	-2,686E-03	3,095E-03	-6,453E-03	-8,824E-03	-5,594E-04	-6,924E-03	1,650E-03	7,782E-03	6,303E-04	8,861E-03
α_5	8,973E-01	-1,584E-02	5,340E-03	2,153E-02	-9,574E-03	3,665E-02	2,031E-02	3,595E-03	1,345E-02	-5,608E-03	-2,199E-02	1,206E-02	3,740E-03
α_8	-1,354E+01	5,266E-02	1,301E-03	-2,459E-02	-4,177E-03	1,111E-02	3,810E-02	2,400E-03	-7,185E-03	-5,952E-03	-2,581E-03	-1,348E-02	-5,271E-03
α_9	3,021E+01	1,125E-03	-1,476E-02	5,275E-03	6,997E-03	3,736E-03	-2,654E-01	-1,039E-02	2,036E-03	9,482E-03	4,554E-03	7,331E-03	-1,029E-02
$\delta_{1,f}$	1,125E-03	2,601E-02	-8,889E-03	2,747E-04	6,950E-04	2,844E-03	-1,433E-02	-1,464E-03	-1,393E-02	-1,159E-02	-9,775E-03	7,038E-03	3,797E-03
$\delta_{1,m}$	-1,476E-02	-8,889E-03	2,874E-02	1,156E-03	-2,754E-03	-7,263E-03	-1,371E-02	3,421E-03	3,261E-02	3,112E-02	2,868E-02	8,507E-04	-9,378E-05
$\delta_{1,f}$	5,275E-03	2,747E-04	1,156E-03	2,951E-02	-2,843E-03	1,478E-02	-3,728E-02	8,330E-04	-1,658E-03	-2,918E-03	-3,617E-03	2,091E-02	1,490E-02
δ_2	6,997E-03	6,950E-04	-2,754E-03	-2,843E-03	6,130E-03	-1,425E-02	-6,403E-03	-1,357E-03	-7,358E-03	6,268E-04	6,952E-03	6,224E-03	1,626E-02
δ_3	3,736E-03	2,844E-03	-7,263E-03	1,478E-02	-1,425E-02	2,384E-01	2,206E-01	1,741E-02	-1,809E-02	-2,370E-02	-1,937E-02	-2,104E-02	-3,679E-02
δ_4	-2,654E-01	-1,433E-02	-1,371E-02	-3,728E-02	-6,403E-03	2,206E-01	1,701E+00	7,153E-03	5,925E-02	3,624E-02	2,637E-02	1,448E-02	-1,280E-02
δ_5	-1,039E-02	-1,464E-03	3,421E-03	8,330E-04	-1,357E-03	1,741E-02	7,153E-03	1,313E-02	3,314E-03	3,904E-03	4,944E-03	-1,124E-03	-1,443E-03
$\delta_{0,m,1}$	2,036E-03	-1,393E-02	3,261E-02	-1,658E-03	-7,358E-03	-1,809E-02	5,925E-02	3,314E-03	1,113E-01	4,682E-02	2,478E-02	-5,992E-03	-2,544E-02
$\delta_{0,m,2}$	9,482E-03	-1,159E-02	3,112E-02	-2,918E-03	6,268E-04	-2,370E-02	3,624E-02	3,904E-03	4,682E-02	1,066E-01	4,487E-02	8,934E-03	1,528E-02
$\delta_{0,m,3}$	4,554E-03	-9,775E-03	2,868E-02	-3,617E-03	6,952E-03	-1,937E-02	2,637E-02	4,944E-03	2,478E-02	4,487E-02	7,125E-02	1,951E-02	4,515E-02
$\delta_{0,f,1}$	7,331E-03	7,038E-03	8,507E-04	2,091E-02	6,224E-03	-2,104E-02	1,448E-02	-1,124E-03	-5,992E-03	8,934E-03	1,951E-02	3,457E-01	1,017E-01
$\delta_{0,f,2}$	-1,029E-02	3,797E-03	-9,378E-05	1,490E-02	1,626E-02	-3,679E-02	-1,280E-02	-1,443E-03	-2,544E-02	1,528E-02	4,515E-02	1,017E-01	2,346E-01

Table S3.8: Covariance matrix for GMM lung cancer model.

	$\beta_{0,m}$	$\beta_{0,f}$	β_{city}	β_7	$\beta_{9,Hi}$	$\beta_{9,Na}$	$\beta_{1,m}$	$\beta_{1,f}$	$\beta_{2,m}$	$\beta_{2,f}$	α_0	α_3	α_5	α_8
$\beta_{0,m}$	1,465E-02	9,380E-04	-1,177E-03	-2,551E-05	-1,245E-03	-1,599E-04	-2,914E-03	-5,985E-04	-2,649E-02	-3,276E-03	-2,188E-03	8,394E-04	-8,425E-03	-4,828E-04
$\beta_{0,f}$	9,380E-04	5,145E-03	-1,059E-03	2,531E-04	-1,258E-03	-1,721E-04	-1,132E-03	-3,887E-03	-1,073E-03	-2,639E-02	-4,223E-03	1,881E-03	-1,717E-02	2,130E-02
β_{city}	-1,177E-03	-1,059E-03	3,552E-03	2,196E-04	1,140E-03	-2,402E-03	-1,065E-03	-7,108E-04	-3,449E-04	-3,564E-04	4,506E-04	1,134E-04	1,040E-03	-2,814E-03
β_7	-2,551E-05	2,531E-04	2,196E-04	1,804E-03	6,204E-05	-9,882E-05	-6,114E-03	-7,453E-03	-3,920E-03	-3,305E-03	4,448E-04	-1,612E-03	7,492E-03	-3,743E-04
$\beta_{9,Hi}$	-1,245E-03	-1,258E-03	1,140E-03	6,204E-05	4,574E-03	3,166E-04	-1,504E-03	-4,342E-04	4,852E-04	6,863E-05	1,805E-03	-1,298E-04	3,590E-03	-1,256E-02
$\beta_{9,Na}$	-1,599E-04	-1,721E-04	-2,402E-03	-9,882E-05	3,166E-04	1,246E-02	-3,033E-04	7,585E-04	2,509E-03	1,010E-03	1,306E-03	-4,073E-04	2,220E-03	-9,223E-03
$\beta_{1,m}$	-2,914E-03	-1,132E-03	-1,065E-03	-6,114E-03	-1,504E-03	-3,033E-04	1,287E-01	3,525E-02	1,521E-01	2,497E-02	-1,673E-03	7,816E-03	-5,490E-02	-3,710E-03
$\beta_{1,f}$	-5,985E-04	-3,887E-03	-7,108E-04	-7,453E-03	-4,342E-04	7,585E-04	3,525E-02	1,053E-01	1,767E-02	8,986E-02	-1,847E-03	7,507E-03	-1,096E-01	1,152E-02
$\beta_{2,m}$	-2,649E-02	-1,073E-03	-3,449E-04	-3,920E-03	4,852E-04	2,509E-03	1,521E-01	1,767E-02	1,066E+00	4,213E-02	-1,571E-03	6,646E-03	2,735E-02	-4,942E-03
$\beta_{2,f}$	-3,276E-03	-2,639E-02	-3,564E-04	-3,305E-03	6,863E-05	1,010E-03	2,497E-02	8,986E-02	4,213E-02	7,686E-01	1,026E-02	-6,008E-03	2,067E-01	-1,023E-01
α_0	-2,188E-03	-4,223E-03	4,506E-04	4,448E-04	1,805E-03	1,306E-03	-1,673E-03	-1,847E-03	-1,571E-03	1,026E-02	2,959E-02	-1,495E-02	1,179E-01	-2,171E-01
α_3	8,394E-04	1,881E-03	1,134E-04	-1,612E-03	-1,298E-04	-4,073E-04	7,816E-03	7,507E-03	6,646E-03	-6,008E-03	-1,495E-02	2,361E-02	-1,146E-01	4,089E-02
α_5	-8,425E-03	-1,717E-02	1,040E-03	7,492E-03	3,590E-03	2,220E-03	-5,490E-02	-1,096E-01	2,735E-02	2,067E-01	1,179E-01	-1,146E-01	1,386E+00	-8,134E-01
α_8	-4,828E-04	2,130E-02	-2,814E-03	-3,743E-04	-1,256E-02	-9,223E-03	-3,710E-03	1,152E-02	-4,942E-03	-1,023E-01	-2,171E-01	4,089E-02	-8,134E-01	7,094E+00
α_9	8,647E-03	-2,490E-02	3,214E-03	-1,221E-03	2,431E-02	2,079E-02	1,070E-02	5,292E-03	6,477E-02	1,805E-01	2,580E-01	-7,095E-03	1,008E+00	-1,422E+01
$\delta_{1,f}$	2,739E-03	-3,944E-04	7,123E-05	-2,115E-04	-3,598E-04	-5,267E-04	-1,690E-03	3,093E-03	3,252E-03	-3,326E-03	-5,244E-03	4,713E-03	-2,687E-02	2,640E-02
$\delta_{1,m}$	-1,781E-02	-3,230E-04	2,409E-04	1,760E-04	-6,202E-05	-3,464E-04	1,867E-03	2,696E-04	2,120E-02	3,600E-03	1,432E-03	-6,442E-04	6,047E-03	2,142E-02
$\delta_{1,f}$	5,332E-04	-4,422E-03	2,297E-04	8,276E-04	3,153E-04	-2,374E-05	-4,873E-03	-1,017E-02	-3,915E-04	9,147E-04	4,089E-03	-3,228E-03	1,534E-02	-4,269E-02
δ_2	8,123E-04	-2,802E-04	-1,481E-04	-2,202E-03	-3,157E-04	-1,208E-04	4,996E-03	8,267E-03	3,586E-03	2,669E-03	-1,269E-04	8,121E-04	-4,346E-03	-9,548E-03
δ_3	6,454E-03	2,089E-03	1,070E-03	5,086E-03	1,356E-03	-8,842E-05	-1,025E-01	-3,161E-02	-1,679E-01	-4,225E-02	-2,332E-03	-2,885E-03	1,863E-03	3,744E-02
δ_4	-1,117E-02	-4,339E-03	8,642E-04	3,780E-03	1,387E-03	-1,895E-04	-9,075E-02	-5,422E-03	-4,308E-01	-1,100E-02	8,913E-03	-7,104E-03	3,353E-02	-5,906E-02
δ_5	-3,036E-03	-1,849E-04	2,627E-04	3,314E-04	7,547E-04	4,717E-04	-1,102E-02	-3,011E-03	-1,307E-02	-4,106E-03	4,370E-04	-4,590E-04	1,896E-03	3,469E-03
$\delta_{0,m,1}$	-2,259E-02	5,269E-04	-1,112E-04	3,690E-03	-2,133E-03	-2,356E-03	1,856E-02	-1,115E-02	-3,307E-02	1,130E-03	1,474E-03	-3,082E-03	1,184E-02	1,900E-02
$\delta_{0,m,2}$	-2,299E-02	-5,423E-04	-1,093E-03	-1,773E-03	-5,751E-04	3,479E-04	2,168E-02	9,296E-03	5,799E-03	1,078E-02	4,520E-05	5,304E-04	2,339E-03	2,604E-02
$\delta_{0,m,3}$	-2,163E-02	-1,288E-03	-8,563E-04	-5,895E-03	-9,486E-05	7,282E-04	1,357E-02	2,489E-02	1,858E-02	1,372E-02	-6,921E-04	2,640E-03	-1,250E-02	3,155E-02
$\delta_{0,f,1}$	-1,166E-03	-2,611E-02	-3,200E-03	-6,909E-03	-2,623E-03	1,318E-03	2,715E-02	5,515E-02	1,275E-02	5,828E-02	8,027E-03	-8,783E-03	6,577E-02	3,255E-02
$\delta_{0,f,2}$	-8,568E-04	-1,744E-02	-2,700E-03	-1,377E-02	-6,252E-04	2,312E-03	4,454E-02	4,230E-02	2,507E-02	5,507E-02	4,479E-03	-4,812E-03	2,746E-02	4,308E-02

Table S3.8: Covariance matrix for GMM lung cancer model. (cont'd)

	α_9	α_{10}	$\delta_{1,m}$	$\delta_{1,f}$	δ_2	δ_3	δ_4	δ_5	$\delta_{0,m,1}$	$\delta_{0,m,2}$	$\delta_{0,m,3}$	$\delta_{0,f,1}$	$\delta_{0,f,2}$
$\beta_{0,m}$	8,647E-03	2,739E-03	-1,781E-02	5,332E-04	8,123E-04	6,454E-03	-1,117E-02	-3,036E-03	-2,259E-02	-2,299E-02	-2,163E-02	-1,166E-03	-8,568E-04
$\beta_{0,f}$	-2,490E-02	-3,944E-04	-3,230E-04	-4,422E-03	-2,802E-04	2,089E-03	-4,339E-03	-1,849E-04	5,269E-04	-5,423E-04	-1,288E-03	-2,611E-02	-1,744E-02
β_{city}	3,214E-03	7,123E-05	2,409E-04	2,297E-04	-1,481E-04	1,070E-03	8,642E-04	2,627E-04	-1,112E-04	-1,093E-03	-8,563E-04	-3,200E-03	-2,700E-03
β_7	-1,221E-03	-2,115E-04	1,760E-04	8,276E-04	-2,202E-03	5,086E-03	3,780E-03	3,314E-04	3,690E-03	-1,773E-03	-5,895E-03	-6,909E-03	-1,377E-02
$\beta_{9,Hi}$	2,431E-02	-3,598E-04	-6,202E-05	3,153E-04	-3,157E-04	1,356E-03	1,387E-03	7,547E-04	-2,133E-03	-5,751E-04	-9,486E-05	-2,623E-03	-6,252E-04
$\beta_{9,Na}$	2,079E-02	-5,267E-04	-3,464E-04	-2,374E-05	-1,208E-04	-8,842E-05	-1,895E-04	4,717E-04	-2,356E-03	3,479E-04	7,282E-04	1,318E-03	2,312E-03
$\beta_{1,m}$	1,070E-02	-1,690E-03	1,867E-03	-4,873E-03	4,996E-03	-1,025E-01	-9,075E-02	-1,102E-02	1,856E-02	2,168E-02	1,357E-02	2,715E-02	4,454E-02
$\beta_{1,f}$	5,292E-03	3,093E-03	2,696E-04	-1,017E-02	8,267E-03	-3,161E-02	-5,422E-03	-3,011E-03	-1,115E-02	9,296E-03	2,489E-02	5,515E-02	4,230E-02
$\beta_{2,m}$	6,477E-02	3,252E-03	2,120E-02	-3,915E-04	3,586E-03	-1,679E-01	-4,308E-01	-1,307E-02	-3,307E-02	5,799E-03	1,858E-02	1,275E-02	2,507E-02
$\beta_{2,f}$	1,805E-01	-3,326E-03	3,600E-03	9,147E-04	2,669E-03	-4,225E-02	-1,100E-02	-4,106E-03	1,130E-03	1,078E-02	1,372E-02	5,828E-02	5,507E-02
α_0	2,580E-01	-5,244E-03	1,432E-03	4,089E-03	-1,269E-04	-2,332E-03	8,913E-03	4,370E-04	1,474E-03	4,520E-05	-6,921E-04	8,027E-03	4,479E-03
α_3	-7,095E-03	4,713E-03	-6,442E-04	-3,228E-03	8,121E-04	-2,885E-03	-7,104E-03	-4,590E-04	-3,082E-03	5,304E-04	2,640E-03	-8,783E-03	-4,812E-03
α_5	1,008E+00	-2,687E-02	6,047E-03	1,534E-02	-4,346E-03	1,863E-03	3,353E-02	1,896E-03	1,184E-02	2,339E-03	-1,250E-02	6,577E-02	2,746E-02
α_8	-1,422E+01	2,640E-02	2,142E-02	-4,269E-02	-9,548E-03	3,744E-02	-5,906E-02	3,469E-03	1,900E-02	2,604E-02	3,155E-02	3,255E-02	4,308E-02
α_9	3,229E+01	2,679E-02	-5,840E-02	-7,210E-03	1,594E-02	-3,635E-02	-7,481E-02	-7,228E-03	-4,857E-02	-4,228E-02	-4,743E-02	-8,903E-02	-1,041E-01
$\delta_{1,f}$	2,679E-02	1,937E-02	-2,353E-03	-3,915E-03	3,950E-04	2,034E-03	-8,521E-03	-4,059E-04	-4,663E-03	-2,025E-03	-8,721E-04	-3,133E-04	-1,962E-03
$\delta_{1,m}$	-5,840E-02	-2,353E-03	2,748E-02	1,093E-03	-2,518E-03	-6,624E-03	-1,094E-02	3,132E-03	3,070E-02	3,001E-02	2,748E-02	1,217E-03	-5,970E-04
$\delta_{1,f}$	-7,210E-03	-3,915E-03	1,093E-03	2,878E-02	-2,689E-03	1,324E-02	-2,458E-02	7,893E-04	-1,044E-03	-3,301E-03	-4,285E-03	2,483E-02	1,481E-02
δ_2	1,594E-02	3,950E-04	-2,518E-03	-2,689E-03	5,628E-03	-1,326E-02	-6,401E-03	-1,271E-03	-6,607E-03	4,685E-04	6,270E-03	8,528E-03	1,762E-02
δ_3	-3,635E-02	2,034E-03	-6,624E-03	1,324E-02	-1,326E-02	2,222E-01	2,217E-01	1,612E-02	-1,619E-02	-2,190E-02	-1,725E-02	-2,907E-02	-4,019E-02
δ_4	-7,481E-02	-8,521E-03	-1,094E-02	-2,458E-02	-6,401E-03	2,217E-01	1,384E+00	7,409E-03	4,618E-02	2,773E-02	2,041E-02	2,205E-02	-1,201E-02
δ_5	-7,228E-03	-4,059E-04	3,132E-03	7,893E-04	-1,271E-03	1,612E-02	7,409E-03	1,159E-02	3,029E-03	3,562E-03	4,513E-03	-1,535E-03	-1,680E-03
$\delta_{0,m,1}$	-4,857E-02	-4,663E-03	3,070E-02	-1,044E-03	-6,607E-03	-1,619E-02	4,618E-02	3,029E-03	1,016E-01	4,450E-02	2,381E-02	-7,626E-03	-2,684E-02
$\delta_{0,m,2}$	-4,228E-02	-2,025E-03	3,001E-02	-3,301E-03	4,685E-04	-2,190E-02	2,773E-02	3,562E-03	4,450E-02	1,039E-01	4,288E-02	1,274E-02	1,620E-02
$\delta_{0,m,3}$	-4,743E-02	-8,721E-04	2,748E-02	-4,285E-03	6,270E-03	-1,725E-02	2,041E-02	4,513E-03	2,381E-02	4,288E-02	6,747E-02	2,716E-02	4,860E-02
$\delta_{0,f,1}$	-8,903E-02	-3,133E-04	1,217E-03	2,483E-02	8,528E-03	-2,907E-02	2,205E-02	-1,535E-03	-7,626E-03	1,274E-02	2,716E-02	6,764E-01	1,604E-01
$\delta_{0,f,2}$	-1,041E-01	-1,962E-03	-5,970E-04	1,481E-02	1,762E-02	-4,019E-02	-1,201E-02	-1,680E-03	-2,684E-02	1,620E-02	4,860E-02	1,604E-01	3,029E-01

S4. Female breast cancer, group BREAST (ICD10: C50)

Model description

The pooled study of Preston et al. (2002) includes not only the LSS, but also several other studies of radiation-induced breast cancer from Western populations. Therefore, it was decided to use the pooled study for ProZES. Radiation risk is given by the following EAR-model (EAR per 10^4 PY):

$$EAR(a,e,d) = \beta d \exp\left(\frac{\theta}{10}(e-25) + \gamma_1 \ln\frac{a}{50} + \gamma_2 \max\left(0, \ln\frac{a}{50}\right)\right) \tag{S4.1}$$

where parameters of Eq. (S4.1) and their covariances are shown in Table S4.1. Diagonal elements of the covariance matrix represent variances of the parameters.

Table S4.1. Parameters of the EAR model (S4.1) according to Preston et al. (2002)

Parameter	Value	Covariance			
		β	θ	γ_1	γ_2
β	9.74	2.5811			
θ	−0.51	0.078078	0.00991		
γ_1	3.5	0.33353	−0.018353	0.38194	
γ_2	−2.47	−1.0913	−0.011519	−0.46549	1.0273

The model (S4.1) describes the excess absolute risk. Correspondingly, excess relative risk in the target population is estimated by dividing EAR from Eq. (S4.1) by the baseline incidence rate observed in Germany in the year the cancer was diagnosed. The age-dependent baseline rate in the pooled cohort is not available, so the transfer of risk from the pooled cohort to the German population cannot be modelled taking into account the (unknown) ratio of the baseline rates. The LSS is a major (64% of person-years) contributor to the pooled cohort, so baseline rates in the LSS were compared to Germany to estimate the range of baseline ratios. For ProZES, the transfer factor is then modelled stochastically with the assumption that the ratio of baselines is log-uniformly distributed in range from 1/3 to 3, as outlined in the methodology section.

S5. Thyroid cancer, group THYROID (ICD10: C73)

Model description

The thyroid cancer model is based on an analysis of the LSS data with explicit modelling of the screening effect of medical surveillance for members of the so-called Adult Health Study (AHS) (Jacob 2014). The screening effect was found to be statistically significant.

The baseline incidence rate λ_0 (*s*,*a*,*e*,*c*,*AHS*,*NIC*) depends on the explanatory variables of sex s, attained age a, age at exposure e, city (Hiroshima: c=1; Nagasaki c=2), status of participation in the AHS screening program (no: *AHS* = 0; yes: *AHS* = 1) and of having been in the city at the time of bombing (for distance from hypocenter <10 km: *NIC* = 0; otherwise: *NIC* = 1). The baseline incidence rate factorizes

$$\lambda_{0,LSS}(s,a,e,c,AHS,NIC) = \lambda_{0,fit}(s,a,e)F_{scr}(a,e,AHS)g(c,NIC)$$

into a fit function common for all cohort members:

$$\lambda_{0,fit}(s,a,e) = 10^{-4}\exp\left(\beta_{0,s} + \beta_{a_1,s}\ln\frac{a}{60} + \beta_{a_2,s}\ln^2\frac{a}{60} + \beta_{e_1,s}\frac{e-20}{10} + \beta_{e_2,s}\left(\frac{e-20}{60}\right)^2\right)$$

and an adjustment factor accounting for screening effect for the AHS members:

$$F_{scr}(a,e,AHS) = \exp(\beta_{AHS}(a-e)AHS),$$

where for non-zero factor AHS:

$$\beta_{AHS}(a-e) = \begin{cases}\beta_{AHS}, & a-e \geq 25 \text{ (AHS in 1970 and later);}\\ \beta_{AHS,1970}, & a-e < 25 \text{ (AHS before 1970)}\end{cases}$$

and a factor accounting for residential status (city and 'not-in-the-city' factor—NIC):

$$g(c,NIC) = \exp(\beta_c(c-1) + \beta_{NIC}\,NIC).$$

In the further calculations, city- and NIC-status have been averaged out with weights defined from the number of cancer cases observed in each of the sub-groups of the LSS cohort. For the dose response an ERR model was chosen using the form

$$ERR(s,e,a) = \alpha_d\,D\exp\left(\alpha_s\,s + \alpha_a\ln\frac{a}{60} + \alpha_e\frac{e-20}{10}\right)$$

where the α's are the model parameters, D is the thyroid dose and the parameter s equals to +1 for females and to −1 for males.

Table S5.1: MLE estimates and confidence intervals (CIs) for the parameters of the ERR model for risk of thyroid cancer. CIs are calculated from the likelihood profile.

Parameter	Unit	MLE	Confidence interval for probability:	
			P = 0.68	P = 0.95
$\beta_{0,m}$	–	−0.39	(−0.62; −0.17)	–
$\beta_{0,f}$	–	0.53	(0.36; 0.70)	–
β_{city}	–	−0.22	(−0.33; −0.11)	–
β_{AHS}	–	0.21	(0.08; 0.33)	–
$\beta_{AHS,1970}$	–	0.33	(0.22; 0.44)	–
β_{NIC}	–	−0.47	(−0.61; −0.34)	–
$\beta_{a_1,m}$	–	1.9	(1.3; 2.5)	–
$\beta_{a_1,f}$	–	2.0	(1.5; 2.4)	–
$\beta_{a_2,f}$	–	−0.75	(−1.30; −0.24)	–
$\beta_{e_1,m}$	yr^{-1}	0.091	(n.a.; 0.19)	–
$\beta_{e_1,f}$	yr^{-1}	−0.24	(−0.33; −0.16)	–
$\beta_{e_2,f}$	yr^{-1}	0.080	(0.060; 0.098)	–
α_d	Gy^{-1}	1.07	(0.71; 1.51)	(0.44; 2.04)
α_e	–	−0.59	(−0.89; −0.32)	(−1.20;−0.08)
α_a	yr^{-1}	−1.03	(−1.89; −0.16)	(−2.74; 0.70)
α_s	–	0.11	(−0.16; 0.42)	(−0.52; 0.77)

Table S5.2: Covariance matrix for thyroid cancer risk model.

	$\beta_{0,m}$	$\beta_{0,f}$	β_{city}	β_{AHS}	$\beta_{AHS,1970}$	β_{NIC}	$\beta_{a_1,m}$	$\beta_{a_1,f}$	$\beta_{a_2,f}$	$\beta_{e_1,m}$	$\beta_{e_1,f}$	$\beta_{e_2,f}$	α_d	α_e	$\alpha_{\boldsymbol{a}}$	α_s
$\beta_{0,m}$	5,241E-02	2,327E-02	-3,056E-03	-1,892E-02	5,710E-03	-2,224E-03	5,505E-02	1,699E-02	-1,074E-03	-1,357E-02	-2,472E-03	-4,034E-05	-2,749E-03	1,059E-02	-2,086E-02	1,971E-02
$\beta_{0,f}$	2,327E-02	2,884E-02	-3,049E-03	-1,811E-02	5,245E-03	-2,452E-03	1,605E-02	1,796E-02	-6,867E-03	-2,457E-03	-2,898E-03	-4,456E-04	8,365E-03	5,869E-03	-1,805E-02	-2,437E-03
β_{city}	-3,056E-03	-3,049E-03	1,193E-02	-2,150E-04	9,150E-05	9,485E-04	4,324E-04	5,871E-04	3,448E-04	1,813E-04	2,259E-04	-6,845E-06	1,126E-03	-3,210E-05	2,007E-04	1,641E-04
β_{AHS}	-1,892E-02	-1,811E-02	-2,150E-04	1,639E-02	-5,500E-03	-1,483E-03	-1,196E-02	-1,387E-02	-4,604E-03	2,067E-03	1,487E-03	1,828E-04	-1,832E-02	-4,334E-03	-2,793E-03	-1,540E-03
$\beta_{AHS,1970}$	5,710E-03	5,245E-03	9,150E-05	-5,500E-03	1,234E-02	-8,012E-05	2,584E-02	3,053E-02	1,282E-02	-4,817E-03	-4,493E-03	-1,728E-04	2,275E-03	-4,968E-03	1,343E-02	-5,197E-04
β_{NIC}	-2,224E-03	-2,452E-03	9,485E-04	-1,483E-03	-8,012E-05	1,820E-02	-2,594E-04	6,213E-04	-5,353E-05	1,217E-04	5,920E-05	5,509E-05	1,087E-02	3,192E-03	-3,338E-04	9,432E-04
$\beta_{a_1,m}$	5,505E-02	1,605E-02	4,324E-04	-1,196E-02	2,584E-02	-2,594E-04	3,528E-01	7,866E-02	-1,278E-02	-4,479E-02	-1,317E-02	2,426E-04	2,085E-03	9,012E-03	-1,147E-01	-2,482E-02
$\beta_{a_1,f}$	1,699E-02	1,796E-02	5,871E-04	-1,387E-02	3,053E-02	6,213E-04	7,866E-02	1,932E-01	1,081E-01	-1,422E-02	-2,411E-02	-1,325E-05	7,592E-04	9,804E-03	-4,351E-02	6,663E-03
$\beta_{a_2,f}$	-1,074E-03	-6,867E-03	3,448E-04	-4,604E-03	1,282E-02	-5,353E-05	-1,278E-02	1,081E-01	2,795E-01	-8,967E-04	-2,902E-03	-2,816E-03	4,084E-02	-2,315E-02	1,930E-01	-8,443E-03
$\beta_{e_1,m}$	-1,357E-02	-2,457E-03	1,813E-04	2,067E-03	-4,817E-03	1,217E-04	-4,479E-02	-1,422E-02	-8,967E-04	1,048E-02	2,591E-03	-9,358E-05	5,457E-03	-3,190E-03	1,288E-02	-4,067E-03
$\beta_{e_1,f}$	-2,472E-03	-2,898E-03	2,259E-04	1,487E-03	-4,493E-03	5,920E-05	-1,317E-02	-2,411E-02	-2,902E-03	2,591E-03	6,903E-03	-9,600E-04	2,431E-03	-8,304E-03	1,785E-02	5,136E-04
$\beta_{e_2,f}$	-4,034E-05	-4,456E-04	-6,845E-06	1,828E-04	-1,728E-04	5,509E-05	2,426E-04	-1,325E-05	-2,816E-03	-9,358E-05	-9,600E-04	3,761E-04	-2,924E-04	2,230E-03	-4,203E-03	7,320E-05
α_d	-2,749E-03	8,365E-03	1,126E-03	-1,832E-02	2,275E-03	1,087E-02	2,085E-03	7,592E-04	4,084E-02	5,457E-03	2,431E-03	-2,924E-04	1,596E-01	1,162E-02	1,322E-01	-4,987E-02
α_e	1,059E-02	5,869E-03	-3,210E-05	-4,334E-03	-4,968E-03	3,192E-03	9,012E-03	9,804E-03	-2,315E-02	-3,190E-03	-8,304E-03	2,230E-03	1,162E-02	7,686E-02	-1,359E-01	1,104E-02
$\alpha_{\boldsymbol{a}}$	-2,086E-02	-1,805E-02	2,007E-04	-2,793E-03	1,343E-02	-3,338E-04	-1,147E-01	-4,351E-02	1,930E-01	1,288E-02	1,785E-02	-4,203E-03	1,322E-01	-1,359E-01	7,040E-01	-2,217E-02
α_s	1,971E-02	-2,437E-03	1,641E-04	-1,540E-03	-5,197E-04	9,432E-04	-2,482E-02	6,663E-03	-8,443E-03	-4,067E-03	5,136E-04	7,320E-05	-4,987E-02	1,104E-02	-2,217E-02	8,117E-02

S6. Cancer of digestive organs, excluding stomach and colon, group DIG (ICD10: C00–C15, C17, C19–C26, C48)

Table S6.1: Statistical properties of the models fitted to characterize risk of cancers of digestive organs (group DIG) for members of the LSS cohort

Model type (name)	K	Estimated cases		deviance	ΔAIC	AIC-weight (%)
		baseline	excess			
ERR (DIG-ERR14)	14	3970.3	112.7	8030.01	0	71.3
EAR (DIG-EAR14)	14	3983.5	99.5	8031.83	1.82	28.7

The ERR-type model dominates in the generated distribution of assigned share Z and contributes with 71.3% to the total generated sample. The parametric form of the baseline rate appears as follows:

$$\lambda_0(10^{-4}PY^{-1}) = \exp\left(\beta_1 + \beta_2 s + \beta_4 IC + \beta_5 \ln\frac{a}{70} + \beta_8 s \ln^2\frac{a}{70} + \right.$$
$$+ (\beta_9 + \beta_{10}s)\frac{b-1915}{10} + (\beta_{11}+\beta_{12}s)\left(\frac{b-1915}{10}\right)^2 +$$
$$\left. + (\beta_{13}+\beta_{14}s)\max^2\left(0, \ln\frac{a}{\beta_{15}}\right)\right)$$

and the risk function as:

$$ERR = \beta_{19} D \exp\left(\beta_{25}\ln\frac{a}{70}\right)$$

The EAR-type model contributes with 28.7% to the generated sample of Z. For this model, the parametric baseline rate is defined in the following form:

$$\lambda_0(10^{-4}PY^{-1}) = \exp\left(\beta_1 + \beta_2 s + \beta_4 IC + (\beta_5+\beta_6 s)\ln\frac{a}{70} + \beta_8 s \ln^2\frac{a}{70} + \right.$$
$$+ (\beta_9 + \beta_{10}s)\frac{b-1915}{10} + (\beta_{11}+\beta_{12}s)\left(\frac{b-1915}{10}\right)^2 +$$
$$\left. + \beta_{13}\max{}^2\left(0, \ln\frac{a}{\beta_{15}}\right)\right).$$

The EAR is defined with linear dose response, and the only modifier depends on attained age:

$$EAR(10^{-4}PY^{-1}) = \beta_{19} D \exp\left(\beta_{25}\ln\frac{a}{70}\right).$$

Table S6.2: Parameters and parameter statistics for the selected models of the DIG group

Parameter	ERR-type model (ERR14)			EAR-type model (EAR14)		
	Estimate	(p-value)	95%CI	Estimate	(p-value)	95%CI
β_1	3.71	(<0.001)	(3.64; 3.88)	3.71	(<0.001)	(3.63; 3.9)
β_2	−0.51	(<0.001)	(−0.57; −0.46)	−0.45	(<0.001)	(−0.48; −0.41)
β_4	0.078	(0.040)	(0.04; 0.15)	0.083	(0.028)	(0.046; 0.16)
β_5	6.88	(<0.001)	(6.63; 7.43)	6.91	(<0.001)	(6.64; 7.5)
β_6	—	—	—	0.66	(<0.001)	(0.52; 0.96)
β_8	0.95	(<0.001)	(0.41; 1.49)	1.91	(<0.001)	(1.24; 2.6)
β_9	−0.25	(<0.001)	(−0.28; −0.22)	−0.25	(<0.001)	(−0.28;−0.22)
β_{10}	0.045	(0.003)	(0.016; 0.073)	0.041	(0.009)	(0.026; 0.072)
β_{11}	0.020	(0.005)	(0.006; 0.033)	0.019	(0.007)	(0.005; 0.033)
β_{12}	−0.020	(0.003)	(−0.033; −0.007)	−0.019	(0.006)	(−0.033; −0.006)
β_{13}	−6.36	(<0.001)	(−8.24; −4.72)	−6.50	(<0.001)	(−8.6; −4.8)
β_{14}	1.64	(<0.001)	(1.16; 3.0)	—	—	—
β_{15}	58.5	(<0.001)	(54; 63.5)	58.7	(<0.001)	(53.4; 64.4)
β_{19}	0.24	(0.001)	(0.17; 0.40)	6.85	(<0.001)	(4.9; 11)
β_{25}	−3.04	(<0.001)	(−3.9; −1.2)	2.26	(<0.001)	(0.93; 3.7)

Table S6.3: Covariance matrix for the model DIG-ERR14

	β_1	β_2	β_4	β_5	β_8	β_9	β_{10}	β_{11}	β_{12}	β_{13}	β_{14}	β_{15}	β_{19}	β_{25}
β_1	5,271E-03	-4,840E-04	-1,009E-03	1,380E-02	5,838E-04	1,024E-04	-1,104E-04	-4,971E-05	2,299E-05	2,557E-02	-1,647E-02	-1,315E-01	-7,043E-04	-1,514E-02
β_2	-4,840E-04	7,918E-04	-3,100E-05	-1,526E-03	-1,087E-03	5,122E-06	1,336E-04	4,664E-06	-8,241E-05	-6,553E-03	-3,204E-03	2,086E-02	1,029E-04	1,437E-03
β_4	-1,009E-03	-3,100E-05	1,443E-03	1,005E-04	-5,498E-05	-8,872E-06	-8,783E-06	-7,841E-06	5,767E-06	2,968E-05	-7,811E-05	-6,136E-04	-5,652E-04	-2,952E-03
β_5	1,380E-02	-1,526E-03	1,005E-04	6,589E-02	-1,205E-03	-9,456E-04	-4,687E-04	4,039E-04	1,008E-04	2,758E-02	-4,307E-02	-3,702E-01	-2,984E-03	-8,025E-02
β_8	5,838E-04	-1,087E-03	-5,498E-05	-1,205E-03	7,473E-02	2,984E-04	1,359E-03	-9,869E-05	-5,375E-04	1,893E-02	-1,597E-02	-4,057E-02	6,358E-04	1,099E-02
β_9	1,024E-04	5,122E-06	-8,872E-06	-9,456E-04	2,984E-04	2,328E-04	5,244E-05	-3,228E-05	-1,762E-05	1,499E-03	-7,870E-04	-4,655E-03	1,626E-05	4,553E-04
β_{10}	-1,104E-04	1,336E-04	-8,783E-06	-4,687E-04	1,359E-03	5,244E-05	2,168E-04	-1,722E-05	-2,638E-05	-1,419E-03	-2,497E-03	5,046E-03	3,027E-05	4,087E-04
β_{11}	-4,971E-05	4,664E-06	-7,841E-06	4,039E-04	-9,869E-05	-3,228E-05	-1,722E-05	4,741E-05	7,265E-06	-1,463E-03	3,001E-04	1,015E-03	3,295E-06	-1,336E-04
β_{12}	2,299E-05	-8,241E-05	5,767E-06	1,008E-04	-5,375E-04	-1,762E-05	-2,638E-05	7,265E-06	4,529E-05	3,027E-04	-6,813E-05	-8,512E-04	-9,889E-06	-8,867E-05
β_{13}	2,557E-02	-6,553E-03	2,968E-05	2,758E-02	1,893E-02	1,499E-03	-1,419E-03	-1,463E-03	3,027E-04	9,205E-01	-2,615E-01	-1,730E+00	8,810E-04	3,126E-02
β_{14}	-1,647E-02	-3,204E-03	-7,811E-05	-4,307E-02	-1,597E-02	-7,870E-04	-2,497E-03	3,001E-04	-6,813E-05	-2,615E-01	2,399E-01	6,916E-01	1,583E-03	3,393E-02
β_{15}	-1,315E-01	2,086E-02	-6,136E-04	-3,702E-01	-4,057E-02	-4,655E-03	5,046E-03	1,015E-03	-8,512E-04	-1,730E+00	6,916E-01	5,346E+00	1,519E-02	3,073E-01
β_{19}	-7,043E-04	1,029E-04	-5,652E-04	-2,984E-03	6,358E-04	1,626E-05	3,027E-05	3,295E-06	-9,889E-06	8,810E-04	1,583E-03	1,519E-02	5,472E-03	4,590E-02
β_{25}	-1,514E-02	1,437E-03	-2,952E-03	-8,025E-02	1,099E-02	4,553E-04	4,087E-04	-1,336E-04	-8,867E-05	3,126E-02	3,393E-02	3,073E-01	4,590E-02	7,238E-01

Table S6.4: Covariance matrix for the model DIG-EAR14

	β_1	β_2	β_4	β_5	β_6	β_8	β_9	β_{10}	β_{11}	β_{12}	β_{13}	β_{15}	β_{19}	β_{25}
β_1	6,633E-03	1,548E-04	-1,038E-03	1,743E-02	-4,424E-04	-1,575E-03	1,469E-04	-1,992E-05	-5,791E-05	-8,160E-06	4,239E-02	-1,831E-01	-4,064E-03	-4,266E-03
β_2	1,548E-04	5,042E-04	-1,657E-05	3,352E-04	-3,721E-05	-1,606E-03	2,403E-05	-1,068E-05	5,445E-06	-8,029E-05	1,222E-04	-4,185E-03	-2,717E-03	-7,262E-04
β_4	-1,038E-03	-1,657E-05	1,436E-03	-1,988E-05	-2,478E-05	-8,186E-05	-3,156E-06	-7,726E-06	-6,690E-06	6,709E-06	1,052E-04	-4,512E-05	-1,274E-02	-2,291E-03
β_5	1,743E-02	3,352E-04	-1,988E-05	7,603E-02	1,202E-03	-3,112E-03	-9,031E-04	-2,795E-04	4,263E-04	2,406E-05	7,339E-02	-5,125E-01	-1,427E-02	-2,561E-02
β_6	-4,424E-04	-3,721E-05	-2,478E-05	1,202E-03	2,270E-02	2,697E-02	-1,284E-04	-1,406E-03	7,975E-05	9,644E-05	-2,281E-02	2,533E-02	2,733E-03	-9,584E-04
β_8	-1,575E-03	-1,606E-03	-8,186E-05	-3,112E-03	2,697E-02	1,203E-01	5,084E-05	-3,430E-04	2,038E-05	-5,107E-04	-4,151E-02	6,809E-02	1,506E-02	4,687E-03
β_9	1,469E-04	2,403E-05	-3,156E-06	-9,031E-04	-1,284E-04	5,084E-05	2,489E-04	6,411E-05	-3,583E-05	-2,126E-05	2,004E-03	-6,187E-03	-1,339E-03	-1,529E-04
β_{10}	-1,992E-05	-1,068E-05	-7,726E-06	-2,795E-04	-1,406E-03	-3,430E-04	6,411E-05	2,445E-04	-2,090E-05	-3,329E-05	-2,786E-04	1,540E-03	3,781E-05	-8,086E-05
β_{11}	-5,791E-05	5,445E-06	-6,690E-06	4,263E-04	7,975E-05	2,038E-05	-3,583E-05	-2,090E-05	5,052E-05	9,106E-06	-1,633E-03	1,268E-03	-3,223E-04	-2,082E-04
β_{12}	-8,160E-06	-8,029E-05	6,709E-06	2,406E-05	9,644E-05	-5,107E-04	-2,126E-05	-3,329E-05	9,106E-06	4,854E-05	-8,612E-05	3,102E-04	-3,294E-04	6,598E-08
β_{13}	4,239E-02	1,222E-04	1,052E-04	7,339E-02	-2,281E-02	-4,151E-02	2,004E-03	-2,786E-04	-1,633E-03	-8,612E-05	1,134E+00	-2,352E+00	-7,023E-03	3,578E-03
β_{15}	-1,831E-01	-4,185E-03	-4,512E-05	-5,125E-01	2,533E-02	6,809E-02	-6,187E-03	1,540E-03	1,268E-03	3,102E-04	-2,352E+00	7,261E+00	1,137E-01	9,920E-02
β_{19}	-4,064E-03	-2,717E-03	-1,274E-02	-1,427E-02	2,733E-03	1,506E-02	-1,339E-03	3,781E-05	-3,223E-04	-3,294E-04	-7,023E-03	1,137E-01	4,033E+00	9,201E-01
β_{25}	-4,266E-03	-7,262E-04	-2,291E-03	-2,561E-02	-9,584E-04	4,687E-03	-1,529E-04	-8,086E-05	-2,082E-04	6,598E-08	3,578E-03	9,920E-02	9,201E-01	4,628E-01

S7. Cancer of the remaining organs, group REM (ICD10: C30–C32, C37–C41, C45–C47, C49, C62, C74–C76)

Table S7.1: Statistical properties of the models fitted to characterize risk of cancer for remaining organs (group REM) for members of the LSS cohort

Model type (name)	K	Estimated cases		Deviance	ΔAIC	AIC-weight (%)
		baseline	excess			
ERR (REM-ERR11)	11	308.6	15.4	2031.12	—	67.04
EAR (REM-EAR11)	11	309.6	14.4	2032.54	1.42	32.96

The group of "remaining" solid cancers, the REM group, has combined all diagnoses in the LSS cohort, for which the number of the observed cancer cases was not sufficient for statistically significant inference of radiation risk.

Radiation risk modelling was performed using descriptive models of ERR- and EAR-types. Structure of the baseline function was the same for both risk models and appears as follows:

$$\lambda_0 = \exp\left(\beta_1 + \beta_2 s + \beta_4 IC + \beta_5 \ln\frac{a}{70} + \beta_7 \ln^2\frac{a}{70} + \right.$$
$$\left. + \beta_9 \frac{by-1915}{10} + (\beta_{13} + \beta_{14} s)\max^2\left(0, \ln\frac{a}{\beta_{15}}\right)\right)$$

Radiation risk in the ERR-type model is expressed with an age-dependent modifier:

$$ERR = \beta_{19} D \exp\left(\beta_{25} \ln\frac{a}{70}\right),$$

while in the EAR-type model no statistically significant effect modifiers were found:

$$EAR(10^{-4}PY^{-1}) = (\beta_{19} + \beta_{20} s) D.$$

Table S7.2: Parameters and their statistics for the fitted risk models of solid cancers of remaining organs (group REM) among members of the LSS cohort

Parameter	ERR-type model (REM-ERR11)			EAR-type model (REM-EAR11)		
	Estimate	(p-value)	95%CI	Estimate	(p-value)	95%CI
β_1	0.95	(0.007)	(0.27; 2.72)	0.90	(0.037)	(0.48; 4.3)
β_2	−0.57	(<0.001)	(−0.65; −0.41)	−0.56	(<0.001)	(−0.64; −0.37)
β_4	0.41	(0.006)	(0.26; 0.71)	0.41	(0.005)	(0.27; 0.7)
β_5	5.32	(<0.001)	(2.6; 10.3)	5.01	(0.005)	(1.5; 8.6)
β_7	2.03	(0.084)	(−2.5; 4.4)	1.76	(0.25)	(−1.2; 4.8)
β_9	0.12	(0.018)	(0.07; 0.21)	0.12	(0.016)	(0.07; 0.22)
β_{13}	−9.83	(0.001)	(−197; −4.4)	−9.57	(0.005)	(−36; −3.4)
β_{14}	−2.34	(0.12)	(−26; −0.19)	−2.56	(0.18)	(−17.5; 0.23)
β_{15}	55.5	(<0.001)	(46.4; 85.2)	55.9	(<0.001)	(32; 100)
β_{19}	0.25	(0.20)	(0.06; 0.76)	0.60	(0.030)	(0.33; 1.24)
β_{20}	—	—	—	-0.41	(0.136)	(−0.95; 0.048)
β_{25}	−2.77	(0.019)	(−5.1; −0.43)	—	—	—

Table S7.3: Covariance matrix for the model REM-EAR11.

	β_1	β_2	β_4	β_5	β_7	β_9	β_{13}	β_{14}	β_{15}	β_{19}	β_{20}
β_1	1,833E-01	1,063E-02	-1,693E-02	7,018E-01	5,387E-01	-1,893E-03	-2,105E-02	5,152E-01	-2,194E+00	-2,147E-03	-8,946E-05
β_2	1,063E-02	7,204E-03	-3,601E-04	3,391E-02	2,640E-02	8,139E-05	7,605E-03	-2,537E-02	-1,395E-01	2,856E-03	-4,213E-03
β_4	-1,693E-02	-3,601E-04	2,196E-02	2,989E-03	2,257E-03	-2,028E-04	-1,646E-03	2,644E-03	-7,520E-03	-4,754E-03	3,868E-03
β_5	7,018E-01	3,391E-02	2,989E-03	3,250E+00	2,622E+00	-1,701E-02	-1,183E+00	1,970E+00	-8,251E+00	-9,607E-03	-3,437E-04
β_7	5,387E-01	2,640E-02	2,257E-03	2,622E+00	2,338E+00	-8,545E-03	-1,414E+00	1,434E+00	-6,038E+00	-1,482E-02	1,369E-03
β_9	-1,893E-03	8,139E-05	-2,028E-04	-1,701E-02	-8,545E-03	2,614E-03	1,746E-02	7,181E-03	-1,419E-02	6,221E-04	-6,820E-04
β_{13}	-2,105E-02	7,605E-03	-1,646E-03	-1,183E+00	-1,414E+00	1,746E-02	1,150E+01	3,121E+00	-9,608E+00	6,521E-04	9,870E-03
β_{14}	5,152E-01	-2,537E-02	2,644E-03	1,970E+00	1,434E+00	7,181E-03	3,121E+00	3,686E+00	-9,522E+00	-3,033E-02	2,929E-02
β_{15}	-2,194E+00	-1,395E-01	-7,520E-03	-8,251E+00	-6,038E+00	-1,419E-02	-9,608E+00	-9,522E+00	3,802E+01	4,292E-02	-2,277E-02
β_{19}	-2,147E-03	2,856E-03	-4,754E-03	-9,607E-03	-1,482E-02	6,221E-04	6,521E-04	-3,033E-02	4,292E-02	7,726E-02	-6,472E-02
β_{20}	-8,946E-05	-4,213E-03	3,868E-03	-3,437E-04	1,369E-03	-6,820E-04	9,870E-03	2,929E-02	-2,277E-02	-6,472E-02	7,645E-02

Table S7.4: Covariance matrix for the model REM-ERR11.

	β_1	β_2	β_4	β_5	β_7	β_9	β_{13}	β_{14}	β_{15}	β_{19}	β_{25}
β_1	1,241E-01	6,371E-03	-1,796E-02	4,359E-01	3,152E-01	-1,514E-03	-7,692E-02	2,621E-01	-1,257E+00	7,135E-03	5,209E-02
β_2	6,371E-03	6,250E-03	-1,717E-04	1,617E-02	1,203E-02	1,883E-05	-3,590E-04	-3,717E-02	-7,123E-02	4,280E-04	2,483E-03
β_4	-1,796E-02	-1,717E-04	2,224E-02	-3,259E-03	-4,381E-03	-1,219E-04	3,120E-03	-1,496E-03	2,577E-03	-5,506E-03	-1,729E-02
β_5	4,359E-01	1,617E-02	-3,259E-03	2,015E+00	1,554E+00	-1,451E-02	-1,131E+00	9,443E-01	-4,361E+00	4,993E-02	3,784E-01
β_7	3,152E-01	1,203E-02	-4,381E-03	1,554E+00	1,387E+00	-6,643E-03	-1,178E+00	6,386E-01	-2,964E+00	5,948E-02	5,775E-01
β_9	-1,514E-03	1,883E-05	-1,219E-04	-1,451E-02	-6,643E-03	2,429E-03	1,282E-02	6,490E-03	-1,327E-02	-1,160E-04	-3,321E-04
β_{13}	-7,692E-02	-3,590E-04	3,120E-03	-1,131E+00	-1,178E+00	1,282E-02	9,135E+00	2,137E+00	-6,740E+00	-5,625E-02	-5,041E-01
β_{14}	2,621E-01	-3,717E-02	-1,496E-03	9,443E-01	6,386E-01	6,490E-03	2,137E+00	2,303E+00	-5,000E+00	1,002E-02	8,181E-02
β_{15}	-1,257E+00	-7,123E-02	2,577E-03	-4,361E+00	-2,964E+00	-1,327E-02	-6,740E+00	-5,000E+00	2,166E+01	-4,474E-02	-3,633E-01
β_{19}	7,135E-03	4,280E-04	-5,506E-03	4,993E-02	5,948E-02	-1,160E-04	-5,625E-02	1,002E-02	-4,474E-02	3,829E-02	1,783E-01
β_{25}	5,209E-02	2,483E-03	-1,729E-02	3,784E-01	5,775E-01	-3,321E-04	-5,041E-01	8,181E-02	-3,633E-01	1,783E-01	1,402E+00

S8. Cancers of female genital organs, group GNF1 – Uterus/Cervix (ICD10: C53)

Table S8.1: Statistical properties of the models fitted to characterize risk of female cervical cancers (group GNF1) for members of the LSS cohort

Model type (name)	K	Estimated cases: baseline	excess	Deviance	ΔAIC	AIC-weight (%)
EAR (GNF1-EAR8)	8	970.0	8.0	2861.05	–	57.08
ERR (GNF1-ERR8)	8	973.6	4.4	2861.62	0.57	42.92

The fitted models share the similar parametric representation for the baseline:

$$\lambda_0(10^{-4}PY^{-1}) = \exp\left(\beta_1 + \beta_5 \ln\frac{a}{70} + \beta_7 \ln^2\frac{a}{70} + \right.$$
$$\left. + \beta_9 \frac{b-1915}{10} + \beta_{11}\left(\frac{b-1915}{10}\right)^2 + \beta_{13}\max^2\left(0, \ln\frac{a}{\beta_{15}}\right)\right)$$

Both models are simple models with a linear dose-response without effect modifiers. The EAR model is represented by:

$$EAR(10^{-4}PY^{-1}) = \beta_{19} D$$

and the ERR model by:

$$ERR = \beta_{19} D$$

Table S8.2: Parameters and parameter statistics for the selected models of the GNF1 (cervical cancer) model group

Parameter	ERR-type model (GNF1-ERR8)			EAR-type model (GNF1-EAR8)		
	Estimate	(p-value)	95%CI	Estimate	(p-value)	95%CI
β_1	0.91	(0.11)	(−1.3; 1.64)	0.75	(0.23)	(−4.1; 1.6)
β_5	−5.92	(0.006)	(−8.0; −3.0)	−6.62	(0.007)	(−9.0; −3.3)
β_7	−6.69	(<0.001)	(−10.4; −3.9)	−7.40	(0.001)	(−11.9; −4.1)
β_9	0.34	(<0.001)	(0.31; 0.41)	0.35	(<0.001)	(0.32; 0.41)
β_{11}	−0.030	(0.034)	(−0.057; −0.003)	−0.031	(0.031)	(−0.058; −0.003)
β_{13}	6.52	(0.002)	(4.4; 11.6)	7.27	(0.005)	(4.7; 14.6)
β_{15}	48.5	(<0.001)	(40.5; 60)	48.0	(<0.001)	(35.5; 59)
β_{19}	0.06	(0.68)	(−0.08; 0.35)	0.57	(0.41)	(−0.11; 2.0)

Table S8.3: Covariance matrix for the model GNF1-EAR8

	β_1	β_5	β_7	β_9	β_{11}	β_{13}	β_{15}	β_{19}
β_1	3,874E-01	1,511E+00	1,361E+00	-1,707E-03	1,784E-04	-1,387E+00	1,628E+00	-1,344E-01
β_5	1,511E+00	6,066E+00	5,579E+00	-8,892E-03	1,474E-03	-5,842E+00	5,786E+00	-5,622E-01
β_7	1,361E+00	5,579E+00	5,278E+00	-5,433E-03	3,053E-04	-5,567E+00	4,816E+00	-5,632E-01
β_9	-1,707E-03	-8,892E-03	-5,433E-03	1,059E-03	-7,939E-05	6,037E-03	-4,084E-04	2,686E-03
β_{11}	1,784E-04	1,474E-03	3,053E-04	-7,939E-05	1,999E-04	-3,555E-03	7,000E-05	-6,102E-04
β_{13}	-1,387E+00	-5,842E+00	-5,567E+00	6,037E-03	-3,555E-03	6,775E+00	-3,442E+00	5,861E-01
β_{15}	1,628E+00	5,786E+00	4,816E+00	-4,084E-04	7,000E-05	-3,442E+00	1,024E+01	-3,899E-01
β_{19}	-1,344E-01	-5,622E-01	-5,632E-01	2,686E-03	-6,102E-04	5,861E-01	-3,899E-01	4,696E-01

Table S8.4: Covariance matrix for the model GNF1-ERR8

	β_1	β_5	β_7	β_9	β_{11}	β_{13}	β_{15}	β_{19}
β_1	3,172E-01	1,183E+00	1,006E+00	-6,870E-04	-7,482E-05	-9,164E-01	1,964E+00	-1,219E-03
β_5	1,183E+00	4,566E+00	3,984E+00	-4,718E-03	4,550E-04	-3,824E+00	6,746E+00	1,047E-03
β_7	1,006E+00	3,984E+00	3,603E+00	-1,448E-03	-6,422E-04	-3,534E+00	5,342E+00	6,149E-04
β_9	-6,870E-04	-4,718E-03	-1,448E-03	1,023E-03	-7,113E-05	2,040E-03	3,493E-03	1,432E-05
β_{11}	-7,482E-05	4,550E-04	-6,422E-04	-7,113E-05	1,947E-04	-2,580E-03	-8,506E-04	1,861E-05
β_{13}	-9,164E-01	-3,824E+00	-3,534E+00	2,040E-03	-2,580E-03	4,523E+00	-2,933E+00	2,121E-04
β_{15}	1,964E+00	6,746E+00	5,342E+00	3,493E-03	-8,506E-04	-2,933E+00	1,652E+01	2,403E-03
β_{19}	-1,219E-03	1,047E-03	6,149E-04	1,432E-05	1,861E-05	2,121E-04	2,403E-03	1,783E-02

S9. Cancer of female genital organs, group GNF2 – Uterus/Corpus and other (ICD10: C51–C52, C54–C58)

Table S9.1: Statistical properties of the models fitted to characterize risk of cancers of female genital organs other than cervix uteri (group GNF2) for members of the LSS cohort

Model type (name)	K	Estimated cases: baseline	excess	Deviance	ΔAIC	AIC-weight (%)
EAR (GNF2-EAR5)	5	472.0	7.0	1970.87	1.93	27.59
ERR (GNF2-ERR5)	5	465.9	13.1	1969.11	—	72.41

The fitted models have the same structure of the parametric baseline, which appear as follows:

$$\lambda_0(10^{-4}PY^{-1}) = \exp\left(\beta_1 + \beta_5 \ln\frac{a}{70} + \beta_7 \ln^2\frac{a}{70} + \beta_9 \frac{b-1915}{10}\right).$$

The statistical significance was not sufficient to identify effect modifiers. Therefore, both for the EAR- and ERR-type model, risk functions depend linearly on dose without additional age dependencies. The EAR model is represented by:

$$EAR(10^{-4}PY^{-1}) = \beta_{19} D$$

and the ERR model by:

$$ERR = \beta_{19} D.$$

Table S9.2: Parameters and parameter statistics for the selected models of the GNF2 group (female genital organs, excluding cervix uteri)

Parameter	ERR-type model (GNF2-ERR5)			EAR-type model (GNF2-EAR5)		
	Estimate	(p-value)	95%CI	Estimate	(p-value)	95%CI
β_1	1.49	(<0.001)	(1.4; 1.6)	1.51	(<0.001)	(1.4; 1.6)
β_5	1.1	(0.009)	(0.28; 1.9)	1.07	(0.012)	(0.24; 1.9)
β_7	−2.98	(<0.001)	(−4.4; −1.6)	−3.13	(<0.001)	(−4.6; −1.7)
β_9	−0.12	(0.004)	(−0.21; −0.04)	−0.12	(0.005)	(−0.21; −0.04)
β_{19}	0.35	(0.12)	(0.13; 0.86)	0.49	(0.30)	(0.03; 1.6)

Table S9.3: Covariance matrix for the model GNF2-EAR5

	β_1	β_5	β_7	β_9	β_{19}
β_1	3,230E-03	6,747E-03	-4,422E-03	-6,243E-04	-3,824E-03
β_5	6,747E-03	1,807E-01	2,388E-01	-9,865E-03	-4,304E-03
β_7	-4,422E-03	2,388E-01	5,774E-01	-5,541E-03	-3,777E-02
β_9	-6,243E-04	-9,865E-03	-5,541E-03	1,856E-03	6,006E-04
β_{19}	-3,824E-03	-4,304E-03	-3,777E-02	6,006E-04	2,195E-01

Table S9.4: Covariance matrix for the model GNF2-ERR5

	β_1	β_5	β_7	β_9	β_{19}
β_1	3,372E-03	6,579E-03	-4,235E-03	-5,963E-04	-3,845E-03
β_5	6,579E-03	1,745E-01	2,244E-01	-9,658E-03	1,707E-03
β_7	-4,235E-03	2,244E-01	5,184E-01	-5,345E-03	1,789E-03
β_9	-5,963E-04	-9,658E-03	-5,345E-03	1,814E-03	2,126E-05
β_{19}	-3,845E-03	1,707E-03	1,789E-03	2,126E-05	5,009E-02

S10. Cancer of male genital organs, group GNM (ICD10: C60–C61, C63)

Table S10.1: Statistical properties of the models fitted to characterize risk of cancers of male genital organs (group GNM) for members of the LSS cohort

Model type (name)	K	Estimated cases: baseline	excess	Deviance	ΔAIC	AIC-weight (%)
EAR (GNM-EAR7)	7	396.2	6.8	1518.05	5.565	4.72
ERR (GNM-ERR8a)	8	398.6	4.4	1510.48	—	76.35
ERR (GNM-ERR8b)	8	396.7	6.3	1513.27	2.789	18.93

Modelling of radiation risk of cancers of male genital organs (dominated by cancers of prostate: 387 of 403 considered) resulted in three models (see Table S10.1).

In the selected models, the parametric baseline is described using a simple representation which accounts only for effects of age and birth year. However, despite of general similarity, the baseline rate representations for different models vary. For example, the baseline equation for the 'GNM EAR7' model includes no quadratic spline term and appears as follows:

$$\lambda_0(10^{-4}PY^{-1}) = \exp\left(\beta_1 + \beta_5 \ln\frac{a}{70} + \beta_7 \ln^2\frac{a}{70} + \beta_9 \frac{b-1915}{10} + \beta_{11}\left(\frac{b-1915}{10}\right)^2\right)$$

with the risk function of the following form:

$$EAR(10^{-4}PY^{-1}) = \beta_{19} D \exp\left(\beta_{25} \ln\frac{a}{70}\right).$$

The model 'GNM-ERR8a' is formulated with the following baseline function:

$$\lambda_0(10^{-4}PY^{-1}) = \exp\left(\beta_1 + \beta_5 \ln\frac{a}{70} + \beta_7 \ln^2\frac{a}{70} + \right.$$
$$\left. + \beta_9 \frac{b-1915}{10} + \beta_{11}\left(\frac{b-1915}{10}\right)^2 + \beta_{13}\max^2\left(0, \ln\frac{a}{\beta_{15}}\right)\right)$$

and with a simple, linear dose-response risk model:

$$ERR = \beta_{19} D.$$

The model 'GNM-ERR8b' has a simpler baseline function:

$$\lambda_0(10^{-4}PY^{-1}) = \exp\left(\beta_1 + \beta_5 \ln\frac{a}{70} + \beta_7 \ln^2\frac{a}{70} + \beta_9 \frac{b-1915}{10} + \beta_{13}\max^2\left(0, \ln\frac{a}{\beta_{15}}\right)\right)$$

with radiation risk including also an attained-age effect modifier:

$$ERR = \beta_{19} D \exp\left(\beta_{25} \ln\frac{a}{70}\right)$$

Parameter values and statistics are shown in Table S10.2. As seen from the table, parameters of the radiation risk functions show low statistical significance.

Table S10.2: Parameters and parameter statistics for the selected models of the group GNM for cancers of male genital organs

Parameter	EAR-type model (GNM-EAR7)		ERR-type model (GNM-ERR8a)		ERR-type model (GNM-ERR8b)	
	Estimate (p-value)	95%CI	Estimate (p-value)	95%CI	Estimate (p-value)	95%CI
β_1	2.36 (<0.001)	(2.28; 2.51)	2.78 (<0.001)	(2.39; 4.7)	2.75 (<0.001)	(2.38; 4.8)
β_5	10.8 (<0.001)	(9.3; 12.3)	17.5 (<0.001)	(11.7; 25.4)	16.1 (<0.001)	(10.6; 27.6)
β_7	−13.8 (<0.001)	(−17.2; −6.9)	8.2 (0.010)	(−4.2; 9.8)	6.82 (0.087)	(−8.6; 8.8)
β_9	−0.32 (<0.001)	(−0.45; −0.19)	−0.32 (<0.001)	(−0.39; −0.2)	−0.25 (<0.001)	(−0.34; −0.15)
β_{11}	0.043 (0.078)	(−0.005; 0.09)	0.044 (0.061)	(−0.002; 0.089)	—	—
β_{13}	—	—	−24.5 (<0.001)	(−99.7; −9.98)	−21.3 (<0.001)	(−341; −6.6)
β_{15}	—	—	61.5 (<0.001)	(54.2; 110)	61.7 (<0.001)	(54; 114)
β_{19}	1.39 (0.38)	(−0.14; 5.2)	0.12 (0.56)	(−0.083; 0.62)	0.20 (0.37)	(−0.016; 0.74)
β_{25}	2.7 (0.27)	(−7.0; 42.6)	—	—	−3.7 (0.33)	(−7.4; 26.7)

Table S10.3: Covariance matrix for the model GNM-EAR7

	β_1	β_5	β_7	β_9	β_{11}	β_{19}	β_{25}
β_1	6,230E-03	-3,331E-03	-8,874E-02	-2,726E-05	-5,558E-04	-2,386E-02	-3,509E-02
β_5	-3,331E-03	5,675E-01	-1,733E+00	-3,415E-02	8,526E-03	2,681E-01	-7,107E-02
β_7	-8,874E-02	-1,733E+00	1,217E+01	9,246E-02	-3,428E-02	-8,625E-01	1,001E+00
β_9	-2,726E-05	-3,415E-02	9,246E-02	4,435E-03	-1,165E-03	-1,244E-02	9,736E-05
β_{11}	-5,558E-04	8,526E-03	-3,428E-02	-1,165E-03	5,970E-04	4,193E-03	2,270E-04
β_{19}	-2,386E-02	2,681E-01	-8,625E-01	-1,244E-02	4,193E-03	2,467E+00	2,725E+00
β_{25}	-3,509E-02	-7,107E-02	1,001E+00	9,736E-05	2,270E-04	2,725E+00	6,138E+00

Table S10.4: Covariance matrix for the model GNM-ERR8a

	β_1	β_5	β_7	β_9	β_{11}	β_{13}	β_{15}	β_{19}
β_1	1,616E-01	1,075E+00	9,293E-01	-1,094E-03	2,202E-04	-3,885E-01	-1,419E+00	-4,497E-03
β_5	1,075E+00	8,772E+00	8,092E+00	-4,933E-02	1,676E-02	-7,264E+00	-8,964E+00	-1,514E-03
β_7	9,293E-01	8,092E+00	1,020E+01	-2,773E-02	9,173E-03	-9,684E+00	-7,234E+00	-8,757E-04
β_9	-1,094E-03	-4,933E-02	-2,773E-02	3,991E-03	-1,025E-03	9,625E-02	-5,351E-03	-1,649E-05
β_{11}	2,202E-04	1,676E-02	9,173E-03	-1,025E-03	5,476E-04	-3,408E-02	-1,859E-03	3,026E-05
β_{13}	-3,885E-01	-7,264E+00	-9,684E+00	9,625E-02	-3,408E-02	2,338E+01	-9,923E-01	5,371E-03
β_{15}	-1,419E+00	-8,964E+00	-7,234E+00	-5,351E-03	-1,859E-03	-9,923E-01	1,383E+01	2,067E-03
β_{19}	-4,497E-03	-1,514E-03	-8,757E-04	-1,649E-05	3,026E-05	5,371E-03	2,067E-03	4,536E-02

Table S10.5: Covariance matrix for the model GNM-ERR8b

	β_1	β_5	β_7	β_9	β_{13}	β_{15}	β_{19}	β_{25}
β_1	1,423E-01	9,835E-01	1,025E+00	-1,093E-03	-5,812E-01	-1,409E+00	-6,429E-03	3,080E-01
β_5	9,835E-01	8,062E+00	9,262E+00	-1,863E-02	-8,231E+00	-9,166E+00	-3,581E-03	3,162E+00
β_7	1,025E+00	9,262E+00	1,585E+01	-1,089E-02	-1,499E+01	-8,457E+00	-8,795E-02	9,181E+00
β_9	-1,093E-03	-1,863E-02	-1,089E-02	2,180E-03	3,065E-02	-6,687E-03	-1,673E-06	-1,294E-04
β_{13}	-5,812E-01	-8,231E+00	-1,499E+01	3,065E-02	2,664E+01	1,362E-01	6,377E-02	-8,464E+00
β_{15}	-1,409E+00	-9,166E+00	-8,457E+00	-6,687E-03	1,362E-01	1,586E+01	8,564E-03	-1,896E+00
β_{19}	-6,429E-03	-3,581E-03	-8,795E-02	-1,673E-06	6,377E-02	8,564E-03	5,073E-02	3,908E-03
β_{25}	3,080E-01	3,162E+00	9,181E+00	-1,294E-04	-8,464E+00	-1,896E+00	3,908E-03	1,428E+01

S11. Cancer of urinary organs, group URI (ICD10: C64–C68)

Table S11.1: Statistical properties of the models fitted to characterize risk of cancers of urinary tract organs (group URI) for members of the LSS cohort

Model type (name)	K	Estimated cases		Deviance	ΔAIC	AIC weight (%)
		baseline	excess			
ERR (URI-ERR9)	9	682.3	58.7	3368.67	0.24	47.0
EAR (URI-EAR10)	10	687.0	54.0	3366.43	–	53.0

The group of urinary cancers (URI) combines cases diagnosed with cancer of kidney (178 cases), renal pelvis and ureter (92 cases), urinary bladder (511), and other parts of urinary system (26 cases). The ERR-type model has a baseline of the form:

$$\lambda_0(10^{-4}PY^{-1}) = \exp\left(\beta_1 + \beta_2 s + \beta_5 \ln\frac{a}{70} + \right.$$
$$\left. + \beta_9 \frac{b-1915}{10} + \beta_{11}\left(\frac{b-1915}{10}\right)^2 + \beta_{13}\max^2\left(0, \ln\frac{a}{\beta_{15}}\right)\right)$$

and the risk is represented as:

$$ERR = (\beta_{19} + \beta_{20} s)\, D.$$

The baseline of the EAR-type model has the following form:

$$\lambda_0(10^{-4}PY^{-1}) = \exp\left(\beta_1 + \beta_2 s + \beta_5 \ln\frac{a}{70} + \beta_7 \ln^2\frac{a}{70} + \right.$$
$$\left. + \beta_9 \frac{b-1915}{10} + \beta_{11}\left(\frac{b-1915}{10}\right)^2 + \beta_{13}\max^2\left(0, \ln\frac{a}{\beta_{15}}\right)\right)$$

and the risk is represented as:

$$EAR(10^{-4}PY^{-1}) = \beta_{19} D \exp\left(\beta_{25}\ln\frac{a}{70}\right).$$

Table S11.2: Parameters and parameter statistics for the selected models of the URI (cancers of urinary tract organs) model group

Parameter	ERR-type model (URI-ERR9)			EAR-type model (URI-EAR10)		
	Estimate	(p-value)	95%CI	Estimate	(p-value)	95%CI
β_1	1.80	(<0.001)	(1.72; 1.98)	2.25	(<0.001)	(1.9; 3.7)
β_2	−0.66	(<0.001)	(−0.75; −0.58)	−0.66	(<0.001)	(−0.74; −0.58)
β_5	7.07	(<0.001)	(6.65; 8.0)	10.6	(<0.001)	(8.8; 14)
β_7	—	—	—	4.1	(0.018)	(0.72; 4.4)
β_9	−0.26	(<0.001)	(−0.33; −0.19)	−0.29	(<0.001)	(−0.36; −0.21)
β_{11}	0.033	(0.035)	(0.002; 0.063)	0.040	(0.017)	(0.007; 0.072)
β_{13}	−14.6	(0.015)	(−26.3; −6.6)	−13.4	(<0.001)	(−19; −7.7)
β_{15}	69.6	(<0.001)	(62.3; 76.8)	58.6	(<0.001)	(49.3; 72)
β_{19}	1.21	(<0.001)	(0.94; 1.85)	4.2	(<0.001)	(3.2; 6.4)
β_{20}	0.73	(0.011)	(0.45; 1.36)	—	—	—
β_{25}	—	—	—	3.6	(<0.001)	(2.7; 5.7)

Table S11.3: Covariance matrix for the model URI-EAR10.

	β_1	β_2	β_5	β_7	β_9	β_{11}	β_{13}	β_{15}	β_{19}	β_{25}
β_1	1,271E-01	5,962E-04	6,191E-01	5,195E-01	7,290E-04	-1,603E-04	2,746E-02	-1,593E+00	-8,526E-03	-2,023E-02
β_2	5,962E-04	1,747E-03	-2,548E-04	-2,028E-04	5,195E-05	7,027E-05	-1,792E-03	-1,384E-03	-6,699E-03	-2,691E-03
β_5	6,191E-01	-2,548E-04	3,443E+00	3,012E+00	-9,588E-03	4,693E-03	-1,126E+00	-7,324E+00	1,615E-02	-1,325E-01
β_7	5,195E-01	-2,028E-04	3,012E+00	3,008E+00	-4,828E-03	2,428E-03	-1,575E+00	-5,883E+00	2,464E-02	-5,339E-02
β_9	7,290E-04	5,195E-05	-9,588E-03	-4,828E-03	1,457E-03	-2,557E-04	2,439E-02	-2,055E-02	-8,952E-04	6,488E-04
β_{11}	-1,603E-04	7,027E-05	4,693E-03	2,428E-03	-2,557E-04	2,783E-04	-1,321E-02	2,862E-03	7,338E-05	-6,007E-04
β_{13}	2,746E-02	-1,792E-03	-1,126E+00	-1,575E+00	2,439E-02	-1,321E-02	7,897E+00	-4,426E+00	5,996E-02	9,252E-02
β_{15}	-1,593E+00	-1,384E-03	-7,324E+00	-5,883E+00	-2,055E-02	2,862E-03	-4,426E+00	2,257E+01	-1,090E-01	1,449E-01
β_{19}	-8,526E-03	-6,699E-03	1,615E-02	2,464E-02	-8,952E-04	7,338E-05	5,996E-02	-1,090E-01	1,096E+00	4,173E-01
β_{25}	-2,023E-02	-2,691E-03	-1,325E-01	-5,339E-02	6,488E-04	-6,007E-04	9,252E-02	1,449E-01	4,173E-01	8,479E-01

Table S11.4: Covariance matrix for the model URI-ERR 9.

	β_1	β_2	β_5	β_9	β_{11}	β_{13}	β_{15}	β_{19}	β_{20}
β_1	5,552E-03	4,581E-04	1,508E-02	-1,608E-05	-2,801E-04	1,626E-01	-1,698E-01	-6,374E-03	-3,842E-03
β_2	4,581E-04	1,899E-03	-5,879E-04	2,849E-05	6,459E-05	-2,069E-03	-1,933E-03	-3,692E-03	-6,007E-03
β_5	1,508E-02	-5,879E-04	1,846E-01	-7,895E-03	2,485E-03	3,689E-01	-7,594E-01	5,456E-04	1,887E-04
β_9	-1,608E-05	2,849E-05	-7,895E-03	1,241E-03	-2,068E-04	2,050E-02	-6,450E-03	-4,898E-05	4,011E-05
β_{11}	-2,801E-04	6,459E-05	2,485E-03	-2,068E-04	2,414E-04	-7,231E-03	-4,503E-03	7,095E-05	1,079E-05
β_{13}	1,626E-01	-2,069E-03	3,689E-01	2,050E-02	-7,231E-03	3,585E+01	-1,926E+01	-1,593E-03	2,250E-03
β_{15}	-1,698E-01	-1,933E-03	-7,594E-01	-6,450E-03	-4,503E-03	-1,926E+01	1,389E+01	9,377E-03	3,798E-03
β_{19}	-6,374E-03	-3,692E-03	5,456E-04	-4,898E-05	7,095E-05	-1,593E-03	9,377E-03	8,214E-02	5,567E-02
β_{20}	-3,842E-03	-6,007E-03	1,887E-04	4,011E-05	1,079E-05	2,250E-03	3,798E-03	5,567E-02	8,204E-02

S12. Cancer of brain and central nervous system, group BCNS (ICD10: C69–C72)

Table S12.1: Statistical properties of the models fitted to characterize risk of cancers of brain and central nervous system (group BCNS) for members of the LSS cohort

Model type (name)	K	Estimated cases [a]		Deviance	ΔAIC	AIC-weight (%)
		baseline	excess			
EAR (BCNS-EAR6)	6	236.9	10.2	1870.31	0.65	41.91
ERR (BCNS-ERR7)	7	233.9	12.1	1867.66	—	58.09

[a] Screening effect of the autopsy program (mostly, before 1970) is excluded

The fitted models have the following common parametric form for the function describing the baseline rate:

$$\lambda_0(10^{-4}\,PY^{-1}) = \exp\left(\beta_1 + \beta_4 IC + \beta_5 \ln\frac{a}{70} + \beta_9 \frac{b-1915}{10}\right),$$

and the radiation risk is specified using a constant linear dose-response EAR-type model:

$$EAR(10^{-4}\,PY^{-1}) = \beta_{19} D,$$

and a linear dose-response ERR-type model with an attained age effect modifier:

$$ERR = \beta_{19} D \exp\left(\beta_{25} \ln\frac{a}{70}\right).$$

For cancers of the BCNS group, the screening factor for the LSS cohort is

$$F_{scr} = \exp\left(\beta_{33} \mathrm{sign}\left(cy - 1970\right)\right)$$

where sgn is +1 for positive value of the argument, and -1 for negative values.

Table S12.2: Parameters and their statistics for the BCNS cancer group risk models

Parameter	ERR-type model (BCNS-ERR7)			EAR-type model (BCNS-EAR6)		
	Estimate	(p-value)	95%CI	Estimate	(p-value)	95%CI
β_1	0.26	(0.16)	(−0.10; 0.61)	0.23	(0.21)	(−0.13; 0.57)
β_4	0.63	(<0.001)	(0.46; 0.99)	0.64	(<0.001)	(0.47; 0.99)
β_5	3.91	(<0.001)	(3.42; 4.92)	3.77	(<0.001)	(3.28; 4.76)
β_9	−0.36	(<0.001)	(−0.52; −0.19)	−0.34	(<0.001)	(−0.51; −0.17)
β_{19}	0.24	(0.23)	(0.045; 0.79)	0.46	(0.046)	(0.24; 0.99)
β_{25}	−2.97	(0.009)	(−5.2; −0.66)	—	—	—
β_{33}	−0.31	(0.007)	(−0.53; −0.086)	−0.27	(0.015)	(−0.49; −0.06)

Table S12.3: Covariance matrix for the model BCNS-EAR6

	β_1	β_4	β_5	β_9	β_{19}	β_{33}
β_1	3,346E-02	-2,465E-02	4,294E-02	-6,459E-03	-2,826E-03	-9,378E-03
β_4	-2,465E-02	2,998E-02	1,329E-03	-3,866E-04	-4,701E-03	-5,709E-04
β_5	4,294E-02	1,329E-03	2,485E-01	-3,614E-02	-7,879E-03	-4,088E-02
β_9	-6,459E-03	-3,866E-04	-3,614E-02	7,313E-03	2,543E-03	7,404E-03
β_{19}	-2,826E-03	-4,701E-03	-7,879E-03	2,543E-03	5,358E-02	5,091E-03
β_{33}	-9,378E-03	-5,709E-04	-4,088E-02	7,404E-03	5,091E-03	1,236E-02

Table S12.4: Covariance matrix for the model BCNS-ERR7

	β_1	β_4	β_5	β_9	β_{19}	β_{25}	β_{33}
β_1	3,360E-02	-2,501E-02	4,423E-02	-6,476E-03	-7,774E-04	-1,910E-02	-9,576E-03
β_4	-2,501E-02	3,025E-02	-6,719E-05	-1,508E-04	-5,820E-03	-1,824E-02	-6,060E-05
β_5	4,423E-02	-6,719E-05	2,567E-01	-3,613E-02	-4,392E-03	-1,346E-01	-4,251E-02
β_9	-6,476E-03	-1,508E-04	-3,613E-02	7,065E-03	9,906E-04	1,215E-02	7,459E-03
β_{19}	-7,774E-04	-5,820E-03	-4,392E-03	9,906E-04	3,791E-02	1,785E-01	9,478E-04
β_{25}	-1,910E-02	-1,824E-02	-1,346E-01	1,215E-02	1,785E-01	1,281E+00	1,027E-02
β_{33}	-9,576E-03	-6,060E-05	-4,251E-02	7,459E-03	9,478E-04	1,027E-02	1,260E-02

S13. Non-melanoma skin cancer, group SKIN (ICD10: C44)

Table S13.1: Statistical properties of the models fitted to characterize risk of non-melanoma skin cancers (group SKIN) for members of the LSS cohort

Model type (name)	K	Estimated cases		Deviance	ΔAIC	AIC-weight (%)
		baseline	excess			
ERR (SKIN-ERR8)	8	291.4	38.6	2061.85	—	71.71
EAR (SKIN-EAR10)	10	293.1	36.9	2059.71	1.86	28.29

The fitted risk models, one of ERR-type and one of EAR-type, share the same form of parametric baseline:

$$\lambda_0(10^{-4}\,PY^{-1}) = \exp\left(\beta_1 + \beta_2 s + \beta_4 IC + (\beta_5 + \beta_6 s)\ln\frac{a}{70} + \beta_7 \ln^2\frac{a}{70}\right)$$

Radiation risk for skin cancer demonstrates essentially a non-linear dose response, and the model fitting resulted in a risk function with a dose response to the power of 1.55 for the ERR-type model and to the power of 1.60 for the EAR-type model (see equation below and Table S13.2). Besides the dose response, the risk function for the ERR-type model only has an age-at-exposure modifier:

$$ERR = \beta_{19} D^{\beta_{21}} \exp\left(\beta_{29}\frac{e-30}{10}\right),$$

while the risk function for the EAR-type model also depends on attained age:

$$EAR\left(10^{-4}\,PY^{-1}\right) = \beta_{19} D^{\beta_{21}} \exp\left(\beta_{25}\ln\frac{a}{70} + \beta_{29}\frac{e-30}{10}\right).$$

Table S13.2: Maximum likelihood estimates (MLE) and statistics of parameters for the selected models of radiation risk for non-melanoma skin cancers (group SKIN)

Parameter	ERR-type model (SKIN-ERR8)			EAR-type model (SKIN-EAR10)		
	Estimate	(p-value)	95%CI	Estimate	(p-value)	95%CI
β_1	0.30	(0.028)	(0.03; 0.56)	0.31	(0.027)	(0.04; 0.57)
β_2	−0.12	(0.029)	(−0.18; −0.01)	−0.16	(0.015)	(−0.22; −0.03)
β_4	0.35	(0.022)	(0.20; 0.65)	0.35	(0.020)	(0.20; 0.66)
β_5	6.53	(<0.001)	(6.2; 7.2)	6.33	(<0.001)	(5.6; 7.1)
β_6	—	—	—	0.91	(0.010)	(0.57; 1.6)
β_7	3.05	(<0.001)	(2.1; 3.9)	2.36	(0.001)	(0.9; 3.7)
β_{19}	0.71	(0.018)	(0.42; 1.46)	1.12	(0.021)	(0.65; 2.3)
β_{21}	1.55	(<0.001)	(1.3; 2.1)	1.60	(<0.001)	(1.3; 2.2)
β_{25}	—	—	—	3.65	(<0.001)	(2.7; 5.9)
β_{29}	−0.89	(<0.001)	(−1.26; −0.56)	−0.75	(<0.001)	(−1.2; −0.35)

Table S13.3: Covariance matrix for the model SKIN-EAR10.

	β_1	β_2	β_4	β_5	β_6	β_7	β_{19}	β_{21}	β_{25}	β_{29}
β_1	1,953E-02	-6,551E-04	-1,804E-02	-8,772E-03	-1,256E-04	-2,151E-02	-1,132E-03	2,746E-04	-1,944E-03	-2,820E-05
β_2	-6,551E-04	4,181E-03	-1,720E-04	-4,834E-03	-3,660E-03	2,555E-03	-7,158E-04	3,006E-04	1,415E-03	-4,623E-04
β_4	-1,804E-02	-1,720E-04	2,280E-02	-1,038E-03	-3,159E-04	-1,096E-03	-7,426E-03	2,583E-03	-1,154E-03	-1,969E-03
β_5	-8,772E-03	-4,834E-03	-1,038E-03	1,388E-01	-2,146E-02	1,518E-01	1,502E-02	-9,066E-03	-2,347E-03	1,745E-03
β_6	-1,256E-04	-3,660E-03	-3,159E-04	-2,146E-02	1,246E-01	-1,276E-01	-1,737E-03	-3,625E-04	-2,042E-02	1,886E-03
β_7	-2,151E-02	2,555E-03	-1,096E-03	1,518E-01	-1,276E-01	5,493E-01	2,101E-02	-3,444E-04	6,831E-02	-8,403E-04
β_{19}	-1,132E-03	-7,158E-04	-7,426E-03	1,502E-02	-1,737E-03	2,101E-02	2,346E-01	-7,571E-02	9,415E-02	5,822E-02
β_{21}	2,746E-04	3,006E-04	2,583E-03	-9,066E-03	-3,625E-04	-3,444E-04	-7,571E-02	6,808E-02	1,965E-02	-1,169E-02
β_{25}	-1,944E-03	1,415E-03	-1,154E-03	-2,347E-03	-2,042E-02	6,831E-02	9,415E-02	1,965E-02	9,437E-01	-1,054E-01
β_{29}	-2,820E-05	-4,623E-04	-1,969E-03	1,745E-03	1,886E-03	-8,403E-04	5,822E-02	-1,169E-02	-1,054E-01	5,011E-02

Table S13.4: Covariance matrix for the model SKIN-ERR8.

	β_1	β_2	β_4	β_5	β_7	β_{19}	β_{21}	β_{29}
β_1	1,914E-02	-6,570E-04	-1,805E-02	-7,860E-03	-1,267E-02	-1,135E-03	3,004E-04	-8,182E-05
β_2	-6,570E-04	3,287E-03	-1,792E-04	-1,703E-03	-8,357E-04	-4,791E-05	3,173E-04	-1,142E-04
β_4	-1,805E-02	-1,792E-04	2,291E-02	-1,071E-03	-1,166E-03	-8,332E-03	3,298E-03	-1,866E-03
β_5	-7,860E-03	-1,703E-03	-1,071E-03	1,188E-01	9,922E-02	1,374E-02	-7,749E-03	-1,032E-02
β_7	-1,267E-02	-8,357E-04	-1,166E-03	9,922E-02	2,226E-01	1,597E-02	-2,605E-03	1,068E-02
β_{19}	-1,135E-03	-4,791E-05	-8,332E-03	1,374E-02	1,597E-02	9,095E-02	-5,182E-02	3,612E-02
β_{21}	3,004E-04	3,173E-04	3,298E-03	-7,749E-03	-2,605E-03	-5,182E-02	7,176E-02	-6,567E-03
β_{29}	-8,182E-05	-1,142E-04	-1,866E-03	-1,032E-02	1,068E-02	3,612E-02	-6,567E-03	3,589E-02

S14. Leukaemia group HEM1 (ICD10: C91.0, C91.3, C91.9)

The leukaemia group HEM1 includes the following diagnoses: acute lymphoblastic leukaemia (ALL), prolymphocytic leukaemia of B-cell type, lymphoid leukaemia/unspecified

The risk models of the group HEM1 are represented by the two EAR-type models:

- EAR-LNT model of EAR-type with linear non-threshold (LNT) dose response (w_{AIC} =90.18%);
- EAR-QDR model of EAR-type with pure quadratic (QDR) dose response (w_{AIC} = 9.82%).

The models share the same form of the parametric baseline:

$$\lambda_0(10^{-4}PY^{-1}) = \exp\left(\beta_1 + \beta_2 \ln\frac{a}{70} + \beta_3 NIC_{Hi} + \beta_4 NIC_{Na}\right).$$

NIC is an indicator variable for not-in-city at the time of bombing in Hiroshima (Hi) and Nagasaki (Na) (*NIC*=1 for being not-in-city, *NIC*=0 for being in city). The risk functions have different dose response but the same effect modifiers. The model with the linear dose response has the form:

$$EAR_{LNT}(10^{-4}PY^{-1}) = \beta_7 D \exp\left(\beta_5 \ln\frac{a}{70} + \beta_6 f\right)$$

and the model with the quadratic dose response:

$$EAR_{QDR}(10^{-4}PY^{-1}) = 1.12\beta_7 D^2 \exp\left(\beta_5 \ln\frac{a}{70} + \beta_6 f\right).$$

The variable *f* indicates females (*f*=1 for females, *f*=0 for males). These two models form a twin pair. The model parameters and their standard deviations are shown in Table S14.1.

Table S14.1: Parameters (MLE and standard deviations) of the fitted models for radiation and baseline risks of the group HEM1 leukaemias

Parameter	EAR-LNT		EAR-QDR	
	MLE	σ	MLE	σ
β_1	−2.334	0.295	−2.297	0.283
β_2	1.700	0.766	1.267	0.814
β_3	−0.020	0.567	−0.137	0.557
β_4	−0.134	1.036	−0.274	1.032
β_5	−1.810	0.372	−1.869	0.384
β_6	−0.922	0.480	−0.908	0.514
β_7	0.232	0.121	0.174	0.093

Table S14.2: Covariance matrix for the EAR-LNT model with linear non-threshold (LNT) dose response (w_{AIC} =90.18%)

	β_1	β_2	β_3	β_4	β_5	β_6	β_7
β_1	8,709E-02	9,545E-02	-7,143E-02	-6,659E-02	-1,495E-02	-3,847E-04	-5,345E-03
β_2	9,545E-02	5,867E-01	8,491E-04	3,058E-02	-1,441E-03	6,999E-03	1,357E-03
β_3	-7,143E-02	8,491E-04	3,216E-01	7,161E-02	1,471E-02	1,533E-03	5,568E-03
β_4	-6,659E-02	3,058E-02	7,161E-02	1,073E+00	1,464E-02	1,888E-03	5,637E-03
β_5	-1,495E-02	-1,441E-03	1,471E-02	1,464E-02	1,383E-01	-1,063E-02	3,673E-02
β_6	-3,847E-04	6,999E-03	1,533E-03	1,888E-03	-1,063E-02	2,305E-01	-2,055E-02
β_7	-5,345E-03	1,357E-03	5,568E-03	5,637E-03	3,673E-02	-2,055E-02	1,458E-02

Table S14.3: Covariance matrix for the EAR-QDR model with pure quadratic (QDR) dose response (w_{AIC} = 9.82%)

	β_1	β_2	β_3	β_4	β_5	β_6	β_7
β_1	8,030E-02	1,157E-01	-5,658E-02	-5,026E-02	-1,134E-02	4,979E-03	-2,384E-03
β_2	1,157E-01	6,618E-01	2,004E-02	5,623E-02	-3,069E-02	4,652E-02	-1,397E-03
β_3	-5,658E-02	2,004E-02	3,107E-01	6,179E-02	5,050E-03	4,560E-03	2,097E-03
β_4	-5,026E-02	5,623E-02	6,179E-02	1,065E+00	3,372E-03	7,103E-03	2,021E-03
β_5	-1,134E-02	-3,069E-02	5,050E-03	3,372E-03	1,472E-01	-1,675E-02	2,886E-02
β_6	4,979E-03	4,652E-02	4,560E-03	7,103E-03	-1,675E-02	2,643E-01	-1,729E-02
β_7	-2,384E-03	-1,397E-03	2,097E-03	2,021E-03	2,886E-02	-1,729E-02	8,673E-03

S15. Leukaemia group HEM2 (ICD10: C81–C86, C88, C91.1, C91.4)

The leukaemia group HEM2 includes the following diagnoses: Hodgkin lymphoma, Non-Hodgkin lymphoma, chronic lymphoblastic leukaemia (CLL), lymphoma of peripheral and cutaneous T-cell, malignant immunoproliferative disease, hairy cell leukaemia.

The group HEM2 contains a relatively large number of cancer cases – 449, including 103 'not-in-city' cases. However, fitting male and female cases separately resulted in no radiation risk for females and in a reasonably well-defined radiation risk function for males. Fitting both genders together also resulted in significant non-zero risk. Since such gender differences are not biologically plausible and also problematic for compensation claims, the decision was made to use for both genders the same set of risk models. An MMI weight of 50% is given to a set of models derived jointly for males and females, and another 50% weight to models derived from the male dataset only. Within the two groups further AIC-based weighting was performed, resulting in one ERR and one EAR model for each group. Thus, radiation risk is characterized by a set of four linear (LNT) models:

- EAR-LNT model of EAR-type (w_{AIC} = 21.00%)
- ERR-LNT model of ERR-type (w_{AIC} = 29.00%)
- EAR-LNT-male model of EAR-type (w_{AIC} = 35.87%)
- ERR-LNT-male model of ERR-type (w_{AIC} = 14.13%)

These four models are applied for the both sexes.

All selected models share the same form of the sex-specific parametric baseline:

$$\lambda_0(10^{-4}PY^{-1}) = \exp\left(\beta_1 m + \beta_2 f + \beta_3 \ln\frac{a}{70} + \beta_4 m \ln^2\frac{a}{70} + \beta_5\frac{b-1915}{10} + \right.$$
$$\left. + \beta_6\left(\frac{b-1915}{10}\right)^2 + \beta_7 NIC_{Hi} + \beta_8 NIC_{Na}\right).$$

The variable *f* indicates females (*f*=1 for females, *f*=0 for males), correspondingly for males (*m*=1 for males, *m*=0 for females), and *b* is the birthyear. *NIC* is an indicator variable for not-in-city at the time of bombing in Hiroshima (Hi) and Nagasaki (Na) (*NIC*=1 for being not-in-city, *NIC*=0 for being in city). The risk functions for all models are linear in dose without effect modifiers:

$$EAR(10^{-4}PY^{-1}) = \beta_9 D \quad \text{and} \quad ERR = \beta_9 D.$$

Since all models are linear in dose the HEM2 group has no twin models.

Table S15.1: Parameters (MLE and standard deviations) of the models selected to describe radiation risk of lymphomas including chronic lymphoblastic leukaemia (group HEM2)

Parameter	EAR-LNT		EAR-LNT-male		ERR-LNT		ERR-LNT-male	
	MLE	σ	MLE	σ	MLE	σ	MLE	σ
β_1	1.430	0.094	1.409	0.096	1.392	0.097	1.361	0.101
β_2	0.890	0.079	0.896	0.079	0.863	0.082	0.890	0.078
β_3	4.391	0.297	4.378	0.298	4.369	0.290	4.369	0.290
β_4	1.188	0.368	1.028	0.426	1.310	0.305	1.309	0.305
β_5	0.239	0.039	0.237	0.039	0.236	0.039	0.237	0.039
β_6	−0.058	0.020	−0.057	0.020	−0.056	0.020	−0.055	0.020
β_7	−0.086	0.124	−0.077	0.124	−0.065	0.126	−0.066	0.125
β_8	−0.176	0.226	−0.163	0.226	−0.155	0.227	−0.152	0.226
β_9	0.175	0.161	0.699	0.390	0.306	0.223	0.610	0.369

Table S15.2 Covariance matrix for the EAR-LNT model (w_{AIC} = 21.00%)

	β_1	β_2	β_3	β_4	β_5	β_6	β_7	β_8	β_9
β_1	8,822E-03	2,551E-03	1,874E-03	-3,465E-03	1,169E-04	-9,708E-04	-3,294E-03	-3,651E-03	-4,788E-04
β_2	2,551E-03	6,284E-03	1,507E-03	3,155E-03	1,153E-04	-6,906E-04	-3,188E-03	-2,982E-03	-1,205E-03
β_3	1,874E-03	1,507E-03	8,811E-02	5,819E-02	6,100E-03	9,297E-04	-4,166E-04	6,042E-04	2,967E-03
β_4	-3,465E-03	3,155E-03	5,819E-02	1,355E-01	1,808E-03	-1,214E-04	-1,160E-03	-3,363E-04	-1,071E-02
β_5	1,169E-04	1,153E-04	6,100E-03	1,808E-03	1,551E-03	4,506E-05	-2,351E-05	-2,196E-04	3,813E-04
β_6	-9,708E-04	-6,906E-04	9,297E-04	-1,214E-04	4,506E-05	4,142E-04	5,536E-05	1,082E-04	-2,447E-04
β_7	-3,294E-03	-3,188E-03	-4,166E-04	-1,160E-03	-2,351E-05	5,536E-05	1,534E-02	3,154E-03	1,916E-03
β_8	-3,651E-03	-2,982E-03	6,042E-04	-3,363E-04	-2,196E-04	1,082E-04	3,154E-03	5,090E-02	1,959E-03
β_9	-4,788E-04	-1,205E-03	2,967E-03	-1,071E-02	3,813E-04	-2,447E-04	1,916E-03	1,959E-03	2,594E-02

Table S15.3 Covariance matrix for the EAR-LNT-male model (w_{AIC} = 35.87%)

	β_1	β_2	β_3	β_4	β_5	β_6	β_7	β_8	β_9
β_1	9,225E-03	2,536E-03	1,499E-03	-4,895E-03	9,384E-05	-9,496E-04	-3,455E-03	-3,838E-03	-4,763E-03
β_2	2,536E-03	6,166E-03	1,786E-03	3,157E-03	1,204E-04	-6,971E-04	-3,121E-03	-2,912E-03	-4,737E-04
β_3	1,499E-03	1,786E-03	8,907E-02	6,496E-02	6,094E-03	9,562E-04	-6,123E-04	4,381E-04	2,378E-03
β_4	-4,895E-03	3,157E-03	6,496E-02	1,812E-01	1,896E-03	-2,526E-04	-1,095E-03	-1,936E-04	-2,434E-02
β_5	9,384E-05	1,204E-04	6,094E-03	1,896E-03	1,557E-03	6,593E-05	-5,672E-05	-2,575E-04	2,564E-05
β_6	-9,496E-04	-6,971E-04	9,562E-04	-2,526E-04	6,593E-05	4,052E-04	6,992E-05	1,162E-04	-1,751E-04
β_7	-3,455E-03	-3,121E-03	-6,123E-04	-1,095E-03	-5,672E-05	6,992E-05	1,533E-02	3,162E-03	3,754E-03
β_8	-3,838E-03	-2,912E-03	4,381E-04	-1,936E-04	-2,575E-04	1,162E-04	3,162E-03	5,092E-02	4,265E-03
β_9	-4,763E-03	-4,737E-04	2,378E-03	-2,434E-02	2,564E-05	-1,751E-04	3,754E-03	4,265E-03	1,523E-01

Table S15.4 Covariance matrix for the ERR-LNT model (w_{AIC} = 29.00%)

	β_1	β_2	β_3	β_4	β_5	β_6	β_7	β_8	β_9
β_1	9,338E-03	3,202E-03	2,247E-03	-2,188E-03	1,127E-04	-9,395E-04	-3,930E-03	-4,271E-03	-6,362E-03
β_2	3,202E-03	6,775E-03	1,651E-03	2,330E-03	1,334E-04	-6,833E-04	-3,713E-03	-3,512E-03	-5,853E-03
β_3	2,247E-03	1,651E-03	8,399E-02	5,277E-02	5,894E-03	8,818E-04	-5,582E-04	4,096E-04	1,264E-03
β_4	-2,188E-03	2,330E-03	5,277E-02	9,278E-02	1,928E-03	-2,022E-04	-8,719E-05	6,666E-04	9,259E-04
β_5	1,127E-04	1,334E-04	5,894E-03	1,928E-03	1,500E-03	5,323E-05	-5,379E-05	-2,419E-04	3,260E-05
β_6	-9,395E-04	-6,833E-04	8,818E-04	-2,022E-04	5,323E-05	3,861E-04	8,632E-05	1,353E-04	1,248E-04
β_7	-3,930E-03	-3,713E-03	-5,582E-04	-8,719E-05	-5,379E-05	8,632E-05	1,580E-02	3,621E-03	5,886E-03
β_8	-4,271E-03	-3,512E-03	4,096E-04	6,666E-04	-2,419E-04	1,353E-04	3,621E-03	5,136E-02	5,950E-03
β_9	-6,362E-03	-5,853E-03	1,264E-03	9,259E-04	3,260E-05	1,248E-04	5,886E-03	5,950E-03	4,995E-02

Table S15.5 Covariance matrix for the ERR-LNT-male model (w_{AIC} = 14.13%)

	β_1	β_2	β_3	β_4	β_5	β_6	β_7	β_8	β_9
β_1	1,010E-02	2,677E-03	2,261E-03	-2,170E-03	1,000E-04	-9,428E-04	-3,909E-03	-4,322E-03	-1,475E-02
β_2	2,677E-03	6,157E-03	1,761E-03	2,414E-03	1,348E-04	-6,721E-04	-3,212E-03	-3,011E-03	-2,045E-03
β_3	2,261E-03	1,761E-03	8,402E-02	5,282E-02	5,897E-03	8,817E-04	-6,262E-04	3,525E-04	1,398E-03
β_4	-2,170E-03	2,414E-03	5,282E-02	9,294E-02	1,930E-03	-2,012E-04	-1,536E-04	6,236E-04	8,775E-04
β_5	1,000E-04	1,348E-04	5,897E-03	1,930E-03	1,500E-03	5,372E-05	-5,134E-05	-2,421E-04	1,499E-04
β_6	-9,428E-04	-6,721E-04	8,817E-04	-2,012E-04	5,372E-05	3,855E-04	8,170E-05	1,298E-04	2,006E-04
β_7	-3,909E-03	-3,212E-03	-6,262E-04	-1,536E-04	-5,134E-05	8,170E-05	1,553E-02	3,369E-03	7,448E-03
β_8	-4,322E-03	-3,011E-03	3,525E-04	6,236E-04	-2,421E-04	1,298E-04	3,369E-03	5,114E-02	8,246E-03
β_9	-1,475E-02	-2,045E-03	1,398E-03	8,775E-04	1,499E-04	2,006E-04	7,448E-03	8,246E-03	1,364E-01

S16. Leukaemia group HEM3 (ICD10: C92.0, C92.2, C92.3, C92.4, C92.5, C93–C96)

The leukaemia group HEM3 includes the following diagnoses: acute myeloid leukaemia (AML), sub-acute myeloid leukaemia, myeloid sarcoma, acute promyelocytic leukaemia, acute myelomonocytic leukaemia, monocytic leukaemia, other leukaemia of specified cell type, leukaemia of unspecified cell type, other or non-specified.

The fitting resulted in four models of EAR- and ERR-types with quadratic (QDR) and threshold linear spline (TLS) dose dependencies. The models are:

- ERR-TLS model of ERR-type and threshold linear spline dose response (w_{AIC} = 6.33%);
- ERR-QDR model of ERR-type and quadratic dose response (w_{AIC} = 30.35%);
- EAR-TLS model of EAR-type and threshold linear spline dose response (w_{AIC}= 8.44%);
- EAR-QDR model of EAR-type and quadratic dose response (w_{AIC}= 54.87%).

Both models of ERR-type have the same structure of the parametric baseline rate:

$$\lambda_0(10^{-4}PY^{-1}) = \exp\left(\beta_1 m + \beta_2 f + \beta_3 \ln\frac{a}{70} + \beta_4 \ln^2\frac{a}{70} + \right.$$
$$\left. + \beta_5\frac{b-1915}{10} + \beta_6\left(\frac{b-1915}{10}\right)^2 + \beta_7 NIC_{Hi} + \beta_8 NIC_{Na}\right)$$

The variable *f* indicates females ($f = 1$ for females, $f = 0$ for males), correspondingly for males (*m*=1 for males, *m*=0 for females), and *b* is the birthyear. *NIC* is an indicator variable for not-in-city at the time of bombing in Hiroshima (Hi) and Nagasaki (Na) (*NIC*=1 for being not-in-city, *NIC*=0 for being in city). Their risk functions appear as follows with a linear spline dose response:

$$ERR_{TLS} = \exp\left(\beta_9\frac{e-30}{10} + \beta_{10}\left(\frac{e-30}{10}\right)^2 + \beta_{11} f\right) \times$$
$$\times \begin{cases} \beta_{13} D & \text{if } D < \beta_{12} \\ \beta_{12}\beta_{13} + \beta_{14}(D - \beta_{12}) & \text{otherwise} \end{cases}$$

and with a pure quadratic response:

$$ERR_{QDR} = \exp\left(\beta_9\frac{e-30}{10} + \beta_{10}\left(\frac{e-30}{10}\right)^2 + \beta_{11} f\right) \times 1.12 D^2 \beta_{12}.$$

The other two models of EAR-type also share the parametric form of the baseline rate:

$$\lambda_0(10^{-4}PY^{-1}) = \exp\left(\beta_1 m + \beta_2 f + (\beta_3 m + \beta_4 f)\ln\frac{a}{70} + (\beta_5 m + \beta_6 f)\ln^2\frac{a}{70} + \right.$$
$$\left. + \beta_7\frac{b-1915}{10} + \beta_8\left(\frac{b-1915}{10}\right)^2 + \beta_9 NIC_{Hi} + \beta_{10} NIC_{Na}\right)$$

and the risk functions of EAR-type appear as follows for a linear spline dose response:

$$EAR_{TLS}(10^{-4}PY^{-1}) = \exp\left(\beta_{11}\ln\frac{a}{70} + \beta_{12}\ln^2\frac{a}{70}\right) \times \times \begin{cases} \beta_{14}D & \text{if } D < \beta_{13} \\ \beta_{13}\beta_{14} + \beta_{15}(D-\beta_{13}) & \text{otherwise} \end{cases}$$

and for a pure quadratic dose response:

$$EAR_{QDR}(10^{-4}PY^{-1}) = \exp\left(\beta_{11}\ln\frac{a}{70} + \beta_{12}\ln^2\frac{a}{70}\right) \times 1.12D^2\beta_{13}.$$

The four selected models form two pairs of twins, one pair is formed by the ERR-TLS and ERR-QDR models, and one pair by the EAR-TLS and EAR-QDR models.

Table S16.1: Maximum likelihood estimates and standard deviations of the model parameters selected to describe radiation risk of acute myeloid leukaemia and related malignancies (group HEM3)

Para-	EAR_{TLS}		EAR_{QDR}		ERR_{TLS}		ERR_{QDR}	
meter	MLE	σ	MLE	σ	MLE	σ	MLE	σ
β_1	0.675	0.165	0.678	0.163	0.623	0.164	0.617	0.162
β_2	−0.397	0.161	−0.379	0.156	−0.319	0.157	−0.310	0.152
β_3	3.952	0.761	3.975	0.750	3.584	0.441	3.587	0.441
β_4	3.700	0.713	3.661	0.683	1.633	0.269	1.635	0.269
β_5	1.216	0.749	1.243	0.729	0.135	0.081	0.134	0.079
β_6	1.868	0.463	1.831	0.452	−0.208	0.050	−0.199	0.048
β_7	0.166	0.082	0.166	0.080	−0.313	0.242	−0.328	0.240
β_8	−0.180	0.049	−0.174	0.046	−0.184	0.393	−0.199	0.391
β_9	−0.311	0.243	−0.329	0.240	0.008	0.127	0.017	0.125
β_{10}	−0.183	0.393	−0.201	0.391	0.312	0.073	0.299	0.073
β_{11}	2.556	0.858	2.585	0.843	0.773	0.437	0.741	0.430
β_{12}	1.406	0.477	1.426	0.467	0.748	0.175	0.834	0.340
β_{13}	0.718	0.195	1.587	0.370	0.346	0.249		
β_{14}	0.704	0.456			2.809	1.260		
β_{15}	5.250	1.846						

Table S16.2: Covariance matrix for the EAR-QDR model and quadratic dose response (w_{AIC} = 54.87%).

	β_1	β_2	β_3	β_4	β_5	β_6	β_7	β_8	β_9	β_{10}	β_{11}	β_{12}	β_{13}
β_1	2,668E-02	7,230E-03	2,656E-02	1,680E-03	7,600E-03	5,830E-03	1,800E-03	-3,810E-03	-1,079E-02	-1,182E-02	-3,730E-03	-1,190E-03	-3,310E-03
β_2	7,230E-03	2,440E-02	4,300E-04	1,775E-02	4,020E-03	5,040E-03	9,900E-04	-2,700E-03	-1,016E-02	-9,040E-03	-1,150E-03	1,900E-04	-3,440E-03
β_3	2,656E-02	4,300E-04	5,620E-01	6,217E-02	4,141E-01	1,138E-02	2,267E-02	2,100E-03	-2,730E-03	-7,400E-04	-2,312E-02	-1,238E-02	-1,490E-03
β_4	1,680E-03	1,775E-02	6,217E-02	4,670E-01	2,073E-02	2,512E-01	1,626E-02	8,300E-04	4,800E-04	5,520E-03	-4,648E-02	-2,373E-02	3,850E-03
β_5	7,600E-03	4,020E-03	4,141E-01	2,073E-02	5,315E-01	8,790E-03	8,310E-03	-2,470E-03	-8,700E-04	4,200E-04	-8,040E-03	-6,410E-03	1,880E-03
β_6	5,830E-03	5,040E-03	1,138E-02	2,512E-01	8,790E-03	2,042E-01	4,070E-03	-3,100E-03	9,600E-04	2,500E-03	-1,831E-02	-1,137E-02	4,150E-03
β_7	1,800E-03	9,900E-04	2,267E-02	1,626E-02	8,310E-03	4,070E-03	6,350E-03	-2,200E-04	-1,000E-04	-8,400E-04	1,560E-03	6,000E-04	1,340E-03
β_8	-3,810E-03	-2,700E-03	2,100E-03	8,300E-04	-2,470E-03	-3,100E-03	-2,200E-04	2,150E-03	2,100E-04	4,300E-04	-3,700E-04	-1,100E-04	-7,600E-04
β_9	-1,079E-02	-1,016E-02	-2,730E-03	4,800E-04	-8,700E-04	9,600E-04	-1,000E-04	2,100E-04	5,763E-02	1,001E-02	7,200E-04	-3,100E-04	4,290E-03
β_{10}	-1,182E-02	-9,040E-03	-7,400E-04	5,520E-03	4,200E-04	2,500E-03	-8,400E-04	4,300E-04	1,001E-02	1,533E-01	-6,500E-04	-9,200E-04	3,970E-03
β_{11}	-3,730E-03	-1,150E-03	-2,312E-02	-4,648E-02	-8,040E-03	-1,831E-02	1,560E-03	-3,700E-04	7,200E-04	-6,500E-04	7,111E-01	3,638E-01	1,486E-01
β_{12}	-1,190E-03	1,900E-04	-1,238E-02	-2,373E-02	-6,410E-03	-1,137E-02	6,000E-04	-1,100E-04	-3,100E-04	-9,200E-04	3,638E-01	2,181E-01	5,326E-02
β_{13}	-3,310E-03	-3,440E-03	-1,490E-03	3,850E-03	1,880E-03	4,150E-03	1,340E-03	-7,600E-04	4,290E-03	3,970E-03	1,486E-01	5,326E-02	1,366E-01

Table S16.3: Covariance matrix for the EAR-TLS model and threshold linear spline dose response (w_{AIC} = 8.44%);

	β_1	β_2	β_3	β_4	β_5	β_6	β_7	β_8	β_9	β_{10}	β_{11}	β_{12}	β_{13}	β_{14}	β_{15}
β_1	2,737E-02	7,740E-03	2,654E-02	7,700E-04	7,440E-03	5,520E-03	1,870E-03	-3,970E-03	-1,128E-02	-1,236E-02	-2,000E-03	-2,200E-04	5,000E-04	-4,070E-03	-2,130E-03
β_2	7,740E-03	2,606E-02	-1,900E-04	1,382E-02	3,940E-03	2,810E-03	9,500E-04	-2,650E-03	-1,136E-02	-1,022E-02	3,400E-03	3,030E-03	-9,400E-04	-1,235E-02	7,100E-04
β_3	2,654E-02	-1,900E-04	5,786E-01	6,649E-02	4,282E-01	1,248E-02	2,330E-02	2,380E-03	-2,270E-03	-1,200E-04	-3,506E-02	-1,809E-02	-3,590E-03	3,440E-03	-4,900E-02
β_4	7,700E-04	1,382E-02	6,649E-02	5,079E-01	2,229E-02	2,709E-01	1,769E-02	-5,100E-04	5,620E-03	1,087E-02	-8,258E-02	-4,388E-02	1,380E-03	5,903E-02	-9,417E-02
β_5	7,440E-03	3,940E-03	4,282E-01	2,229E-02	5,613E-01	9,810E-03	8,550E-03	-2,740E-03	-6,300E-04	6,900E-04	-8,200E-03	-6,690E-03	1,400E-04	5,100E-03	-2,010E-03
β_6	5,520E-03	2,810E-03	1,248E-02	2,709E-01	9,810E-03	2,140E-01	4,750E-03	-4,120E-03	3,940E-03	5,510E-03	-3,512E-02	-2,130E-02	1,910E-03	3,474E-02	-3,394E-02
β_7	1,870E-03	9,500E-04	2,330E-02	1,769E-02	8,550E-03	4,750E-03	6,720E-03	-3,600E-04	1,400E-04	-6,500E-04	1,050E-03	2,100E-04	2,500E-04	3,540E-03	1,100E-03
β_8	-3,970E-03	-2,650E-03	2,380E-03	-5,100E-04	-2,740E-03	-4,120E-03	-3,600E-04	2,440E-03	-1,200E-04	1,300E-04	5,000E-05	3,200E-04	-6,800E-04	-4,270E-03	-1,820E-03
β_9	-1,128E-02	-1,136E-02	-2,270E-03	5,620E-03	-6,300E-04	3,940E-03	1,400E-04	-1,200E-04	5,907E-02	1,144E-02	-4,420E-03	-3,540E-03	9,800E-04	1,539E-02	-1,820E-03
β_{10}	-1,236E-02	-1,022E-02	-1,200E-04	1,087E-02	6,900E-04	5,510E-03	-6,500E-04	1,300E-04	1,144E-02	1,547E-01	-6,330E-03	-4,370E-03	7,700E-04	1,503E-02	-4,500E-03
β_{11}	-2,000E-03	3,400E-03	-3,506E-02	-8,258E-02	-8,200E-03	-3,512E-02	1,050E-03	5,000E-05	-4,420E-03	-6,330E-03	7,368E-01	3,790E-01	8,780E-03	4,320E-03	6,698E-01
β_{12}	-2,200E-04	3,030E-03	-1,809E-02	-4,388E-02	-6,690E-03	-2,130E-02	2,100E-04	3,200E-04	-3,540E-03	-4,370E-03	3,790E-01	2,272E-01	1,970E-03	-1,474E-02	2,637E-01
β_{13}	5,000E-04	-9,400E-04	-3,590E-03	1,380E-03	1,400E-04	1,910E-03	2,500E-04	-6,800E-04	9,800E-04	7,700E-04	8,780E-03	1,970E-03	3,822E-02	3,040E-02	2,186E-01
β_{14}	-4,070E-03	-1,235E-02	3,440E-03	5,903E-02	5,100E-03	3,474E-02	3,540E-03	-4,270E-03	1,539E-02	1,503E-02	4,320E-03	-1,474E-02	3,040E-02	2,080E-01	4,527E-02
β_{15}	-2,130E-03	7,100E-04	-4,900E-02	-9,417E-02	-2,010E-03	-3,394E-02	1,100E-03	-1,820E-03	-1,820E-03	-4,500E-03	6,698E-01	2,637E-01	2,186E-01	4,527E-02	3,409E+00

Table S16.4: Covariance matrix for the ERR-QDR model and quadratic dose response (w_{AIC} = 30.35%);

	β_1	β_2	β_3	β_4	β_5	β_6	β_7	β_8	β_9	β_{10}	β_{11}	β_{12}
β_1	2,637E-02	8,590E-03	1,419E-02	6,990E-03	1,720E-03	-4,140E-03	-1,075E-02	-1,168E-02	-1,600E-04	4,990E-03	2,100E-02	-2,604E-02
β_2	8,590E-03	2,325E-02	7,240E-03	3,640E-03	1,540E-03	-2,920E-03	-1,032E-02	-9,340E-03	8,200E-04	3,690E-03	-1,906E-02	-8,680E-03
β_3	1,419E-02	7,240E-03	1,946E-01	9,685E-02	1,484E-02	1,560E-03	-1,550E-03	1,000E-03	1,870E-03	-1,640E-03	3,240E-03	5,840E-03
β_4	6,990E-03	3,640E-03	9,685E-02	7,239E-02	5,060E-03	-1,090E-03	-3,200E-04	6,700E-04	3,890E-03	-8,800E-04	1,570E-03	3,390E-03
β_5	1,720E-03	1,540E-03	1,484E-02	5,060E-03	6,300E-03	-5,600E-04	-7,000E-05	-9,000E-04	5,520E-03	8,300E-04	-1,590E-03	4,700E-04
β_6	-4,140E-03	-2,920E-03	1,560E-03	-1,090E-03	-5,600E-04	2,320E-03	2,400E-04	4,800E-04	-9,300E-04	-2,480E-03	-9,200E-04	3,960E-03
β_7	-1,075E-02	-1,032E-02	-1,550E-03	-3,200E-04	-7,000E-05	2,400E-04	5,763E-02	9,970E-03	4,500E-04	-6,100E-04	-7,000E-04	1,201E-02
β_8	-1,168E-02	-9,340E-03	1,000E-03	6,700E-04	-9,000E-04	4,800E-04	9,970E-03	1,532E-01	-7,300E-04	-8,400E-04	-2,850E-03	1,308E-02
β_9	-1,600E-04	8,200E-04	1,870E-03	3,890E-03	5,520E-03	-9,300E-04	4,500E-04	-7,300E-04	1,564E-02	-1,200E-04	-5,400E-03	6,710E-03
β_{10}	4,990E-03	3,690E-03	-1,640E-03	-8,800E-04	8,300E-04	-2,480E-03	-6,100E-04	-8,400E-04	-1,200E-04	5,390E-03	2,990E-03	-1,424E-02
β_{11}	2,100E-02	-1,906E-02	3,240E-03	1,570E-03	-1,590E-03	-9,200E-04	-7,000E-04	-2,850E-03	-5,400E-03	2,990E-03	1,853E-01	-9,012E-02
β_{12}	-2,604E-02	-8,680E-03	5,840E-03	3,390E-03	4,700E-04	3,960E-03	1,201E-02	1,308E-02	6,710E-03	-1,424E-02	-9,012E-02	1,154E-01

Table S16.5: Covariance matrix for the ERR-TLS model and threshold linear spline dose response (w_{AIC} = 6.33%);

	β_1	β_2	β_3	β_4	β_5	β_6	β_7	β_8	β_9	β_{10}	β_{11}	β_{12}	β_{13}	β_{14}
β_1	2,704E-02	8,830E-03	1,413E-02	6,890E-03	1,720E-03	-4,280E-03	-1,117E-02	-1,213E-02	-2,600E-04	0,00524	2,281E-02	1,490E-03	-1,223E-02	-8,633E-02
β_2	8,830E-03	2,466E-02	7,220E-03	3,620E-03	1,590E-03	-2,780E-03	-1,138E-02	-1,034E-02	1,140E-03	0,00374	-2,071E-02	-1,600E-04	-8,600E-03	-2,434E-02
β_3	1,413E-02	7,220E-03	1,945E-01	9,681E-02	1,488E-02	1,640E-03	-1,640E-03	9,100E-04	1,990E-03	-0,00169	3,340E-03	-3,000E-05	1,450E-03	2,091E-02
β_4	0,00689	0,00362	0,09681	0,07243	0,00519	-0,00103	-0,00037	0,0006	0,00417	-0,00093	0,00128	-0,0002	0,0011	0,01169
β_5	1,720E-03	1,590E-03	1,488E-02	5,190E-03	6,590E-03	-6,100E-04	-6,000E-05	-9,300E-04	5,960E-03	8,200E-04	-0,00188	3,900E-04	6,800E-04	2,780E-03
β_6	-4,280E-03	-2,780E-03	1,640E-03	-1,030E-03	-6,100E-04	2,530E-03	0,000E+00	2,600E-04	-8,600E-04	-2,630E-03	-0,00133	-5,400E-04	-3,000E-05	1,447E-02
β_7	-1,117E-02	-1,138E-02	-1,640E-03	-3,700E-04	-6,000E-05	0,000E+00	5,873E-02	1,104E-02	1,700E-04	-6,200E-04	-0,00035	1,000E-04	1,061E-02	3,628E-02
β_8	-1,213E-02	-1,034E-02	9,100E-04	6,000E-04	-9,300E-04	2,600E-04	1,104E-02	1,542E-01	-1,070E-03	-8,500E-04	-0,00265	-7,000E-05	1,073E-02	3,986E-02
β_9	-2,600E-04	1,140E-03	1,990E-03	4,170E-03	5,960E-03	-8,600E-04	1,700E-04	-1,070E-03	1,614E-02	-2,600E-04	-0,00649	9,500E-04	1,910E-03	2,913E-02
β_{10}	5,240E-03	3,740E-03	-1,690E-03	-9,300E-04	8,200E-04	-2,630E-03	-6,200E-04	-8,500E-04	-2,600E-04	5,360E-03	0,00353	5,800E-04	-5,700E-03	-4,599E-02
β_{11}	2,281E-02	-2,071E-02	3,340E-03	1,280E-03	-1,880E-03	-1,330E-03	-3,500E-04	-2,650E-03	-6,490E-03	3,530E-03	0,19121	7,610E-03	-3,598E-02	-2,887E-01
β_{12}	1,490E-03	-1,600E-04	-3,000E-05	-2,000E-04	3,900E-04	-5,400E-04	1,000E-04	-7,000E-05	9,500E-04	5,800E-04	0,00761	3,059E-02	9,130E-03	7,772E-02
β_{13}	-1,223E-02	-8,600E-03	1,450E-03	1,100E-03	6,800E-04	-3,000E-05	1,061E-02	1,073E-02	1,910E-03	-5,700E-03	-0,03598	9,130E-03	6,218E-02	1,344E-01
β_{14}	-8,633E-02	-2,434E-02	2,091E-02	1,169E-02	2,780E-03	1,447E-02	3,628E-02	3,986E-02	2,913E-02	-4,599E-02	-0,28871	7,772E-02	1,344E-01	1,587E+00

S17. Leukaemia group HEM4 (ICD10: C92.1)

The leukaemia group HEM4 includes only chronic myeloid leukaemia (CML).

The fitting resulted in a group of six models, four of ERR-type and two of EAR-type. These models are:

- ERR-t-QE model of ERR-type with quadratic-exponential dose response and time-since-exposure effect modifier (w_{AIC} = 7.08%);
- ERR-t-LNT model of ERR-type with linear dose response and time-since-exposure effect modifier (w_{AIC} = 21.00%);
- ERR-e-QE model of ERR-type with quadratic-exponential dose response and age-at-exposure effect modifier (w_{AIC} = 1.72%);
- ERR-e-LNT model of ERR-type with linear dose response and age-at-exposure effect modifier (w_{AIC} = 7.00%);
- EAR-t-LNT model of EAR-type with linear dose response and time-since-exposure effect modifier (w_{AIC} = 55.12%);
- EAR-e-LNT model of EAR-type with linear dose response and age-at-exposure effect modifier (w_{AIC} = 8.08%).

The four models of ERR-type share the same form of the parametric baseline rate:

$$\lambda_0(10^{-4}PY^{-1}) = \exp\left(\beta_1 + (\beta_2 m + \beta_3 f)\ln\frac{a}{70} + \beta_4 NIC_{Hi} + \beta_5 NIC_{Na}\right).$$

The variable *f* indicates females (*f*=1 for females, *f*=0 for males), and correspondingly for males (*m*=1 for males, *m*=0 for females). *NIC* is an indicator variable for not-in-city at the time of bombing in Hiroshima (Hi) and Nagasaki (Na) (*NIC*=1 for being not-in-city, *NIC*=0 for being in city). For the ERR-type models, the risk functions appear as follows:

$$ERR_{tQE} = \exp\left(\beta_6 \ln\frac{a}{55} + \frac{\beta_7}{10}\ln\frac{a-e}{25} + \beta_8 n\right) \times 1.12\beta_9 D^2 \exp\left(\beta_{10} D\right),$$

$$ERR_{tLNT} = \exp\left(\beta_6 \ln\frac{a}{55} + \frac{\beta_7}{10}\ln\frac{a-e}{25} + \beta_8 n\right) \times \beta_9 D$$

if time since exposure is selected as an effect modifier for risk, and

$$ERR_{eQE} = \exp\left(\beta_6 \ln\frac{a}{55} + \beta_7 \ln^2\frac{a}{55} + \beta_8\frac{e-30}{10} + \beta_9 n\right) \times 1.12\beta_{10} D^2 \exp\left(\beta_{11} D\right),$$

$$ERR_{eLNT} = \exp\left(\beta_6 \ln\frac{a}{55} + \beta_7 \ln^2\frac{a}{55} + \beta_8\frac{e-30}{10} + \beta_9 n\right) \times \beta_{10} D$$

if age at exposure is used to modify radiation risk. The variable *n* indicates the city (*n*=1 for Nagasaki, *n*=0 for Hiroshima).

The two models of EAR-type also have their common, sex-independent, structure of simple parametric baseline:

$$\lambda_0(10^{-4}PY^{-1}) = \exp\left(\beta_1 + \beta_2 \ln\frac{a}{70} + \beta_3 NIC_{Hi} + \beta_4 NIC_{Na}\right).$$

Radiation-attributed excess rates for the EAR-type models appear as follows:

$$EAR_{tLNT}\,(10^{-4}PY^{-1}) = \exp\left(\beta_5 f \ln\frac{a}{55} + \frac{\beta_6}{10}\ln\frac{a-e}{25}\right) \times \beta_7 D,$$

$$EAR_{eLNT}\,(10^{-4}PY^{-1}) = \exp\left((\beta_5 m + \beta_6 f)\ln\frac{a}{55} + \beta_7 \ln^2\frac{a}{55} + \beta_8\frac{e-30}{10}\right) \times \beta_9 D.$$

Of the six models belonging to the group HEM4, the four models of ERR-type are treated as twin models. One pair of models is formed by the ERR-t-QE and ERR-t-LNT models, and one pair by the ERR-e-QE and ERR-e-LNT models.

Table S17.1: Maximum likelihood estimates and standard deviations of the model parameters selected to describe radiation risk of chronic myeloid leukaemia (group HEM4)

Parameter	EAR_{tLNT}		EAR_{eLNT}		ERR_{tLNT}		ERR_{tQE}		ERR_{eLNT}		ERR_{eQE}	
	MLE	σ	MLE	σ	MLE	σ	MLE	σ	MLE	σ	MLE	σ
β_1	−1.551	0.210	−1.535	0.210	−1.554	0.207	−1.472	0.193	−1.555	0.208	−1.474	0.194
β_2	2.284	0.662	2.358	0.671	1.322	0.615	1.200	0.546	1.361	0.627	1.212	0.545
β_3	−0.162	0.427	−0.169	0.426	3.149	0.679	3.023	0.636	3.189	0.702	3.036	0.645
β_4	−0.807	1.020	−0.811	1.020	−0.188	0.425	−0.286	0.418	−0.182	0.425	−0.282	0.418
β_5	2.187	0.932	−5.658	1.588	−0.835	1.019	−0.939	1.016	−0.827	1.019	−0.935	1.016
β_6	−16.650	3.330	−3.257	1.597	−1.374	0.731	−1.336	0.671	−6.545	1.647	−6.648	1.662
β_7	0.599	0.203	−1.762	0.794	−15.951	3.443	−16.678	3.668	−1.551	0.746	−1.570	0.759
β_8			1.005	0.262	−1.499	0.744	−1.710	0.757	0.966	0.255	1.006	0.274
β_9			0.608	0.239	5.275	2.333	10.533	6.311	−1.489	0.741	−1.707	0.756
β_{10}							−0.752	0.289	5.643	2.700	11.347	7.125
β_{11}											−0.761	0.288

Table S17.2: Covariance matrix for the EAR-t-LNT model with linear dose response and time-since-exposure effect modifier (w_{AIC} = 55.12%);

	β_1	β_2	β_3	β_4	β_5	β_6	β_7
β_1	4,428E-02	4,747E-02	-3,864E-02	-3,643E-02	-1,054E-02	-1,082E-01	-9,330E-03
β_2	4,747E-02	4,379E-01	4,540E-03	2,494E-02	-7,265E-02	5,400E-02	8,370E-03
β_3	-3,864E-02	4,540E-03	1,820E-01	3,939E-02	1,910E-03	1,147E-01	1,032E-02
β_4	-3,643E-02	2,494E-02	3,939E-02	1,041E+00	-1,470E-03	1,172E-01	1,071E-02
β_5	-1,054E-02	-7,265E-02	1,910E-03	-1,470E-03	8,685E-01	-6,120E-01	-2,284E-02
β_6	-1,082E-01	5,400E-02	1,147E-01	1,172E-01	-6,120E-01	1,109E+01	5,469E-01
β_7	-9,330E-03	8,370E-03	1,032E-02	1,071E-02	-2,284E-02	5,469E-01	4,107E-02

Table S17.3: Covariance matrix for the EAR-e-LNT model with linear dose response and age-at-exposure effect modifier (w_{AIC} = 8.08%).

	β_1	β_2	β_3	β_4	β_5	β_6	β_7	β_8	β_9
β_1	4,413E-02	4,806E-02	-3,866E-02	-3,645E-02	-6,774E-02	-6,362E-02	-2,728E-02	8,340E-03	-1,197E-02
β_2	4,806E-02	4,506E-01	3,180E-03	2,398E-02	-5,278E-02	-9,059E-02	-4,356E-02	-2,450E-03	5,720E-03
β_3	-3,866E-02	3,180E-03	1,819E-01	3,917E-02	6,174E-02	5,331E-02	2,233E-02	-8,620E-03	1,263E-02
β_4	-3,645E-02	2,398E-02	3,917E-02	1,040E+00	5,931E-02	4,913E-02	2,032E-02	-8,730E-03	1,289E-02
β_5	-6,774E-02	-5,278E-02	6,174E-02	5,931E-02	2,523E+00	1,948E+00	1,107E+00	-3,474E-01	2,833E-01
β_6	-6,362E-02	-9,059E-02	5,331E-02	4,913E-02	1,948E+00	2,551E+00	7,740E-01	-3,279E-01	2,498E-01
β_7	-2,728E-02	-4,356E-02	2,233E-02	2,032E-02	1,107E+00	7,740E-01	6,302E-01	-1,153E-01	8,487E-02
β_8	8,340E-03	-2,450E-03	-8,620E-03	-8,730E-03	-3,474E-01	-3,279E-01	-1,153E-01	6,875E-02	-4,960E-02
β_9	-1,197E-02	5,720E-03	1,263E-02	1,289E-02	2,833E-01	2,498E-01	8,487E-02	-4,960E-02	5,700E-02

Table S17.4: Covariance matrix for the ERR-t-LNT model with linear dose response and time-since-exposure effect modifier (w_{AIC} = 21.00%);

	β_1	β_2	β_3	β_4	β_5	β_6	β_7	β_8	β_9
β_1	4,281E-02	4,434E-02	3,703E-02	-3,739E-02	-3,520E-02	-5,103E-02	-9,287E-02	3,210E-03	-2,609E-01
β_2	4,434E-02	3,786E-01	2,608E-01	2,800E-04	1,775E-02	-3,917E-01	4,188E-02	3,373E-02	1,867E-01
β_3	3,703E-02	2,608E-01	4,610E-01	2,190E-03	2,045E-02	-2,836E-01	-7,800E-04	2,510E-02	2,583E-01
β_4	-3,739E-02	2,800E-04	2,190E-03	1,803E-01	3,754E-02	4,440E-03	9,691E-02	8,200E-04	2,868E-01
β_5	-3,520E-02	1,775E-02	2,045E-02	3,754E-02	1,039E+00	-1,393E-02	9,819E-02	2,420E-03	2,983E-01
β_6	-5,103E-02	-3,917E-01	-2,836E-01	4,440E-03	-1,393E-02	5,343E-01	-3,980E-01	-2,323E-02	-8,418E-02
β_7	-9,287E-02	4,188E-02	-7,800E-04	9,691E-02	9,819E-02	-3,980E-01	1,186E+01	-1,044E-01	5,032E+00
β_8	3,210E-03	3,373E-02	2,510E-02	8,200E-04	2,420E-03	-2,323E-02	-1,044E-01	5,541E-01	-1,984E-01
β_9	-2,609E-01	1,867E-01	2,583E-01	2,868E-01	2,983E-01	-8,418E-02	5,032E+00	-1,984E-01	5,441E+00

Table S17.5: Covariance matrix for the ERR-e-LNT model with linear dose response and age-at-exposure effect modifier (W_{AIC} = 7.00%);

	β_1	β_2	β_3	β_4	β_5	β_6	β_7	β_8	β_9	β_{10}
β_1	4,333E-02	4,515E-02	4,024E-02	-3,788E-02	-3,561E-02	-1,092E-01	-2,496E-02	8,260E-03	2,870E-03	-2,946E-01
β_2	4,515E-02	3,934E-01	2,797E-01	3,300E-04	1,855E-02	-4,306E-01	-1,833E-02	1,280E-03	3,763E-02	1,963E-01
β_3	4,024E-02	2,797E-01	4,932E-01	6,200E-04	2,012E-02	-4,225E-01	-9,793E-02	1,390E-03	2,613E-02	2,857E-01
β_4	-3,788E-02	3,300E-04	6,200E-04	1,808E-01	3,795E-02	5,604E-02	2,038E-02	-8,100E-03	1,460E-03	3,215E-01
β_5	-3,561E-02	1,855E-02	2,012E-02	3,795E-02	1,039E+00	3,351E-02	1,765E-02	-8,030E-03	3,190E-03	3,338E-01
β_6	-1,092E-01	-4,306E-01	-4,225E-01	5,604E-02	3,351E-02	2,712E+00	9,861E-01	-3,256E-01	-5,154E-02	2,422E+00
β_7	-2,496E-02	-1,833E-02	-9,793E-02	2,038E-02	1,765E-02	9,861E-01	5,559E-01	-1,066E-01	8,200E-04	7,437E-01
β_8	8,260E-03	1,280E-03	1,390E-03	-8,100E-03	-8,030E-03	-3,256E-01	-1,066E-01	6,495E-02	7,550E-03	-4,760E-01
β_9	2,870E-03	3,763E-02	2,613E-02	1,460E-03	3,190E-03	-5,154E-02	8,200E-04	7,550E-03	5,495E-01	-2,096E-01
β_{10}	-2,946E-01	1,963E-01	2,857E-01	3,215E-01	3,338E-01	2,422E+00	7,437E-01	-4,760E-01	-2,096E-01	7,287E+00

Table S17.6: Covariance matrix for the ERR-t-QE model with quadratic-exponential dose response and time-since-exposure effect modifier (w_{AIC} = 7.08%);

	β_1	β_2	β_3	β_4	β_5	β_6	β_7	β_8	β_9	β_{10}
β_1	3,728E-02	3,961E-02	3,416E-02	-3,201E-02	-2,999E-02	-4,443E-02	-6,963E-02	5,770E-03	-5,149E-01	6,410E-03
β_2	3,961E-02	2,981E-01	1,940E-01	-2,050E-03	1,176E-02	-3,049E-01	2,381E-02	1,115E-02	2,606E-01	-3,310E-03
β_3	3,416E-02	1,940E-01	4,051E-01	-3,200E-04	1,476E-02	-2,103E-01	-1,377E-02	4,650E-03	4,777E-01	-7,120E-03
β_4	-3,201E-02	-2,050E-03	-3,200E-04	1,746E-01	3,171E-02	5,610E-03	7,165E-02	-4,450E-03	5,581E-01	-6,990E-03
β_5	-2,999E-02	1,176E-02	1,476E-02	3,171E-02	1,032E+00	-8,780E-03	7,211E-02	-3,990E-03	5,769E-01	-7,250E-03
β_6	-4,443E-02	-3,049E-01	-2,103E-01	5,610E-03	-8,780E-03	4,508E-01	-4,407E-01	-1,370E-03	-8,726E-02	6,580E-03
β_7	-6,963E-02	2,381E-02	-1,377E-02	7,165E-02	7,211E-02	-4,407E-01	1,345E+01	-1,319E-01	1,227E+01	-9,743E-02
β_8	5,770E-03	1,115E-02	4,650E-03	-4,450E-03	-3,990E-03	-1,370E-03	-1,319E-01	5,727E-01	-6,184E-01	4,650E-03
β_9	-5,149E-01	2,606E-01	4,777E-01	5,581E-01	5,769E-01	-8,726E-02	1,227E+01	-6,184E-01	3,982E+01	-1,242E+00
β_{10}	6,410E-03	-3,310E-03	-7,120E-03	-6,990E-03	-7,250E-03	6,580E-03	-9,743E-02	4,650E-03	-1,242E+00	8,374E-02

Table S17.7: Covariance matrix for the ERR-e-QE model with quadratic-exponential dose response and age-at-exposure effect modifier (w_{AIC} = 1.72%);

	β_1	β_2	β_3	β_4	β_5	β_6	β_7	β_8	β_9	β_{10}	β_{11}
β_1	3,767E-02	3,985E-02	3,580E-02	-3,235E-02	-3,029E-02	-8,531E-02	-1,653E-02	6,150E-03	5,170E-03	-5,814E-01	6,400E-03
β_2	3,985E-02	2,975E-01	1,949E-01	-2,590E-03	1,119E-02	-3,036E-01	2,900E-03	1,390E-03	1,083E-02	2,248E-01	-2,270E-03
β_3	3,580E-02	1,949E-01	4,162E-01	-1,780E-03	1,356E-02	-3,039E-01	-7,457E-02	9,700E-04	-3,000E-05	4,949E-01	-6,920E-03
β_4	-3,235E-02	-2,590E-03	-1,780E-03	1,749E-01	3,190E-02	4,379E-02	1,434E-02	-5,980E-03	-4,050E-03	6,211E-01	-6,860E-03
β_5	-3,029E-02	1,119E-02	1,356E-02	3,190E-02	1,033E+00	2,743E-02	1,280E-02	-5,910E-03	-3,700E-03	6,391E-01	-7,090E-03
β_6	-8,531E-02	-3,036E-01	-3,039E-01	4,379E-02	2,743E-02	2,764E+00	1,027E+00	-3,683E-01	-1,888E-02	5,925E+00	-2,511E-02
β_7	-1,653E-02	2,900E-03	-7,457E-02	1,434E-02	1,280E-02	1,027E+00	5,763E-01	-1,180E-01	1,132E-02	1,706E+00	-5,600E-03
β_8	6,150E-03	1,390E-03	9,700E-04	-5,980E-03	-5,910E-03	-3,683E-01	-1,180E-01	7,507E-02	9,450E-03	-1,154E+00	6,860E-03
β_9	5,170E-03	1,083E-02	-3,000E-05	-4,050E-03	-3,700E-03	-1,888E-02	1,132E-02	9,450E-03	5,716E-01	-6,752E-01	4,930E-03
β_{10}	-5,814E-01	2,248E-01	4,949E-01	6,211E-01	6,391E-01	5,925E+00	1,706E+00	-1,154E+00	-6,752E-01	5,076E+01	-1,334E+00
β_{11}	6,400E-03	-2,270E-03	-6,920E-03	-6,860E-03	-7,090E-03	-2,511E-02	-5,600E-03	6,860E-03	4,930E-03	-1,334E+00	8,318E-02

S18. Lung cancer after exposure to radon in mines

Currently, the implemented model is based on a study of the German Wismut miner cohort (Kreuzer et al. 2015). The Wismut cohort is the worldwide largest epidemiological cohort of miners exposed to radon. Furthermore, the cohort is relevant for compensation claims after radon exposure in mines in Germany.

The model selected for ProZES was developed for a sub-cohort of Wismut workers hired in 1960 or later, when dosimetric control and safety in workplaces were significantly improved compared to the preceding period, thus resulting in generally lower and better quantified estimates of exposures. Exposure is given in terms of working level month, WLM (ICRP 2010).

The parametric baseline has the following form:

$$\lambda_0 = \exp\left(\beta_1 + \beta_2(cy - 1973) + \beta_3 \ln\frac{a}{70} + \beta_4 \max^2\left(0, \ln\frac{a}{\beta_5}\right)\right),$$

cy is the calendar year. Radiation risk is described by a simple ERR model with a linear dose response without effect modifiers, where D is the total exposure in WLM:

$$ERR = \beta_6 \cdot D.$$

Table S18.1: Parameters of the lung cancer risk model after exposure to radon for miners (Kreuzer et al. 2015)

Parameter	Unit	MLE	σ	95%CI
β_1	none	−4.635	0.235	(−5.096;−4.174)[a]
β_2	yr^{-1}	−0.0251	0.0066	(−0.0379; −0.0123)[a]
β_3	none	7.650	0.427	(6.814; 8.486)[a]
β_4	none	−15.57	4.67	(−2472; −6.41)[a]
β_5	yr	60[b]	—	—
β_6	WLM^{-1}	0.0130	0.0033	(0.0072; 0.0206)[c]

[a] Wald-type CI
[b] optimised separately (manual fit)
[c] log-likelihood profile-based CI

S19. Lung cancer after indoor exposure to radon

The model for lung cancer after indoor exposure to radon and progeny was defined using results of the study of Darby et al. (2005). It is based on a pooled analysis using data from 13 European case-control studies of lung cancer after residential radon exposure.

Radon exposure is quantified by indoor air activity concentration (Bq m^{-3}) times duration of exposure. According to Darby et al. (2006, Table B15), the average percentage of time spent indoors at home among cases was approximately 60%. Correspondingly, the excess relative risk value reported in this study (0.16 per 100 Bq m^{-3}) is attributed to 30 years of residential exposure to radon in air at concentration of 100 Bq m^{-3} with average indoor occupancy 60%, which corresponds to the cumulative exposure time of 157788 hours.

Thus, the required input includes both the average radon activity concentration in air q (Bq m^{-3}) and the duration of indoor exposure T (hours). Concerning the duration of exposure, it is important to note that the radon indoor model is different from all other ProZES models. In other models the exposure duration is only used to estimate the dose rate and correct for a potential low dose rate effect, which might increase in particular the error bounds of assigned share. In contrast, for the indoor model the total exposure is directly proportional to exposure duration, and risk increases linearly with duration. For example, an average annual working time in Germany in the period 2000–2016 accounted for approximately 1400 hours (OECD.Stat, https://stats.oecd.org/Index.aspx?DataSetCode=ANHRS).

The excess relative risk of lung cancer after indoor radon exposure is therefore estimated as follows:

$$ERR = \beta_m \cdot q \cdot \frac{T}{30 \cdot 365.25 \cdot 24 \cdot 0.6} = \frac{\beta_m \cdot q \cdot T}{157788},$$

where $\beta_m = 1.6 \text{ kBq}^{-1} \text{ m}^3$ is the risk coefficient from Darby et al. (2005) with 95% confidence interval $(0.5 - 3.1) \text{ kBq}^{-1} \text{ m}^3$. The following equation is used in ProZES to compute the excess relative risk:

$$ERR = \beta \cdot q \cdot T\,,$$

where the parameter β is sampled from a Gaussian distribution with the following parameter values:

$$\mu(\text{Bq}^{-1} \text{ m}^3 \text{ h}^{-1}) = 1.01 \cdot 10^{-8}, \text{and } \sigma(\text{Bq}^{-1} \text{ m}^3 \text{ h}^{-1}) = 0.42 \cdot 10^{-8}\,.$$

References

Darby S, Hill D, Auvinen A, Barros-Dios JM, Baysson H, Bochicchio F, Deo H, Falk R, Forastiere F, Hakama M, Heid I, Kreienbrock L, Kreuzer M, Lagarde F, Mäkeläinen I, Muirhead C, Oberaigner W, Pershagen G, Ruano-Ravina A, Ruosteenoja E, Rosario AS, Tirmarche M, Tomásek L, Whitley E, Wichmann HE, Doll R (2005) Radon in homes and risk of lung cancer: collaborative analysis of individual data from 13 European case-control studies. BMJ 330(7485):223

Darby S, Hill D, Deo H, Auvinen A, Barros-Dios JM, Baysson H, Bochicchio F, Falk R, Farchi S, Figueiras A, Hakama M, Heid I, Hunter N, Kreienbrock L, Kreuzer M, Lagarde F, Mäkeläinen I, Muirhead C, Oberaigner W, Pershagen G, Ruosteenoja E, Rosario AS, Tirmarche M, Tomasek L, Whitley E, Wichmann HE, Doll R (2006) Residential radon and lung cancer - detailed results of a collaborative analysis of individual data on 7148 persons with lung cancer and 14208 persons without lung cancer from 13 epidemiologic studies in Europe. Scand J Work Environ Health 32 Suppl 1:1-83.

Furukawa K, Preston DL, Lönn S, Funamoto S, Yonehara S, Matsuo T, Egawa H, Tokuoka S, Ozasa K, Kasagi F, Kodama K, Mabuchi K (2010) Radiation and smoking effects on lung cancer incidence among atomic bomb survivors. Radiat Res 174:72-82

ICRP (2010) Lung Cancer Risk from Radon and Progeny and Statement on Radon. ICRP Publication 115. Ann ICRP 40:1-64

Jacob P, Kaiser J, Ulanowski A (2013) ProZES – a tool for assessment of assigned share of radiation in probability of cancer development. Research report, Institute of Radiation Protection, Helmholtz Zentrum München - German Research Center for Environmental Health, Neuherberg, www.bfs.de/prozes

Jacob P, Kaiser JC, Ulanovsky A. (2014) Ultrasonography survey and thyroid cancer in the Fukushima Prefecture. Radiat Environ Biophys 53:391-401. Erratum in: Radiat Environ Biophys 53:403

Kreuzer M, Fenske N, Schnelzer M, Walsh L (2015) Lung cancer risk at low radon exposure rates in German uranium miners. Br J Cancer 113:1367-9

Preston DL, Mattsson A, Holmberg E, Shore R, Hildreth NG, Boice JD (2002) Radiation effects on breast cancer risk: a pooled analysis of eight cohorts. Radiat Res 158:220-35. Erratum in: Radiat Res 158:666

Schulze A, Lampert T (2006) Bundes-Gesundheitssurvey: Soziale Unterschiede im Rauchverhalten und in der Passivrauchbelastung in Deutschland, RKI, Berlin

Ulanowski A, Eidemüller M, Güthlin D, Kaiser JC, Shemiakina E, Jacob P (2016) ProZES – a tool for assessment of assigned share of radiation in probability of cancer development (Part II). Research report, Institute of Radiation Protection, Helmholtz Zentrum München - German Research Center for Environmental Health, Neuherberg, www.bfs.de/prozes
